# Grapevine microRNAs responsive to exogenous gibberellin

**DOI:** 10.1186/1471-2164-15-111

**Published:** 2014-02-08

**Authors:** Jian Han, Jinggui Fang, Chen Wang, Yanlei Yin, Xin Sun, Xiangpeng Leng, Changnian Song

**Affiliations:** 1 College of Horticulture, Nanjing Agricultural University, Nanjing 210095, China; 2 Institute of Pomology, Taian 210038, Shandong, China

**Keywords:** Grapevine, Berry, microRNAs, Exogenous gibberellin, High throughput sequencing

## Abstract

**Background:**

MicroRNAs (miRNAs), involving in various biological and metabolic processes, have been discovered and analyzed in quite a number of plants species, such as *Arabidopsis*, rice and other plants. However, there have been few reports about grapevine miRNAs in response to gibberelline (GA_3_).

**Results:**

Solexa technology was used to sequence small RNA libraries constructed from grapevine berries treated with GA_3_ and the control. A total of 122 known and 90 novel grapevine miRNAs (Vvi-miRNAs) were identified. Totally, 137 ones were found to be clearly responsive to GA_3_, among which 58 were down-regulated, 51 were up-regulated, 21 could only be detected in the control, and seven were only detected in the treatment. Subsequently, we found that 28 of them were differentially regulated by GA_3_, with 12 conserved and 16 novel Vvi-miRNAs, based on the analysis of qRT-PCR essays. There existed some consistency in expression levels of GA_3_-responsive Vvi-miRNAs between high throughput sequencing and qRT-PCR essays. In addition, 117 target genes for 29 novel miRNAs were predicted.

**Conclusions:**

Deep sequencing of short RNAs from grapevine berries treated with GA3 and the control identified 137 GA_3_-responsive miRNAs, among which 28 exhibited different expression profiles of response to GA_3_ in the diverse developmental stages of grapevine berries. These identified Vvi-miRNAs might be involved in the grapevine berry development and response to environmental stresses.

## Background

MicroRNAs (miRNAs) are endogenous gene regulators distributed widely in plant genomes, and they play important roles in plant growth, development, signal transduction and response to environmental stimuli [1-5]. Identification of miRNAs is a key step in gaining insight into sRNA-based regulatory functions with many conserved miRNAs having been identified by traditional sequencing approaches such as the Sanger sequencing method [6]. However, most species-specific or tissue-specific miRNAs are hard to be detected probably because of their low accumulation and/or insufficient stringency of the sequencing approach [7-9]. The advent of new sequencing technologies could make it possible to mine even species-/tissue- specific miRNAs with low abundance, and they havebeen successfully used on *Arabidopsis thaliana*, *Oryza sativa*, *Poplus tricocarpa*, *Medicago tuncatula*, *Gossypiumhirsutum*, *Zea mays*, *Arachis hypogaea* L., *Solanumlycopersicum*, *Citrus trifoliate*, *Vitis vinifera* and *Vitis amurensis* Rupr. [10-21].

Grapevine (*Vitis vinifera* L*.*) is one of the most economically important fruit crops worldwide and has nutritional and processing properties [22]. In recent years, sequencing of small RNA libraries from different grapevine cultivars or tissues has severally been reported where a large number of Vvi-miRNAs were identified [7,18-21]. Despite this, there are no reports on the study of response of Vvi-miRNAs to phytohormones. Phytohormones are important endogenous signals and regulators involved in plant growth and development [23], and they are classified into auxins, gibberellins(GA_3_), cytokinins, abscisic acid(ABA) and ethylene. All of these phytohormones act at low concentrations to regulate different aspects of plant growth and development to varying degrees [23-25]. Among the hormones, GA_3_ play significant regulatoryroles in early berry expansion, berry set and berry ripening [26,27]. Till now, it is still unclear on how GA_3_ participates in the regulation of the complicated developmental processes of grapevine berry.

It has been reported that phytohormone signaling pathways can be effectively controlled by modulation of positive and negative regulators during plant growth and development [4]. Among the modulators of phytohormones, miRNA was recently found to be a new growth regulator involved in phytohormone signaling [28-30], with several studies showing the interactions between miRNAs and phytohormones in various plant responses. For instance, GA_3_ modulates the expression of miR159, while miR159 regulates the development of *Arabidopsi*s anthers and seeds by cleaving the *GAMYB* gene, during *Arabidopsis* anther development [28] and seed germination [29]. In strawberry, miR159 interacts with *GAMYB* during the course of receptacle development, and both of them act in a joint fashion to respond, in part, to changes in endogenous GA_3_ levels [30]. These studies strongly demonstrated that miRNAs are involved in the GA_3_ signaling process.

To account for the roles of miRNAs in response to GA_3_ during grapevine berry development, we constructed two small RNA libraries from mixed tissue samples of grapevine berries sprayed with GA_3_ (treatment) and with water (the control). The grapevine cultivar used for this study is ‘Summer Black’ (hybrid of *V. vinifera*?×?*V. labrusca*), an elite table grapevine cultivar native to Japan. After high-throughput sequencing, we identified a number of conserved and non-conserved Vvi-miRNAs responsive to GA_3_, and we further analyzed their potential role in mediation of GA3-induced regulation of grapevine berry growth and development. Further, qRT-PCR was utilized to analyze the expression of Vvi-miRNAs in different development stages of the grapevine berries subjected to exogenous GA_3_ and in exogenous GA_3_-free berries. Lastly, an attempt to elucidate the regulatory functions of Vvi-miRNAs being responsive to GA_3_ during grapevine berry development was done.

## Results

### Characterization of the Vvi-miRNAs from deep sequencing of grapevine sRNA libraries

To identify GA_3_-responsive miRNAs in grapevine berries, two small RNA libraries from grapevine berries treated with GA_3_ (GA_3_ treatment) and sprayed with water (the control) were constructed. Solexa, a high throughput sequencing technology, was employed to sequence these libraries, leading to a generation of 16,231,320 and 16,486,660 clean reads from GA_3_ treatment and the control libraries, respectively. All these clean reads were those from removal of adaptor, insert, polyA, and RNAs shorter than 18nt in length (Table 1). About 4,265,160 (GA_3_ treatment) and 4,326,915 (the control) clear reads could be mapped to the grapevine genome published in 2007 [31], and miRNA, tRNA, siRNA, snRNA, snoRNA, rRNA, repeat regions, exon and intron RNA reads were annotated. In addition, 9,075,238 and 9,207,588 un-annotated reads were used for prediction of new Vvi-miRNAs in GA_3_ treatment and the control grapevine berries, respectively (Table 1).

**Table 1:** **Distribution of small RNAs among different categories in control and GA**_**3**_**Treated grapevine berries**

**Category**	**Control**				**GA3 treatment**			
	**Unique**	**Percent (%)**	**Redundant**	**Percent (%)**	**Unique**	**Percent (%)**	**Redundant**	**Percent (%)**
Exon_antisense	88740	2.28%	232050	1.41%	67193	1.40%	157256	0.98%
Exon_sense	136907	3.52%	449869	2.73%	130285	2.71%	289348	1.80%
Intron_antisense	35335	0.91%	72189	0.44%	32881	0.68%	52624	0.33%
Intron_sense	41391	1.06%	106364	0.65%	39164	0.81%	72984	0.46%
miRNA	1724	0.04%	683061	4.14%	1726	0.04%	812099	5.07%
rRNA	51658	1.33%	1099955	6.67%	72149	1.50%	1108756	6.92%
Repeat	15219	0.39%	27556	0.17%	12601	0.26%	22740	0.14%
snRNA	4408	0.11%	19905	0.12%	4553	0.09%	19873	0.12%
snoRNA	1780	0.05%	5172	0.03%	1895	0.04%	6403	0.04%
tRNA	13078	0.34%	1285951	7.80%	20052	0.42%	1210840	7.55%
unann	3501164	89.97%	9207588	55.85%	4190436	87.17%	9075238	56.60%
Mapping to genome	838267	21.54%	43326915	26.24%	765402	15.92%	4265160	26.60%
Total	3891404	100.00%	16486660	100.00%	4807290	100%	16033016	100%

**Figure 1:**
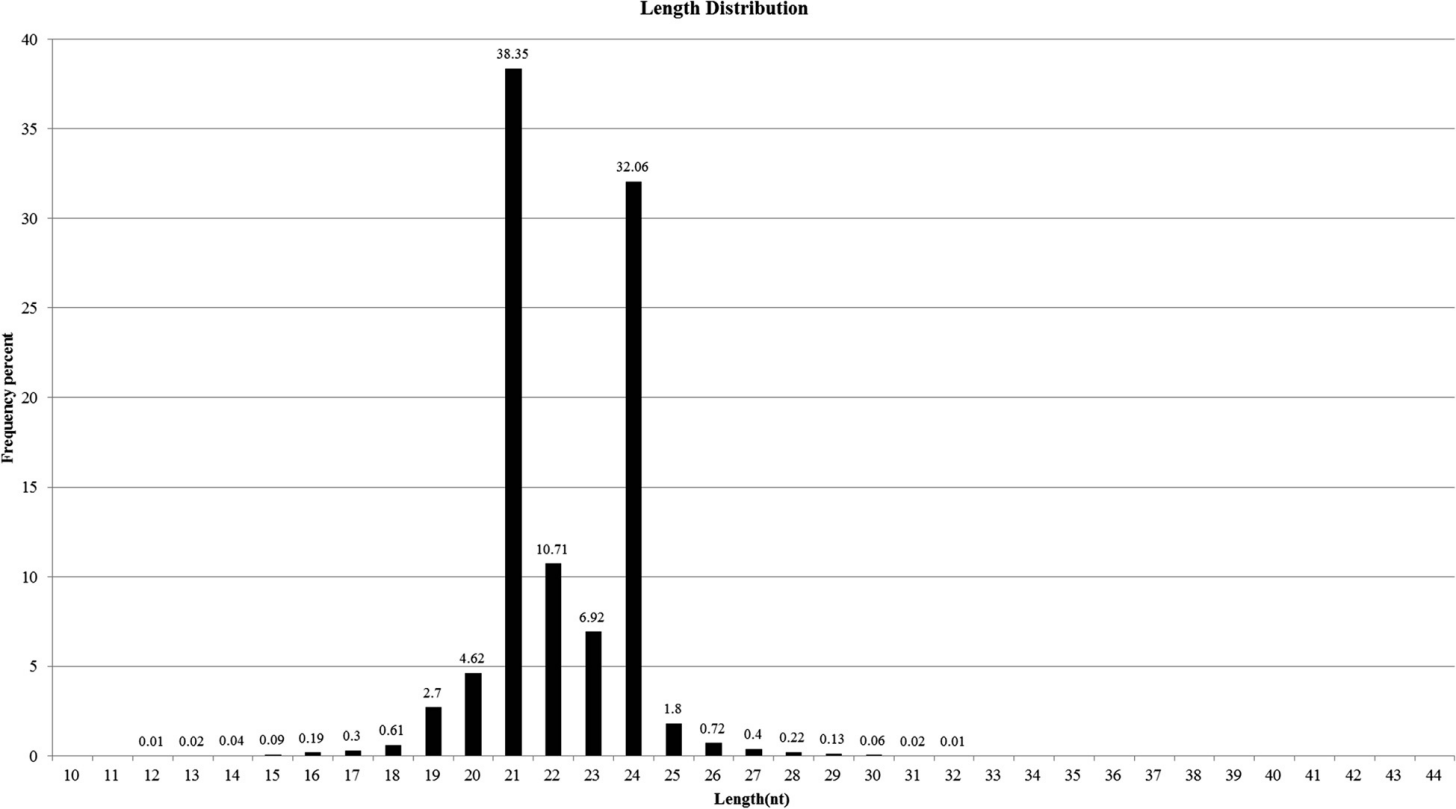
Size distribution of unique small RNA sequences from grapevine.

**Figure 2:**
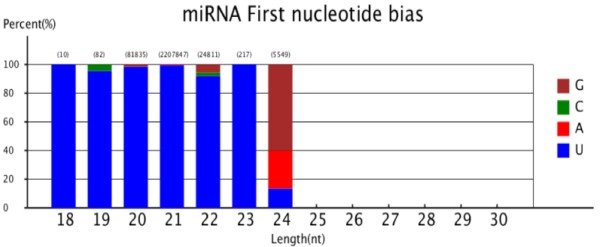
First nucleotide bias of 18-30nt sRNA tags.

The size distribution of all sRNAs was found to be uneven ranging from 18nt to 30nt long, with the majority being 19-25nt long (Figure 1). The sRNAs of 21 nt and 24nt formed two major classes, occupying 38.35% and 32.06% (Figure 1) of the total, respectively, an observation which is in agreement with some previous reports in grapevine and tomato [11,16,21,31], but contrasts to those reported in *Arabidopsis*, rice and peanut [32-35]. The two main peaks of sRNAs in this study were 24 and 21nt long, while the most common sRNAs in *Taxus chinensis*[35] and *Citrus trifoliate*[17] were those with 21 nt in length. These cases suggested that some differences might exist in the sRNA biogenesis pathways in various plants. In addition, analysis of the first nucleotide of 18-25nt long sRNAs indicated that many sRNAs started with a uridine (U) at their 5’-ends and most of them are 21 nt and 22 nt long, with the former being most outstanding in number (Figure 2). Similar to other plants, most miRNAs here were those with 21 and 22 nt in length and they also begin with a 5’uridine, which is one of the important characteristic features of miRNAs [10,11,36].

**Figure 3:**
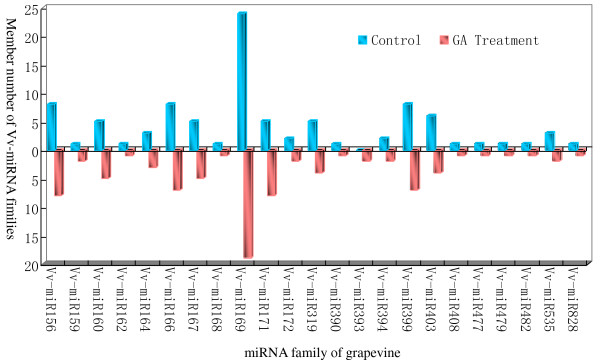
**Number of members from Vv-miRNA families in control and GA**_**3**_**treated grapevine berries.**

High throughput sequencing can verify a large number of known miRNAs together with the identification of novel specific miRNAs even with the low abundance in organisms. From two sRNA libraries in this study, we first searched for known Vvi-miRNAs by comparing our libraries with known miRNAs from other plant species in miRBase 19.0 (http://www.mirbase.org/). A total of 122 known Vvi-miRNAs were sequenced both in the control and GA_3_-treatment libraries, and they belonged to 27 conserved miRNA families according to a comparative genomics-based analysis in different plant species [37]. Eighteen of the 27 Vvi-miRNA families contained many members (Table 2), with four families (Vvi-miR169, Vvi-miR156, Vvi-miR166, Vvi-miR171 and Vvi-miR399) possessing 19, 8, 7, 8, and 7 members, respectively. Another nine Vvi-miRNA families (Vvi-miR162, Vvi-miR168, Vvi-miR390, Vvi-miR397, Vvi-miR408, Vvi-miR477, Vvi-miR479, Vvi-miR482, and Vvi-miR828) had only one member each. Among the known Vvi-miRNAs, the Vvi-miR166 family had the most abundant reads accounting for 63.2% of all the conserved miRNA reads. In this family, the number of Vvi-miR166h reads was over 500,000 in the two libraries, followed by Vvi-miR156, Vvi-miR168, Vvi-miR167, Vvi-miR479, Vvi-miR482 families, whose redundancies were more than several ten thousands. However, other miRNA families like Vvi-miR393, Vvi-miR394, Vvi-miR398 and Vvi-miR399 had only a few or tens of reads. Interestingly, the number and abundance of members of different Vvi-miRNA families between control and GA_3_ libraries were significant divergence (Table 2; Figure 3), which in turn could reflect the discrepancy in their potential functions during the development of grapevine berries responsive to gibberellin.

**Table 2:** **Conserved Vv-miRNAs identified and normalized counts (NC) in control and GA**_**3**_**treated grapevine berries**

**Control**			**GA3treatment**			**Identity**
**miRNA ID**	**Sequences**	**NC**	**miRNA ID**	**Sequences**	**NC**	
Vvi-miR156a	TTGACAGAAGAGAGGGAGCAC	8.81	vv-miR156a	TTGACAGAAGAGAGGGAGCAC	513.79	↑
Vvi-miR156b	AACTGACAGAAGAGAGTGAGCAC	2308.90	vv-miR156b	AACTGACAGAAGAGAGTGAGCAC	0.61	↓
Vvi-miR156c	TGACAGAAGAGAGTGAGCACAC	2214.31	vv-miR156c	TGACAGAAGAGAGTGAGCACAC	0.17	↓
Vvi-miR156d	TGACAGAAGAGAGTGAGCAC	2321.38	vv-miR156d	TGACAGAAGAGAGTGAGCAC	2331.59	●
Vvi-miR156e	TGACAGAGGAGAGTGAGCAC	3.40	vv-miR156e	TGACAGAGGAGAGTGAGCAC	14.83	↑
Vvi-miR156f	CTGTTGACAGAAGATAGAGAGCAC	5215.88	vv-miR156f	CTGTTGACAGAAGATAGAGAGCAC	3.93	↓
Vvi-miR156g	GCTCTCTAGACTTCTGTCATC	5214.31	vv-miR156g	GCTCTCTAGACTTCTGTCATC	0.61	↓
Vvi-miR156i	TTGACAGAAGATAGAGAGCAC	5214.31	vv-miR156i	TTGACAGAAGATAGAGAGCAC	6093.28	↑
		0.00	vv-miR159b	CCTTGGAGTGAAGGGAGCT	0.17	▲
Vvi-miR159c	TTTGGATTGAAGGGAGCTCTA	74.61	vv-miR159c	TTTGGATTGAAGGGAGCTCTA	582.11	↑
Vvi-miR160a	TCCTAGTTGGCATCAGAGGAG	1.66	vv-miR160a	TCCTAGTTGGCATCAGAGGAG	0.09	↓
Vvi-miR160b	GCATGAGGGGAGTCAAGCAGG	1.66	vv-miR160b	GCATGAGGGGAGTCAAGCAGG	4.19	↑
Vvi-miR160c	GCGTGCGAGGAGCCAAGCATA	3.75	vv-miR160c	GCGTGCGAGGAGCCAAGCATA	4.54	●
Vvi-miR160d	TGCCTGGCTCCCTGTATGCCA	4.71			0.00	▼
Vvi-miR160e	GCGTATGAGGAGCCATGCATA	4.71	vv-miR160e	GCGTATGAGGAGCCATGCATA	2.27	↓
		0.00	vv-miR160f	TGCCTGGCTCCCTGTATGCCA	43.98	▲
Vvi-miR162	TCGATAAACCTCTGCATCCAG	236.04	vv-miR162	TCGATAAACCTCTGCATCCAG	231.76	●
Vvi-miR164a	TTGGAGAAGCAGGGCACGTGC	43.02	vv-miR164a	TTGGAGAAGCAGGGCACGTGC	0.17	↓
Vvi-miR164c	TGGAGAAGCAGGGCACGTGCAT	43.19	vv-miR164c	TGGAGAAGCAGGGCACGTGCAT	6.72	↓
Vvi-miR164d	TGGAGAAGCAGGGCACGTGCA	43.02	vv-miR164d	TGGAGAAGCAGGGCACGTGCA	798.08	↑
Vvi-miR166a	TCTCGGACCAGGCTTCATTCCT	762.04	vv-miR166a	TCTCGGACCAGGCTTCATTCCT	7.85	↓
Vvi-miR166b	TCGGACCAGGCTTCATTCCTC	3280.28	vv-miR166b	TCGGACCAGGCTTCATTCCTC	15371.38	↑
Vvi-miR166c	TCGGACCAGGCTTCATTCCCC	21467.36			0.00	▼
Vvi-miR166d	GATTGTTGTCTGGCTCGAGGC	21492.15	vv-miR166d	GATTGTTGTCTGGCTCGAGGC	1.05	↓
Vvi-miR166e	GGAATGTTGTCTGGCTCGAGG	21467.36	vv-miR166e	GGAATGTTGTCTGGCTCGAGG	311.87	↓
Vvi-miR166f	GGAATGTTGGCTGGCTCGAGG	21506.81	vv-miR166f	GGAATGTTGGCTGGCTCGAGG	18.24	↓
Vvi-miR166g	TTCGGACCAGGCTTCATTCCC	21509.60	vv-miR166g	TTCGGACCAGGCTTCATTCCC	82.11	↓
Vvi-miR166h	TCGGACCAGGCTTCATTCCCC	22171.73	vv-miR166h	TCGGACCAGGCTTCATTCCCC	22520.68	●
Vvi-miR167a	TGAAGCTGCCAGCATGATCTGG	32.81	vv-miR167a	TGAAGCTGCCAGCATGATCTGG	11.78	↓
Vvi-miR167b	TGAAGCTGCCAGCATGATCTAAG	616.06	vv-miR167b	TGAAGCTGCCAGCATGATCTAAG	38.48	↓
Vvi-miR167c	TGAAGCTGCCAGCATGATCTC	134.12	vv-miR167c	TGAAGCTGCCAGCATGATCTC	1901.57	↑
Vvi-miR167d	TGAAGCTGCCAGCATGATCTAG	424.87	vv-miR167d	TGAAGCTGCCAGCATGATCTAG	1.75	↓
Vvi-miR167e	TGAAGCTGCCAGCATGATCTA	613.09	vv-miR167e	TGAAGCTGCCAGCATGATCTA	2349.30	↑
Vvi-miR168	TCGCTTGGTGCAGGTCGGGAA	2963.35	vv-miR168	TCGCTTGGTGCAGGTCGGGAA	2654.62	●
Vvi-miR169a	CAGCCAAGGATGACTTGCCGG	13.09			0.00	▼
Vvi-miR169b	GGTCGAATTGAGCCAAGGATGG	5.58	vv-miR169b	GGTCGAATTGAGCCAAGGATGG	0.35	↓
Vvi-miR169c	TCCGGCAAGTTGTCCTTGGCTAC	13.09	vv-miR169c	TCCGGCAAGTTGTCCTTGGCTAC	0.70	↓
Vvi-miR169d	CAGCCAAGAATGATTTGCCGG	35.25	vv-miR169d	CAGCCAAGAATGATTTGCCGG	105.50	↑
Vvi-miR169e	TAGCCAAGGATGACTTGCCT	0.44			0.00	▼
Vvi-miR169f	TGGGCAAGTTGTGTTTGGCTAC	0.35	vv-miR169f	TGGGCAAGTTGTGTTTGGCTAC	0.26	●
Vvi-miR169g	CAGCCAAGGATGACTTGCCGA	0.44	vv-miR169g	CAGCCAAGGATGACTTGCCGA	6.54	↑
Vvi-miR169h	TGAGCCAAGGATGGCTTGCCGT	5.50	vv-miR169h	TGAGCCAAGGATGGCTTGCCGT	7.42	●
Vvi-miR169i	CTGGTCATGCACGGCTGGTTA	1.83	vv-miR169i	CTGGTCATGCACGGCTGGTTA	0.09	↓
Vvi-miR169j	CAGCCAAGGATGACTTGCCGG	13.09			0.00	▼
Vvi-miR169k	AGCCAAGGATGACTTGCCGGA	13.09	vv-miR169k	AGCCAAGGATGACTTGCCGGA	0.26	↓
Vvi-miR169l	TGAGCCAAGGATGACTTGCCGT	58.12	vv-miR169l	TGAGCCAAGGATGACTTGCCGT	0.96	↓
Vvi-miR169m	TGAGCCAAGGATGACTTGCCG	54.45			0.00	▼
Vvi-miR169n	AAGCATCTGAGGCTCTATTTC	16.32	vv-miR169n	AAGCATCTGAGGCTCTATTTC	186.82	↑
Vvi-miR169o	TGAGCCAAGGATGACTTGCCG	54.80	vv-miR169o	TGAGCCAAGGATGACTTGCCG	13.53	↓
Vvi-miR169p	GCAAGCATCCGAGGCTCTGT	55.50	vv-miR169p	GCAAGCATCCGAGGCTCTGT	3.66	↓
Vvi-miR169q	TAGAGCCAAGGATGACTTGCCG	16.40	vv-miR169q	TAGAGCCAAGGATGACTTGCCG	6.37	↓
Vvi-miR169r	TGAGTCAAGGATGACTTGCCGA	4.71	vv-miR169r	TGAGTCAAGGATGACTTGCCGA	0.79	↓
Vvi-miR169s	CAGCCAAGGATGACTTGCCGG	13.18			0.00	▼
Vvi-miR169t	GGCAAGTTGACTTGACTCAGT	1.66	vv-miR169t	GGCAAGTTGACTTGACTCAGT	6.81	↑
Vvi-miR169u	GGCAAGTTGACTTGACTCTGT	2.71	vv-miR169u	GGCAAGTTGACTTGACTCTGT	4.01	●
Vvi-miR169v	AAGCCAAGGATGAATTGCCGG	4.36	vv-miR169v	AAGCCAAGGATGAATTGCCGG	2.62	↓
Vvi-miR169w	CAGCCAAGGATGACTTGCCGG	13.18	vv-miR169w	CAGCCAAGGATGACTTGCCGG	12.39	●
Vvi-miR169x	TGAGTCAAGGATGACTTGCCGA	0.52	vv-miR169x	TGAGTCAAGGATGACTTGCCGA	0.70	●
Vvi-miR171a	TGTTGGGACGGCTCAATCAAA	4.71	vv-miR171a	TGTTGGGACGGCTCAATCAAA	5.67	●
Vvi-miR171b	TTGAGCCGCGTCAATATCTCC	4.36	vv-miR171b	TTGAGCCGCGTCAATATCTCC	35.69	↑
Vvi-miR171c	GGATATTGGTGCGGTTCAATA	5.15	vv-miR171c	GGATATTGGTGCGGTTCAATA	4.54	●
Vvi-miR171d	TTGATTGAGCCGTGCCAATAT	5.15	vv-miR171d	TTGATTGAGCCGTGCCAATAT	2.53	↓
		0.00	vv-miR171e	TGATTGAGCCGCGCCAATATC	0.09	▲
		0.00	vv-miR171f	TTGAGCCGCGCCAATATCACT	2.01	▲
		0.00	vv-miR171h	TTGAGCCGCGCCAATATCCCG	0.96	▲
Vvi-miR171i	TGATTGAGCCGTGCCAATATC	4.71	vv-miR171i	TGATTGAGCCGTGCCAATATC	85.43	↑
Vvi-miR172c	GGAGCATCATCAAGATTCACA	0.09	vv-miR172c	GGAGCATCATCAAGATTCACA	103.40	↑
Vvi-miR172d	AGAATCTTGATGATGCTGCAT	184.47	vv-miR172d	AGAATCTTGATGATGCTGCAT	117.63	↓
Vvi-miR319b	TTGGACTGAAGGGAGCTCCC	0.35			0.00	▼
Vvi-miR319c	ATTGAATGATGCGGGAGACAA	0.35	vv-miR319c	ATTGAATGATGCGGGAGACAA	20.68	↑
Vvi-miR319e	TTTGGACTGAAGGGAGCTCCT	18.59	vv-miR319e	TTTGGACTGAAGGGAGCTCCT	7.94	↓
Vvi-miR319f	TGCTTGGACTGAAGGGAGC	0.35	vv-miR319f	TGCTTGGACTGAAGGGAGC	3.40	↑
Vvi-miR319g	TTGGACTGAAGGGAGCTCCC	0.26	vv-miR319g	TTGGACTGAAGGGAGCTCCC	1.40	↑
Vvi-miR390	AAGCTCAGGAGGGATAGCGCC	1.75	vv-miR390	AAGCTCAGGAGGGATAGCGCC	160.73	↑
		0.00	vv-miR393a	ATCATGCTATCCCTTAGGAAC	1.66	▲
		0.00	vv-miR393b	GGAGGAGGCATCCAAAGGGAT	0.79	▲
Vvi-miR394a	TTGGCATTCTGTCCACCTCC	2.71			0.00	▼
Vvi-miR394b	TATTGGCATTCTGTCCACCTCC	2.53	vv-miR394b	TATTGGCATTCTGTCCACCTCC	0.09	↓
Vvi-miR394c	TTGGCATTCTGTCCACCTCC	2.71	vv-miR394c	TTGGCATTCTGTCCACCTCC	0.79	↓
Vvi-miR395a	CTGAAGTGTTTGGGGGAACTC	22.08			0.00	▼
Vvi-miR395b	CTGAAGTGTTTGGGGGAACTC	22.08			0.00	▼
Vvi-miR395c	CTGAAGTGTTTGGGGGAACTC	22.08			0.00	▼
Vvi-miR395d	CTGAAGTGTTTGGGGGAACTC	22.08			0.00	▼
Vvi-miR395e	CTGAAGTGTTTGGGGGAACTC	22.08			0.00	▼
Vvi-miR395f	CACTGAAGTGTTTGGGGGAAC	22.08	vv-miR395f	CACTGAAGTGTTTGGGGGAAC	0.09	↓
Vvi-miR395g	GTTCCCCTGAGCACTTCATTG	22.16	vv-miR395g	GTTCCCCTGAGCACTTCATTG	0.52	↓
Vvi-miR395h	CTGAAGTGTTTGGGGGAACTC	22.08			0.00	▼
Vvi-miR395i	CTGAAGTGTTTGGGGGAACTC	22.08			0.00	▼
Vvi-miR395j	CTGAAGTGTTTGGGGGAACTC	22.08			0.00	▼
Vvi-miR395k	GTTCCCTTGACCACTTCACTG	22.08	vv-miR395k	GTTCCCTTGACCACTTCACTG	0.44	↓
Vvi-miR395l	CCCCTAGAGTTCCCCTGACCA	22.08	vv-miR395l	CCCCTAGAGTTCCCCTGACCA	0.09	↓
Vvi-miR395m	CTGAAGTGTTTGGGGGAACTC	22.08	vv-miR395m	CTGAAGTGTTTGGGGGAACTC	11.61	↓
Vvi-miR396a	CTCAAGAAAGCTGTGGGAGG	25.04	vv-miR396a	CTCAAGAAAGCTGTGGGAGG	35.17	↑
Vvi-miR396b	TTCCACAGCTTTCTTGAACTT	53.23	vv-miR396b	TTCCACAGCTTTCTTGAACTT	35.86	↓
Vvi-miR396c	TTCCACAGCTTTCTTGAACTG	13.53	vv-miR396c	TTCCACAGCTTTCTTGAACTG	18.41	↑
Vvi-miR396d	GTTCAATAAAGCTGTGGGAAG	14.22	vv-miR396d	GTTCAATAAAGCTGTGGGAAG	6.20	↓
Vvi-miR397a	TCATTGAGTGCAGCGTTGATG	7.59	vv-miR397a	TCATTGAGTGCAGCGTTGATG	29.41	↑
Vvi-miR398a	CAAGGGAGTGGCACCTGAGAACA	0.09	vv-miR398a	CAAGGGAGTGGCACCTGAGAACA	6.98	↑
Vvi-miR398b	GGTGTGACCTGAGAATCACATG	0.61	vv-miR398b	GGTGTGACCTGAGAATCACATG	0.26	↓
Vvi-miR398c	TGTGTTCTCAGGTCGCCCCTG	0.61	vv-miR398c	TGTGTTCTCAGGTCGCCCCTG	1.13	↑
Vvi-miR399a	GTGTGATTCTCCTTTGGCAGA	0.70	vv-miR399a	GTGTGATTCTCCTTTGGCAGA	1.57	↑
Vvi-miR399b	TGCCAAAGGAGAGTTGCCCTG	0.26			0.00	▼
Vvi-miR399c	TGCCAAAGGAGAGTTGCCCTG	0.26	vv-miR399c	TGCCAAAGGAGAGTTGCCCTG	0.09	↓
Vvi-miR399d	TCTGCCAAAGGAGATTTGCTC	1.48	vv-miR399d	TCTGCCAAAGGAGATTTGCTC	0.26	↓
Vvi-miR399e	TGCCAAAGGAGATTTGCCCGG	3.58	vv-miR399e	TGCCAAAGGAGATTTGCCCGG	10.56	↑
Vvi-miR399g	TGCCAAAGGAGATTTGCCCCT	9.08	vv-miR399g	TGCCAAAGGAGATTTGCCCCT	1.31	↓
Vvi-miR399h	TGCCAAAGGAGAATTGCCCTG	0.52	vv-miR399h	TGCCAAAGGAGAATTGCCCTG	0.96	↑
Vvi-miR399i	CGCCAAAGGAGAGTTGCCCTG	29.49	vv-miR399i	CGCCAAAGGAGAGTTGCCCTG	14.05	↓
Vvi-miR403a	TTAGATTCACGCACAAACTCG	52.27			0.00	▼
Vvi-miR403b	TTAGATTCACGCACAAACTCG	52.62			0.00	▼
Vvi-miR403c	CGCACAAACTCGTGATCTGTC	52.27	vv-miR403c	CGCACAAACTCGTGATCTGTC	0.09	↓
Vvi-miR403d	AGTTTGTGCGCGAATCCAACC	52.62	vv-miR403d	AGTTTGTGCGCGAATCCAACC	4.36	↓
Vvi-miR403e	TTAGATTCACGCACAAACTCGC	52.18	vv-miR403e	TTAGATTCACGCACAAACTCGC	0.17	↓
Vvi-miR403f	TTAGATTCACGCACAAACTCG	52.36	vv-miR403f	TTAGATTCACGCACAAACTCG	586.04	↑
Vvi-miR408	ACGGGGACGAGGTAGTGCATG	22.34	vv-miR408	ACGGGGACGAGGTAGTGCATG	62.04	↑
Vvi-miR477	TCCCTCAAAGGCTTCCAATTT	98.25	vv-miR477	TCCCTCAAAGGCTTCCAATTT	21.82	↓
Vvi-miR479	TGTGGTATTGGTTCGGCTCATC	2922.60	vv-miR479	TGTGGTATTGGTTCGGCTCATC	1131.41	↓
Vvi-miR482	AATTGGAGAGTAGGAAAGCTT	45.11	vv-miR482	AATTGGAGAGTAGGAAAGCTT	1056.46	↑
Vvi-miR535a	TGACAACGAGAGAGAGCACGC	763.18			0.00	▼
Vvi-miR535b	ACGAGAGAGAGCACGCTAGTCAG	763.18	vv-miR535b	ACGAGAGAGAGCACGCTAGTCAG	0.09	↓
Vvi-miR535c	TGACAACGAGAGAGAGCACGC	763.18	vv-miR535c	TGACAACGAGAGAGAGCACGC	83.42	↓
Vvi-miR828a	AGATGCTCATTTGAGGAAGCAA	1.13	vv-miR828a	AGATGCTCATTTGAGGAAGCAA	5.32	↑

Notes: **↑**,**↓**,▲ ,▼,● denote up-regulated, down-regulated, induced, repressed, un-affected, respectively.

Bioinformatics analysis of the sequencing data could identify novel Vvi-miRNAs based on the criteria of novel miRNA annotations developed by Meyers *et al.* (2008) [38]. The genomic sequences with flanking un-announced sequences were extracted. Among these sequences, 90 candidate Vvi-miRNAs were firstly uncovered, and the hairpin structures of their precursors’ could be predicted (Additional file 1). The miRNA* sequences available is of vital evidence supporting the release of miRNA duplex from the predicted foldback structure [16]. Among these 90 potential novel Vvi-miRNAs, 28 had their Vvi-miRNA* sequences were detected (Additional file 2), and the sequences of 45 novel potential Vvi-miRNAs started with a 5’ uridine, the important feature of miRNAs (Additional file 2). All these confirmed the existence of these novel candidate Vvi-miRNAs in grapevine. In this study, it was also noted that the novel Vvi-miRNAs had fewer reads than the conserved ones. 72% of the former had only several or dozens of copies except for Vvi-miRC20, Vvi-miRC36, Vvi-miRC43, Vvi-miRC44, Vvi-miRC45, Vvi-miRC46, Vvi-miRC50, Vvi-miRC51, Vvi-miRC52, Vvi-miRC71, and Vvi-miRC74 that were sequenced more than one thousand times (Additional file 2). This agreed with other previously reported results [10,11,36], where most novel species-specific miRNAs were usually expressed at lower levels than their conserved counterparts and were much more spatiotemporally expressed.

### Discovery of miRNAs responsive to exogenous GA_3_ in grapevine

To identify Vvi-miRNAs responsive to exogenous GA_3_, we normalized the counts of reads sequenced based on the systematical analysis, and compared the members and the normalized counts (NC) of different members of Vvi-miRNA families between the control and GA_3_ libraries. The results revealed that seven known Vvi-miRNAs (Vvi-miR159b, Vvi-miR160f, Vvi-miR171e, Vvi-miR171f, Vvi-miR171h, Vvi-miR393a and Vvi-miR393b) were found only in the GA_3_ treated grapevine tissues not in the control, suggesting they were probably those induced by GA_3_; another 21 known Vvi-miRNAs (Vvi-miR160d, Vvi-miR166c, Vvi-miR169a, Vvi-miR169e, Vvi-miR169j, Vvi-miR169m, Vvi-miR169s, Vvi-miR319b, Vvi-miR394a, Vvi-miR395a, Vvi-miR395b, Vvi-miR395c, Vvi-miR395d, Vvi-miR395e, Vvi-miR395h, Vvi-miR395i, Vvi-miR395j, Vvi-miR399b, Vvi-miR403a, Vvi-miR403b and Vvi-miR535a) were strongly repressed in GA_3_ treatment for they could only be detected in the control but not in the GA_3_ treatment (Table 2).

Thirty novel Vvi-miRNAs (Vvi-miRC02, Vvi-miRC03, Vvi-miRC05, Vvi-miRC08, Vvi-miRC10, Vvi-miRC11, Vvi-miRC13, Vvi-miRC14, Vvi-miRC16, Vvi-miRC17, Vvi-miRC21-Vvi-miRC26, Vvi-miRC33, Vvi-miRC39, Vvi-miRC41, Vvi-miRC42, Vvi-miRC54, Vvi-miRC55, Vvi-miRC57, Vvi-miRC59, Vvi-miRC61, Vvi-miRC64, Vvi-miRC65, Vvi-miRC68 and Vvi-miRC72) were found to be responsive to exogenous GA_3_, for they could only be detected in GA_3_ treated grapevines (Table 2); while another 16 novel Vvi-miRNAs (from Vvi-miRC75 to Vvi-miRC90) were repressed by exogenous GA3, and found only in control but not in GA_3_ treatment (Table 2). These results demonstrate that these groups of Vvi-miRNAs could be responsive to GA_3_ treatment.

Further comparison of the normalized counts of Vvi-miRNAs identified in GA_3_ treated and the control plants indicated that many Vvi-miRNAs exhibited drastic variations (increases/decreases over several times) in expression frequencies. As shown in Table 2 and Table 3, the expression of 137 Vvi-miRNAs was strongly responsive to exogenous GA_3_, with 58 of them induced by GA_3_, showing conspicuously up-regulated expression under GA_3_ treatment (pointed as ↑ in Table 2 and 3).Conversely, 51 Vvi-miRNAs were repressed by exogenous GA_3_ (pointed as ↓ in Table 2 and 3), leading to drastic reduction in their expression levels in GA_3_-treated grapevines. An interesting revelation was that diverse members of the same Vvi-miRNA family could exhibit conspicuous discrepancy in their responses to exogenous GA_3_. This aspect is best exemplified by Vvi-miR166 family, where Vvi-miR166d/e/f/g had more than 246,000 reads in control samples, but possessed only several dozen or even a little more reads in the GA_3_ treatment samples, while Vvi-miR166b was detected 37,952 times in control and 176,156 times in GA_3_ treatment (Table 2). Vvi-miR166h, however, had no distinct variations between the control and GA_3_ treatment (Table 2). Similar situations were found in other Vvi-miRNA families like Vvi-miR156, Vvi-miR164, Vvi-miR167, Vvi-miR403 and Vvi-miR535 (Table 2), suggesting the Vvi-miRNAs responsive to exogenous GA_3_ application possess multiple aspects and functions during the development of grapevine berries.

**Table 3:** **Comparison of normalized counts (NC) of novel Vv-miRNAs between GA**_**3**_**treated and control grapevine berries**

**miRNA ID**	**Mature sequences**	**NC**		**Identity of frequency**
		**Control**	**GA treatment**	
Vvi-miRC01	CTATGTTATAGGATCTTGGAT	9.16	17.19	↑
Vvi-miRC01*	CCAAGATACTATAACATGGTC	0.00	0.17	▲
Vvi-miRC02	TCCCTTTGGAAGTGCTAAGCG	0.00	1.83	▲
Vvi-miRC03	AGTGGTGGCAAGGATGAGCAA	0.00	0.52	▲
Vvi-miRC04	TTTGGAATGATTTGTTGATGA	4.62	1.48	↓
Vvi-miRC05	AAGATCTCCCATTGCATCTGA	0.00	0.52	▲
Vvi-miRC06	TTTTTTGGTTATGGTTGGCTG	0.61	1.40	↑
Vvi-miRC07	CTCAAGAAAGCTGTGGGAAAA	0.61	1.05	↑
Vvi-miRC07*	TTTCCACATCTTTCTTGAACT	0.09	0.17	●
Vvi-miRC08	AGAAGAACAAGTAGACTGAGC	0.00	0.96	▲
Vvi-miRC09	TTATATAGGCTTTGAGGATGGA	0.00	1.92	▲
Vvi-miRC10	TTTTAAAAAGGTTCGTCATTC	0.00	0.87	▲
Vvi-miRC11	CCGTGACAAGTGGTATCAGAG	0.00	1.13	▲
Vvi-miRC12	TCTGAAGTTTGAAGAGCTGTG	4.97	10.56	↑
Vvi-miRC12*	AGAGCAATCTACGAACAACAGGAA	0.09	0.09	●
Vvi-miRC13	TTGGCTTGGAGATGGATCATT	0.00	10.56	▲
Vvi-miRC14	TTGGCTTGGAGATGGATCATT	0.00	10.56	▲
Vvi-miRC15	TCAATTTGAGAGCTGGAAGAA	0.61	0.70	●
Vvi-miRC16	ATATTGGTAAATGAATGTTCG	0.00	1.40	▲
Vvi-miRC17	AATTTCTTATGTTCATGATTG	0.00	0.79	▲
Vvi-miRC18	AAGAGCAGTTGAACTGAAGCA	0.70	1.57	↑
Vvi-miRC19	TCTGTCGCAGGAGAGATGATGC	1.66	4.19	↑
Vvi-miRC20	GGAATGGGCTGATTGGGATA	2551.57	760.03	↓
Vvi-miRC20*	TTCCCAATGCCGCCCATTCCAA	94.07	208.55	↑
Vvi-miRC21	CCAAGAGGGTGGAGTTCAGAT	0.00	1.48	▲
Vvi-miRC21*	CTGAACTCTCTCCCTCATGGCC	0.00	0.87	▲
Vvi-miRC22	CTAAATTGCTTCGGGTCCTGC	0.00	6.63	▲
Vvi-miRC22*	AGGAGATGAGGTATGTTTACAT	0.00	5.93	▲
Vvi-miRC23	AAACATGAGTCTGGACCTTGA	0.00	0.79	▲
Vvi-miRC24	AAACATGAGTCTGGACCTTGA	0.00	0.79	▲
Vvi-miRC25	TCTGTTTTCACTCTCATTAAG	0.00	1.13	▲
Vvi-miRC25*	TAGTGAGAATGAGTTGGGGAAG	0.00	0.09	▲
Vvi-miRC26	TCGGAGAAGTGTGATGTGTAT	0.00	0.70	▲
Vvi-miRC27	ATACCATGTGGAAAAGAGGAATC	6.72	5.85	●
Vvi-miRC28	ATTGGCAGAATATTCAAGGTTT	0.44	0.87	↑
Vvi-miRC29	TTATTAGGAGGACATTTAGGTAT	2.88	3.49	●
Vvi-miRC30	TGCGGGTGGAAGAGAAGGAAG	5.67	3.49	↓
Vvi-miRC31	TTCCTGCGGTTTCTCGGCGAC	0.96	0.96	●
Vvi-miRC32	TTTTCCTATGATTTCTTGGCA	0.87	0.79	●
Vvi-miRC32*	CTGGGAAAGCGTGGGAAAACA	0.00	0.09	▲
Vvi-miRC33	TTCCTATCGTTCCCGGGATTT	0.00	1.22	▲
Vvi-miRC34	TGACCGGCTCTTATCTCTCATG	1.48	3.93	↑
Vvi-miRC34*	TGAAGATAAAGAGTCTCGTCTGG	0.35	0.09	↓
Vvi-miRC35	GGAATGGATGGCATGGGAACCA	0.52	0.52	●
Vvi-miRC36	TGAGTAGTGGACTATCGCATG	14.57	1728.01	↑
Vvi-miRC36*	TGAGATAAGTCTGCTGCTCCAT	0.61	94.76	↑
Vvi-miRC37	TGGATGCATGTAGCTTGTCAA	16.06	0.87	↓
Vvi-miRC37*	GACAAGTTACATACATCCAAG	1.05	0.17	↓
Vvi-miRC38	TCCTTCGGCGTCGGCAAATCC	1.75	1.22	↓
Vvi-miRC39	AAGGGTTTCTCACAGAGTTTA	0.00	0.79	▲
Vvi-miRC39*	AGCTCTGTTGGACTCTCTTTG	0.00	0.17	▲
Vvi-miRC40	GAGGAGAATGTAGTGGGGTTA	0.52	0.44	●
Vvi-miRC41	CTTTGATCAGATATTGGATTG	0.00	1.40	▲
Vvi-miRC41*	AGCAGAGTTTGATAGAGGGC	0.00	0.09	▲
Vvi-miRC42	AATGACATGAGTTGGAACTAA	0.00	0.87	▲
Vvi-miRC43	GTTGGAAGCCGGTGGGGGACC	4389.70	425.65	↓
Vvi-miRC44	GTTGGAAGCCGGTGGGGGACC	4389.70	425.65	↓
Vvi-miRC45	GTTGGAAGCCGGTGGGGGACC	4389.70	425.65	↓
*Vvi-miRC46*	*GTTGGAAGTCGGTGGGGGAAC*	1990.92	272.69	↓
Vvi-miRC47	GGCGATTGTAAATATGGGTAA	3.75	1.13	↓
Vvi-miRC48	TCTAGATTTGGAAGTAGGTCA	0.70	0.44	●
Vvi-miRC49	GTTGGAAGTCGGTGGGGGACC	1089.01	72.95	↓
Vvi-miRC50	GTTGGAAGCCGGTGGGGGACC	4389.70	425.65	↓
Vvi-miRC51	TGGGCTTGTGGAGAAGAAAGTGA	0.96	0.52	↓
Vvi-miRC52	CATGGGCGGTTTGGTAAGAGG	2805.93	1401.92	↓
Vvi-miRC52*	TCTTACCAACACCTCCCATTCC	140.49	198.43	↑
Vvi-miRC53	GGTATGGGAGGATTGGGGAGA	1305.32	437.43	↓
Vvi-miRC53*	TTCCCAAGACCCCCCATGCCAA	62.48	327.23	↑
Vvi-miRC54	TCATACCTCGATCTTCGGTTTC	0.00	0.70	▲
Vvi-miRC54*	AATCTGAGATCGAGAATGAAA	0.00	0.09	▲
Vvi-miRC55	ATTCGAACTCAAGACTAAGGT	0.00	41.54	▲
Vvi-miRC56	GAAGCTCTTGAGGGGGACTG	378.18	60.38	↓
Vvi-miRC56*	ACTCTCCCTCAAGGGCTTCTG	12.13	1.31	↓
Vvi-miRC57	AGGTGTAGATGCAAGTGCAGA	0.00	1.05	▲
Vvi-miRC58	TTTAATTTACTAGAGATCTCT	1.13	1.40	●
Vvi-miRC59	GGAGTGAAATTGCAGTGACGG	0.00	1.13	▲
Vvi-miRC60	TCAGCAGGAATTGGACCAGAA	2.88	3.75	●
Vvi-miRC61	ACAGTAGGAAATTGAAAGAGA	0.00	0.70	▲
Vvi-miRC61*	TCTTTCATTTTCCTACTTTTT	0.00	0.52	▲
Vvi-miRC62	AAAGGCGAAGAAAAAGAAGATA	1.75	0.79	↓
Vvi-miRC63	AATATGGAGGACTGTGTTCTT	0.61	1.75	↑
Vvi-miRC63*	GAACTCAGTTCCGGTACCATCTTCA	0.09	0.09	●
Vvi-miRC64	TTGGATTCGCGCACAAACTCG	0.00	1.13	▲
Vvi-miRC65	TTGGATTCGCGCACAAACTCG	0.00	1.13	▲
Vvi-miRC66	CAGCAGTTGCTATTGTGGTTG	0.87	8.38	↑
Vvi-miRC67	AGAAGAGAGAGAGTACAGCTA	1.31	9.60	↑
Vvi-miRC68	TGGTACCAGGAGGGCAACTGTC	0.00	1.05	▲
Vvi-miRC68*	TGTTGCCCTCCTGGTACCATC	0.00	0.09	▲
Vvi-miRC69	TCAAGGGTCGAACGGCTTTGC	1.31	2.36	↑
Vvi-miRC70	TTATGTGAGTGTTCGGCAAATC	0.79	2.62	↑
Vvi-miRC71	TTAGATGATCATCAACAAACA	436.74	517.54	●
Vvi-miRC71*	TTTTGTTGCTGGTCATCTAGTC	2.09	3.05	●
Vvi-miRC72	TGCTTATTAGGTCTGCTGGCA	0.00	0.61	▲
Vvi-miRC73	TCAAAAGAGAAAATGTGGATG	0.52	0.79	●
Vvi-miRC73*	TCCATCTTCTCTCTTTTTACA	0.00	0.09	▲
Vvi-miRC74	TCGCAGGAGAGATGACGCCGT	52.97	110.21	↑
Vvi-miRC74*	AGCATCATTTCTCCTGCATAG	1.13	4.28	↑
Vvi-miRC75	ATATTAGCAGCTGAGAACACA	1.40	0.00	▼
Vvi-miRC76	CAGGACTGGCAGTGATGGTTA	1.13	0.00	▼
Vvi-miRC77	GTGTTTTGCAGGATCAGACGG	0.70	0.00	▼
Vvi-miRC78	TGGCTGAGAACTTGATGGTTA	2.71	0.00	▼
Vvi-miRC79	TTCAAGTCAAAGTCGAACAAG	0.87	0.00	▼
Vvi-miRC80	AGCGAAGTAGTTGTAGGGCTT	0.96	0.00	▼
Vvi-miRC81	TTCGGAGGGAACTGACCGGTT	0.70	0.00	▼
Vvi-miRC82	TGCCAAGAAGCACATTCCTCC	16.84	0.00	▼
Vvi-miRC82*	AGGAATGTGCTTCTTGGCATA	0.09	0.00	▼
Vvi-miRC83	CAAGTGTGGGATTTTGGGTGGCT	0.52	0.00	▼
Vvi-miRC84	GCAGCATCATGAAGATTCACA	0.52	0.00	▼
Vvi-miRC84*	GGAATCTTGATGATGCTGCAT	0.17	0.00	▼
Vvi-miRC85	AGGTGCAGGTGAAGGTGCAGA	1.75	0.00	▼
Vvi-miRC85*	TGCATTTGCACCTGCACCTTA	0.96	0.00	▼
Vvi-miRC86	GTAGCATCATCAAGATTCACA	1.66	0.00	▼
Vvi-miRC87	GGAATGTTGTCTGGCTCGAGGT	0.70	0.00	▼
Vvi-miRC88	ATGTATTTGAGGGAAAGCAAA	0.44	0.00	▼
Vvi-miRC88*	TGTTTTCCCTCAAAAACATGT	0.09	0.00	▼
Vvi-miRC89	CTGCGGGTGGAAAAGGATTAGGC	6.81	0.00	▼
Vvi-miRC89*	CTCATCCTTTTCCATCGGCAGCA	0.35	0.00	▼
Vvi-miRC90	TCTCAGCAACCAAGTAGAGCC	5.93	0.00	▼

Notes: **↑**,**↓**,▲ ,▼,● denote up-regulated, down-regulated, induced, repressed, un-affected, respectively; the symbol * denotes the complementary strands of miRNAs.

### Expression patterns of Vvi-miRNAs responsive to exogenous GA_3_ during grapevine berry development

**Figure 4:**
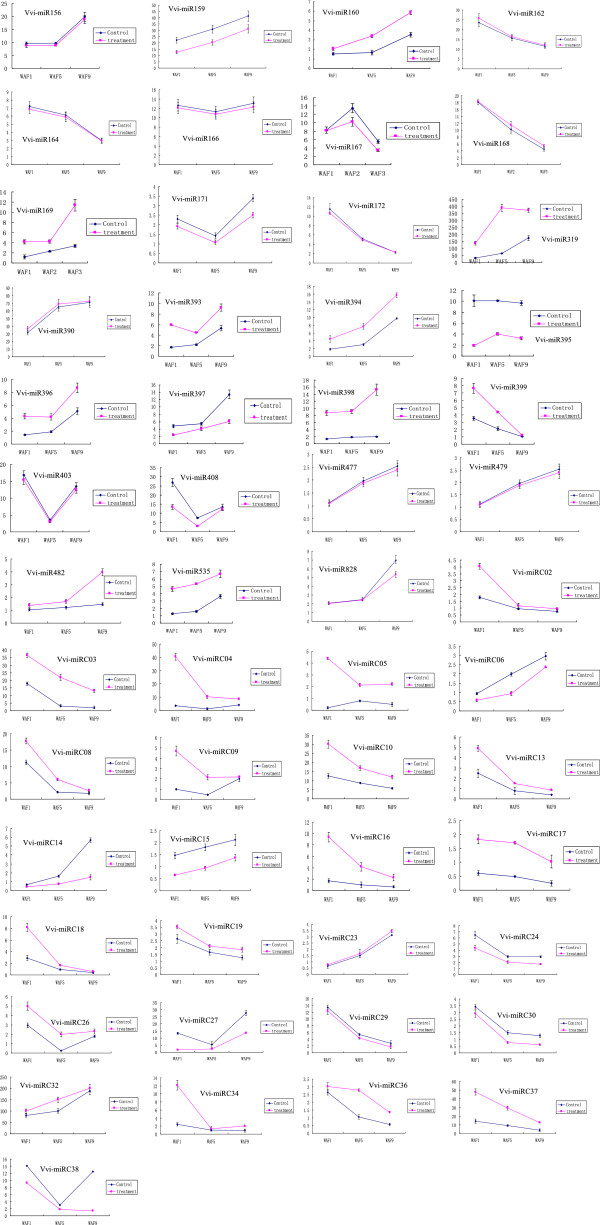
**Expression patterns of miRNAs in grapevine berries underdifferent GA**_**3**_**treatments and control.**

Spatiotemporal expression of grapevine miRNAs could not only provide clues to their physiological functions, but also give fundamental evidence supporting the existence of the miRNAs in grapevine. In this study, 53 Vvi-miRNAs (27 conserved Vvi-miRNAs and 26 novel candidate Vvi-miRNAs) detected in grapevine berries treated with GA_3_ were subjected to qRT-PCR expression analysis as described in Section 2. This could also be applied to the analysis of the degree of response of these Vvi-miRNAs to GA_3_ treatments. We screened for the expression profiles of miRNAs responsive to GA_3_ treatments in the diverse stages of grapevine berries by qRT-PCR. The results showed that the expression levels of 15 Vvi-miRNAs (Vvi-miR169d, Vvi-miR319c, Vvi-miR393a, Vvi-miR396a, Vvi-miR398a, Vvi-miR399a, Vvi-miRC03, Vvi-miRC04, Vvi-miRC05, Vvi-miRC08, Vvi-miRC10, Vvi-miRC13, Vvi-miRC19, Vvi-miRC26 and Vvi-miRC37) were up-regulated by GA_3_, while 13 (Vvi-miR167a, Vvi-miR171d, Vvi-miR395f, Vvi-miR397a, Vvi-miR408, Vvi-miR482, Vvi-miRC06, Vvi-miRC14, Vvi-miRC15, Vvi-miRC24, Vvi-miRC27, Vvi-miRC30, Vvi-miRC38) were down-regulated by GA_3_, and seven (Vvi-miR156d, Vvi-miR166h, Vvi-miR390, Vvi-miR477, Vvi-miRC23, Vvi-miRC29, and Vvi-miRC36) were un-affected by GA_3_ (see Figure 4).

Further analysis of the expression results of 16 known Vvi-miRNAs responsive to GA_3_ both from qRT-PCR and high throughput sequencing revealed that the qRT-PCR expressions of 11 were consistent with the results from high throughput sequencing, which could not only demonstrated the reliability of these two technologies, but confirm the characteristics of these known GA_3_ responsive Vvi-miRNAs. On the other hand, it was discernible that the expression analysis results of 18 novel GA_3_ responsive Vvi-miRNAs from both qRT-PCR and high throughput sequencing were not of the same phenomenon, for only three (Vvi-miRC19, Vvi-miRC14 and Vvi-miRC29) was in the same way. This discrepancy in behavior of conserved and novel Vvi-miRNAs requires further research; while the consistency of the expression results, for the most conserved and a few novel Vvi-miRNAs, from the assays of the qRT-PCR and high throughput may provide some evidence supporting the fact about these Vvi-miRNAs responsive to GA_3_.

### Characterization of potential target genes for novel Vvi-miRNAs responsive to GA_3_

**Figure 5:**
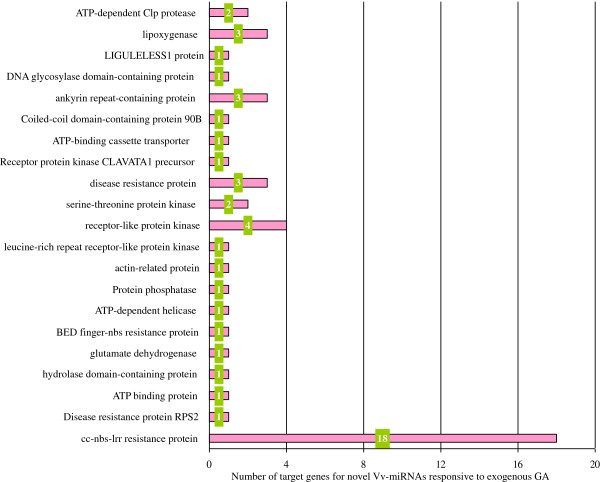
**Functional classification of target genes for novel Vv-miRNAs responsive to exogenous GA**_**3**_**.**

To further comprehend the functions of these newly identified species-specific Vvi-miRNAs, it was essential to search for their target genes. Following the rules suggested by Schwab *et al.* (2005) [39], we searched the grapevine transcript database (http://www.genoscope.cns.fr/spip), and predicted a total of 117 putative target genes for 29 novel Vvi-miRNAs (Additional file 3). Among the 29 novel Vvi-miRNAs, 11 had multiple target genes, as exemplified by Vvi-miRC03 with 19 target genes, indicating these Vvi-miRNAs might possess comprehensive functions in grapevine. Based on orthologous functional annotation in other plants, these target genes seemed to be functionally involved in glucose metabolism (DNA glycosylase domain-containing protein), aromatic substance biosynthesis in berry (lipoxygenase, nigralipoxygenase), signal transduction (receptor-like protein kinase, serine-threonine protein kinase, protein phosphatase), stress resistance (ankyrin repeat-containing protein, disease resistance protein,cc-nbs-lrrresistance protein, domain-containing disease resistance protein, leucine-rich repeat receptor-like protein kinase), etc. (Figure 5; Additional file 3). We also noted that there were a large number of potential target genes with unknown functions for novel Vvi-miRNAs, especially those responsive to GA_3_ (Additional file 3). More in depth investigation on their functions would be essential for thorough understanding of the mechanisms of grapevine flower and fruit development and of the formation of berry quality.

### Verification of potential Vvi-miRNA target genes using 5’-RLM-RACE

**Figure 6:**
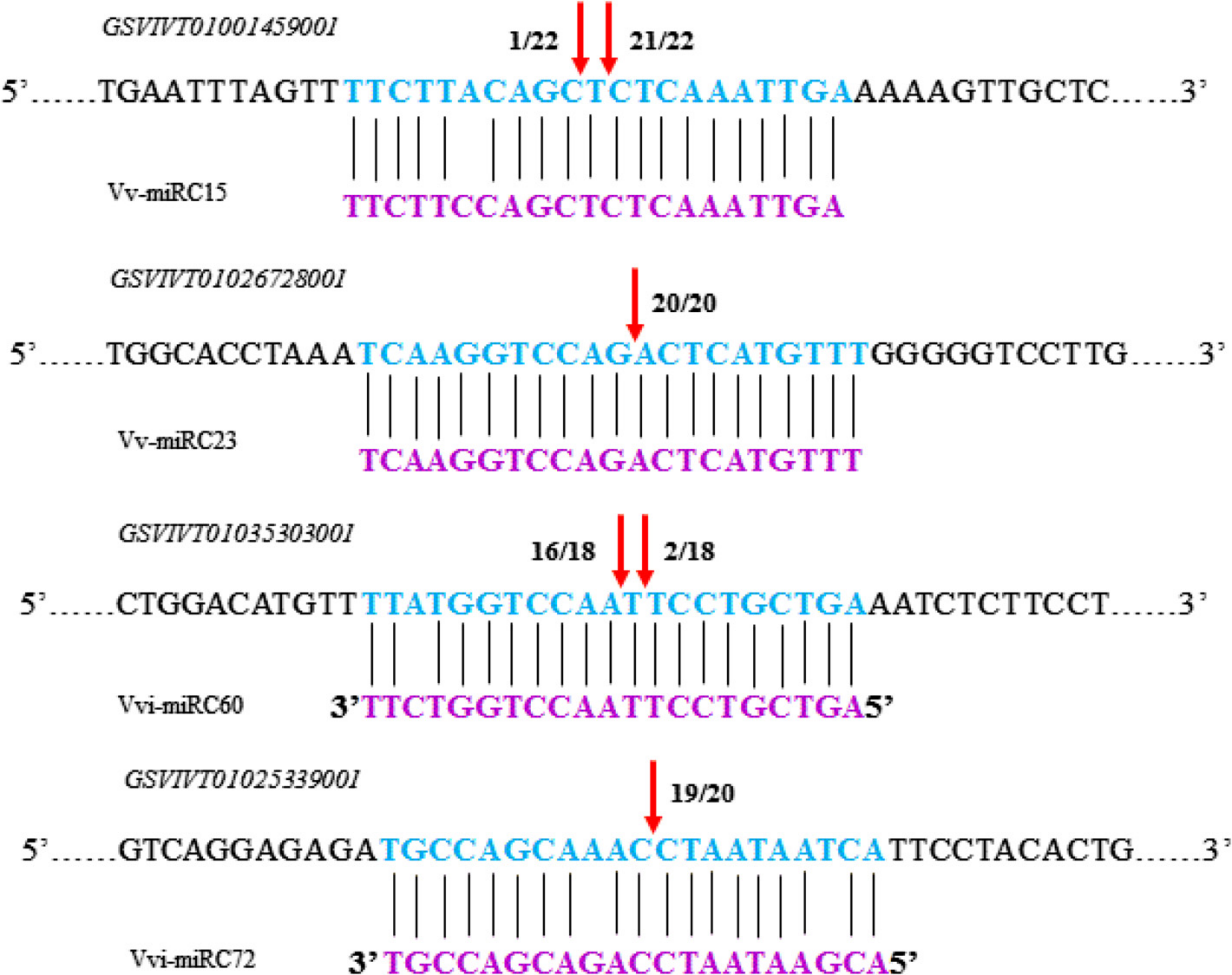
Verification of target genes for Vvi-miRNAs by RLM-5’-RACE.

To verify the nature of potential miRNA targets and to study the Vvi-miRNAs’ regulation on their target genes, a modified RLM-RACE experiment was set up and used to map the cleavage sites in four predicted Vvi-miRNA target genes. Results showed that the cleavage sites of these four miRNA target genes *GSVIVT01000639001, GSVIVT01026728001, GSVIVT01037667001* and *GSVIVT01037667001* for Vvi-miRC15, Vvi-miRC23, Vvi-miRC60 and Vvi-miRC72 in this study is at the nucleotide that pairs with the 9^th^ and/or 10^th^ and/or 11^th^ nucleotide of the corresponding miRNAs (Figure 6), consistent with previous related reports [7,16,18,19,40]. The *GSVIVT01000639001, GSVIVT01026728001, GSVIVT01037667001* and *GSVIVT01037667001* were confirmed as the true targets of Vvi-miR015, Vvi-miR023, Vvi-miR060 andVvi-miR072, respectively. Functional analysis indicated that *GSVIVT01000639001, GSVIVT01026728001, GSVIVT01037667001* and *GSVIVT01037667001* could be of cc-nbs-lrr resistance protein, nbs-lrrresistance protein, leucine-rich repeat family protein, and lipoxygenase (Additional file 3).

## Discussion

To verify the hypothesis that miRNAs play some role inregulating plant response to GA_3_[28], two small RNA libraries from grapevine berries treated with GA_3_ and without GA_3_ were constructed and used for high-throughput sequencing of sRNAs, from which a total of 212 Vvi-miRNAs (both known and novel), with 137 of them being firstly found to be responsive to GA_3_ in grapevine. Further comparison of our dataset from this work to Vvi-miRNAs reported earlier by us [20] revealed that since the grapevine materials used in these studies was the same grapevine cultivar, most of the Vvi-miRNAs could be observed in these two studies, while only a few members (such as Vvi-miR171f, Vvi-miR171h) could be discovered in one of both studies, which may be derived from the differences in the development stages of grapevine materials used in these studies or GA_3_ induced/depressed Vvi-miRNAs in this work.

**Figure 7:**
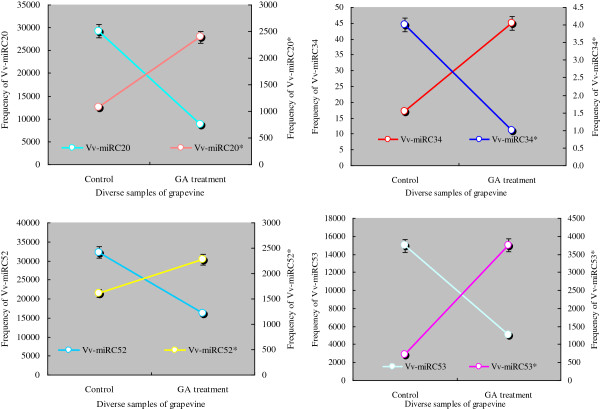
Comparison of expression modes for Vv-miRNAs and Vv-miRNAs*.

The breakthroughs in sequencing technology have provided the most powerful tool for miRNA discovery, and one of the advantages of the thorough approach is its ability to reveal novel miRNAs. In this work, a large number of novel candidate Vvi-miRNAs were uncovered, with the miRNAs* for some novel Vvi-miRNAs being detected too. The identification of miRNAs*of the candidate miRNAs provides convincing evidence for consideration of these Vvi-miRNAs as authentic [38]. Importantly, the expression levels of some novel Vvi-miRNAs and their miRNAs* under GA_3_ treatments had much more diverse variation compared to the control. The best examples of this phenomenon are Vvi-miRC20/Vvi-miRC20*, Vvi-miRC34/Vvi-miRC34*, Vvi-miRC52/Vvi-miR C52* and Vvi-miRC53/Vvi-miRC53* (Figure 7). The expressions of Vvi-miRC20 and Vvi-miRC34 were up-regulated in GA_3_ treatments compared to the control, while the expression of their miRNAs* was down-regulated. On the contrary, the expressions of Vvi-miRC52 and Vvi-miRC53 were down-regulated in GA_3_ treatments, while those of their miRNAs* were up-regulated (Figure 7). The reasons for these discrepancies have not been clearly elucidated. The drastic variation in expression levels of the Vvi-miRNA* under GA_3_ treatments could indicate these miRNAs* were important regulatory genes like Vvi-miRNAs [41].

From high throughput sequencing and qRT-PCR analysis, it was further revealed that the conserve Vvi-miRNAs responsive to GA_3_ had the higher consistency between these two assays, while the novel ones showed some distinct discrepancy. This might be derived from the fact that the conserved Vvi-miRNAs possessed the higher conservation of development and function, while the novel ones had high specificity. Other, the samples used in the high throughput sequencing were the mixed materials from grapevine berries of several development stages, while those used for the qRT-PCR were of three stages berries (1 week after flowering (WAF), 5 WAF, 9 WAF). These could be the reasons explaining the expression levels of novel Vvi-miRNAs from qRT-PCR and high throughput sequencing had more apparent differences than those of conserved Vvi-miRNAs. In addition, the predication of potential target genes for novel Vvi-miRNAs responsive to exogenous GA_3_ and functional annotations of their orthologous target genes in other plants revealed that 20 genes were related to stress resistance. Whether or how these GA_3_ responsive novel Vvi-miRNAs are involved in the regulation of stress resistance can call for further research. The functions of one-third of the novel Vvi-miRNAs were unknown, indicating that more studies need to be performed on these novel miRNAs to elucidate their functions in the growth of grapevine.

## Conclusions

Deep sequencing of short RNAs from grapevine berries in GA3 treatment and the control identified 137 GA_3_-responsive miRNAs, of which 28 exhibited different expression profiles of response to GA_3_ in the diverse developmental stages of grapevine berries. These Vvi-miRNAs identified might be involved in the grapevine berry development and response to various environments.

## Methods

### Plant material

Mixed samples of young berries (one week after flowering, WAF1) large berries (five week after flowering, WAF5), and old berries(nine week after flowering, WAF9) treated with 50 mg/lGA_3_, were collected in 2011 from four-year old ‘Summer Black’ grapevine (hybrids of *V. vinifera* and *V. labrusca*) trees grown under common field conditions at the Nanjing Agricultural University fruit farm, Nanjing, China. Each type of samples had three replicates during deep sequencing and qRT-PCR. All the samples were immediately frozen in liquid nitrogen and stored at −80°C until use.

### Small RNA library construction and sequencing

Mixed Summer Black’ grapevine young berries (one week after flowering), large berries (five week after flowering after flowering), and old berries (nine week after flowering) treated with GA3, wereusedforRNAextraction. The total RNA samples were first extracted usingour modified CTAB method [20]. Isolation of small RNAs and preparation of small RNA libraries were performed based on the procedure of Wang *et al.* (2011) [20]. sRNAs were first separated from the total RNA by size fractionation with 15% TBE urea polyacrylamide gel (TBU) and small RNA regions correspondingto the 18–30 nucleotide bands in the marker lane were excised and recovered. The 18–30 nt small RNAs were 5’ and 3’ RNA adapter-ligated by T4 RNA ligase and at each step, length validated and purified by TBU electrophoretic separation. The adapter-ligated smallRNA was subsequently transcribed into cDNA by SuperScript II Reverse Transcriptase (Invitrogen) and PCR amplified using primers that annealed to the ends of the adapters. The amplified cDNA constructs were purified and recovered. 18 ng cDNA was loaded into the Illumina 1 G Genome Analyzer for sequencing.

### Bioinformatics analysis and identification of Vvi-miRNA

To identify conserved and potential Vvi-miRNAs in grapevine, the raw sequences were processed as described by Sunkar*et al.* (2005) [8]. All sRNAs sequences from 18nt to 30nt obtained were removed from the vector sequences, then the modified sequences were further subjected to removal of rRNA, tRNA, snRNA, snoRNA and those containing the polyA tails, and finally the remaining sequences were compared against known plant Vvi-miRNAs in the miRBase [33]. Only the high matching (0-3mismatches) sequences were considered as conserved Vvi-miRNAs. To study potential Vvi-miRNAs precursor sequences, all sRNAs from grapevine were aligned against the grapevine genome and then the miRNA candidates were processed by miRCat (http://srna-tools.cmp.uea.ac.uk/) [33], using default parameters, to generate the secondary structures (Additional file 1).

### qRT-PCR validation of miRNA expression

The template for RT-PCR was the miRNA-enriched library mentioned above. To amplify the Vvi-miRNAs from the reverse transcribed cDNAs, we used the Vvi-miRNA precise sequences as the forward primers and the mirRacer 3’Primer as the reverse primer [9] (Additional file 3). RT-PCR was conducted with the Rotor-Gene 3000 (Corbett Robotics, Australia) and the Rotor-Gene software version 6.1. For each reaction, 1 μL of diluted cDNA (equivalent to about 100 pg of total RNA) was mixed with 10 μL of 2X SYBR green reaction mix (SYBR® Green qRT-PCR Master Mix; Toyobo, Osaka, Japan), and 5 pmol each of the forward and the reverse primers were added in a final volume of 20 μL. The conditions for the PCR amplification were as follows: polymerase activation at 95°C for 1 min, then 95°C for 1 min, followed by 50 cycles of 95°C for 15 s, 95°C for 15 s, 60°C for 20 s, and 72°C for 20 s. The comparative quantification procedure was used to determine relative expression levels, and the homologous genes of the *Arabidopsis 5.8S rRNA* in grapevine berries was previously used as a reference gene in the qPCR detection of miRNAs in *Arabidopsis*[41]. The data were analyzed with an R^2^ above 0.998 using the LinRegPCR program [42].

### Prediction of potential target mRNAs for Vvi-miRNAs

Target predictions were performed based on methods described by Schwab *et al.*(2005) [39]. Putative Vvi-miRNAs were first blasted against the grapevine unigene database on the Genoscope (http://www.genoscope.cns.fr/). BLASTn hits possessing less than four mismatches were chosen as the candidate targets, and then BLASTx was used to obtain their putative functions. The sequences of predicted targets and their functions are shown in Additional file 4.

### Data access

The sRNA sequence data from this study have been submitted to Gene Expression Omnibus (GEO) under accession No. at website: http://www.ncbi.nlm.nih.gov/geo/query/acc.cgi?token=dlihnquim uscezm&acc=GSE3973.

## Competing interests

The authors declare that they have no competing interests.

## Authors’ contributions

JH and CW carried out the molecular genetic studies, participated in the sequence alignment. YY and XS carried out the RT-PCR arrays. XL and CS participated in the sequence alignment. CW performed the statistical analysis. JH and CW drafted the manuscript. JF and CW conceived of the study, and participated in its design and coordination. JF revised the manuscript. All authors read and approved the final manuscript.

## Supplementary Material

Additional file 1 Secondary structures of the identified novel Vv-miRNAs. Click here for file

Additional file 2 **Novel Vv-miRNAs identified in GA**_3_**treated and the control grapevine berries.** Click here for file

Additional file 3 List of predicted target genes of novel miRNAs identified in grapevines. Click here for file

Additional file 4 Primer sequences of qRT-PCR validated novel miRNAs. Click here for file
