# Supplementary material for: Grapevine microRNAs responsive to exogenous gibberellin
Source: BMC Genomics. 2014 Feb 8;15:111. doi: 10.1186/1471-2164-15-111 (PMC3937062; doi:10.1186/1471-2164-15-111)

Figure S1　Second structures of the identified novel Vv-miRNAs

grape-m0001

grape-m0001-3p：CCAAGATACTATAACATGGTC

grape-m0001-5p：CTATGTTATAGGATCTTGGAT

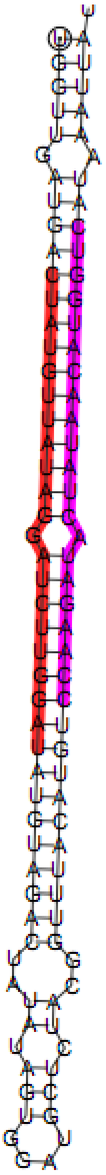

grape-m0002

grape-m0002-3p：TCCCTTTGGAAGTGCTAAGCG

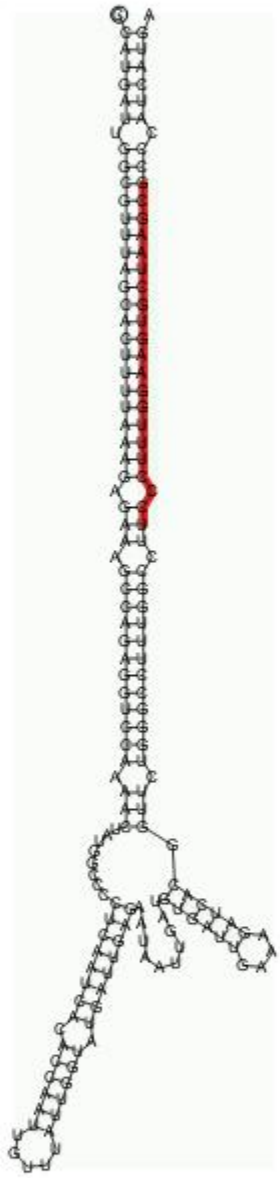

grape-m0003

grape-m0003-3p：AGTGGTGGCAAGGATGAGCAA

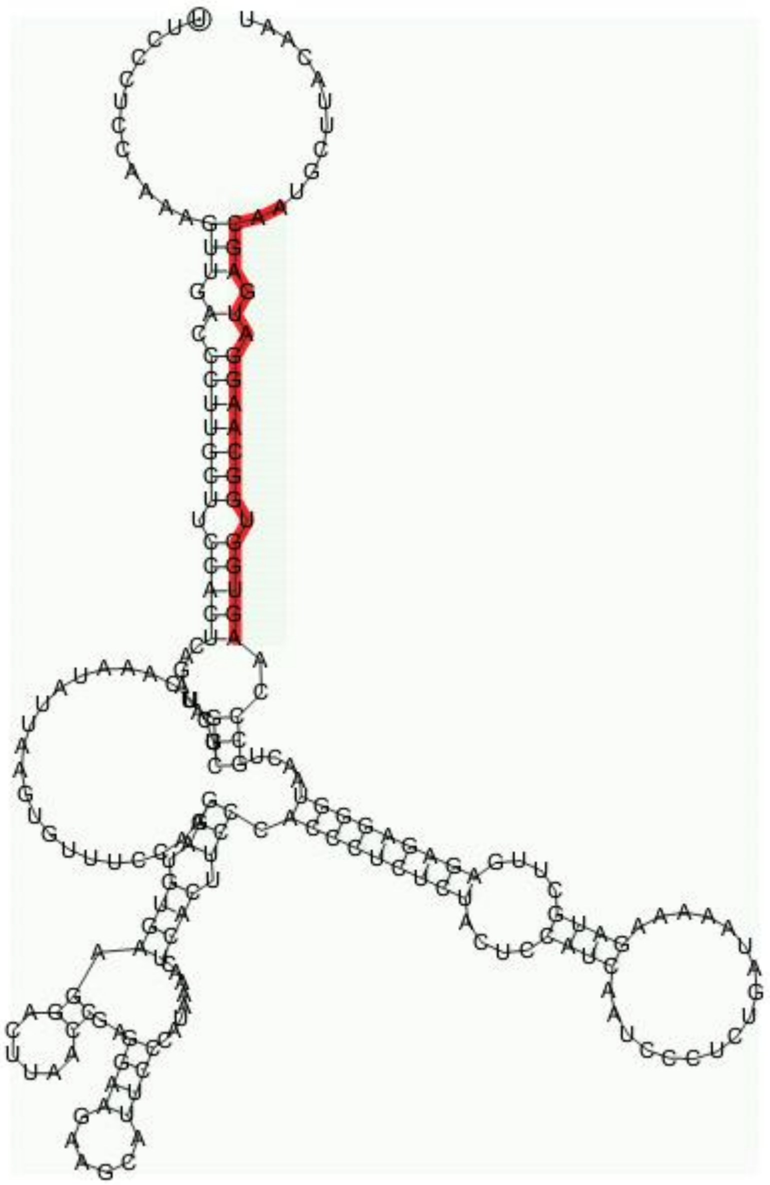

grape-m0004

grape-m0004-5p：TTTGGAATGATTTGTTGATGA

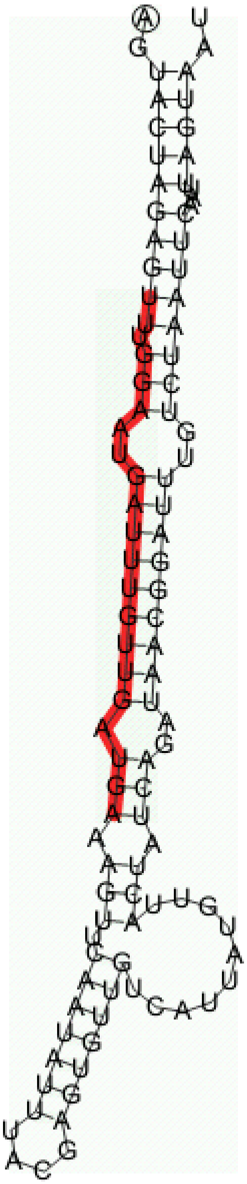

grape-m0005

grape-m0005-3p：AAGATCTCCCATTGCATCTGA

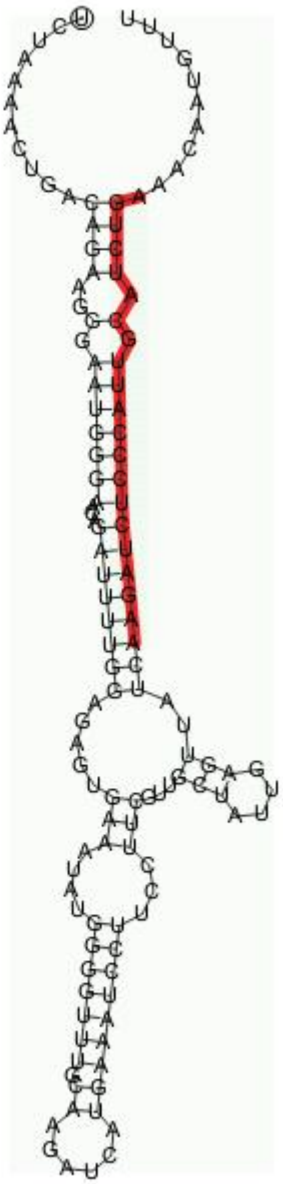

grape-m0006

grape-m0006-3p：TTTTTTGGTTATGGTTGGCTG

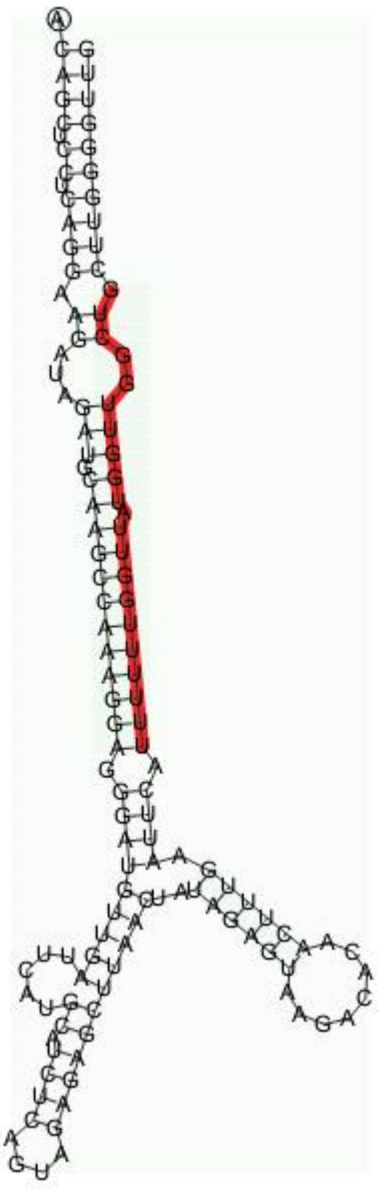

grape-m0007

grape-m0007-5p:TTTCCACATCTTTCTTGAACT

grape-m0007-3p:CTCAAGAAAGCTGTGGGAAAA

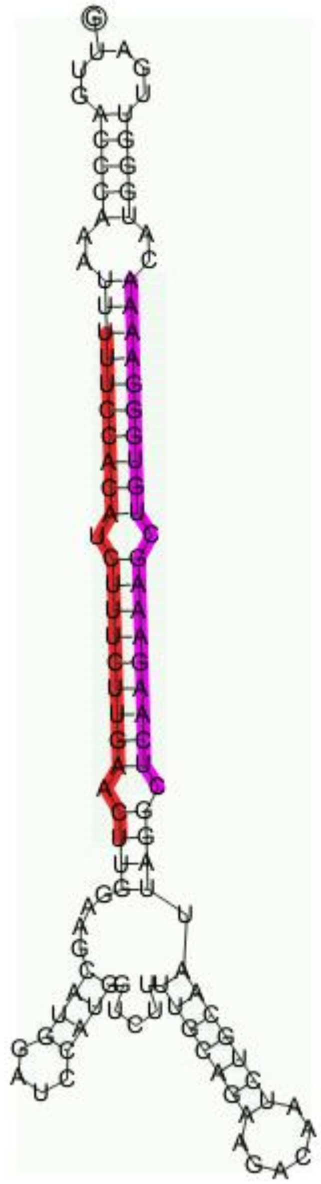

grape-m0008

grape-m0008-3p:AGAAGAACAAGTAGACTGAGC

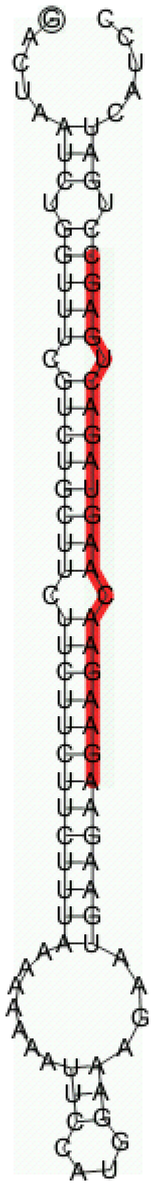

grape-m0009

grape-m0009-3p:TTATATAGGCTTTGAGGATGGA

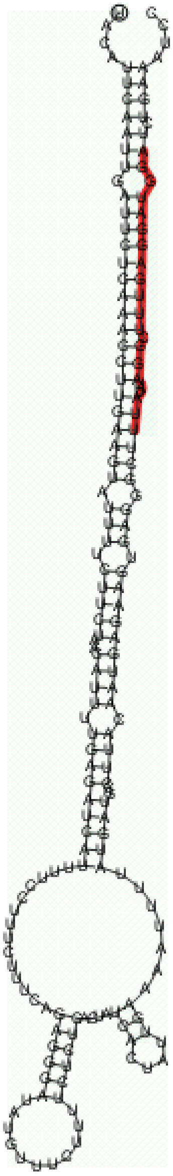

grape-m0010

grape-m0010-5p:TTTTAAAAAGGTTCGTCATTC

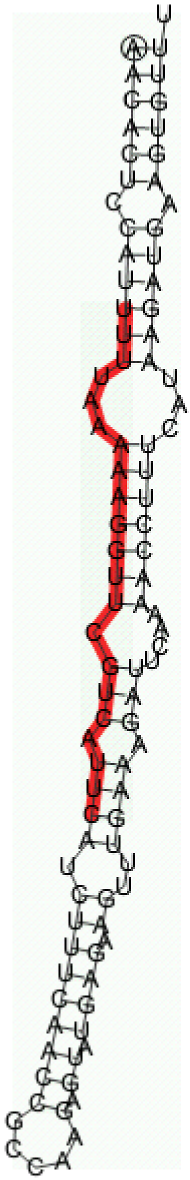

grape-m0011

grape-m0011-3p:CCGTGACAAGTGGTATCAGAG

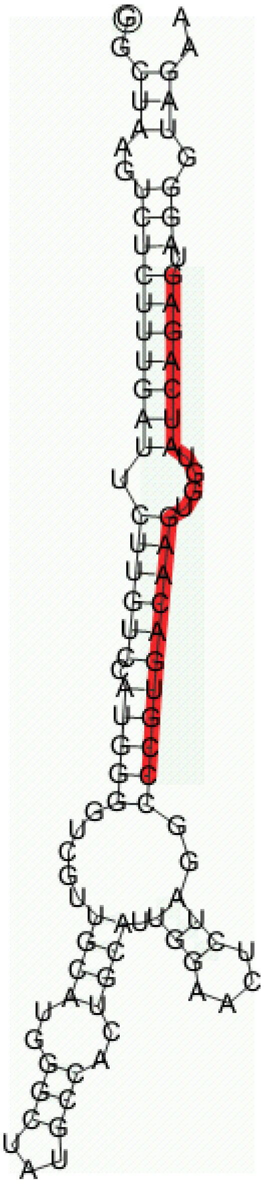

grape-m0012

grape-m0012-5p:TCTGAAGTTTGAAGAGCTGTG

grape-m0012-3p:AGAGCAATCTACGAACAACAGGAA

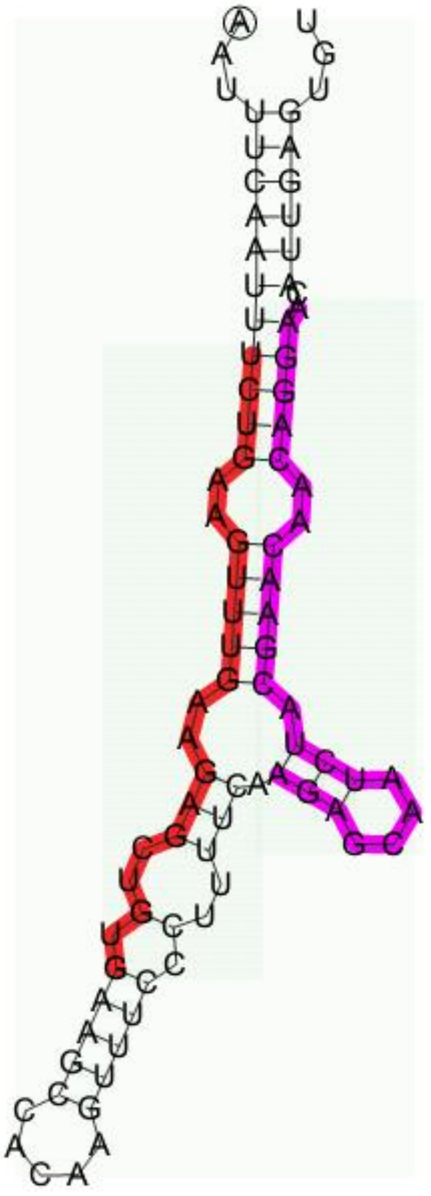

grape-m0013

grape-m0013-3p:TTGGCTTGGAGATGGATCATT

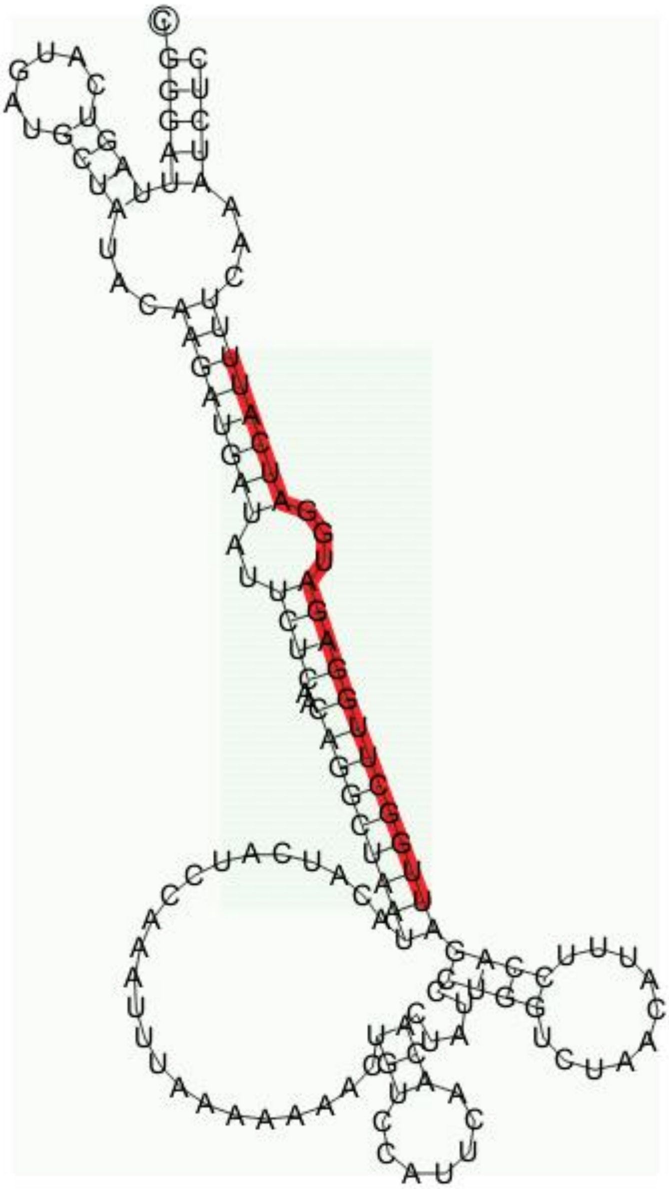

grape-m0014

grape-m0014-3p:TTGGCTTGGAGATGGATCATT

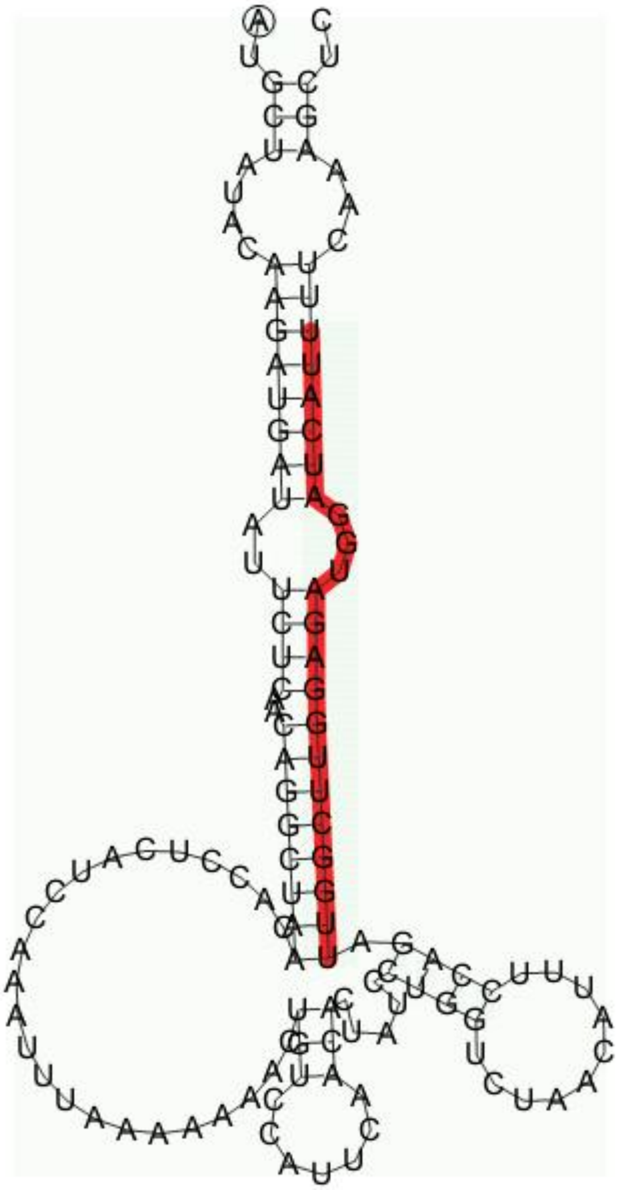

grape-m0015

grape-m0015-3p:TCAATTTGAGAGCTGGAAGAA

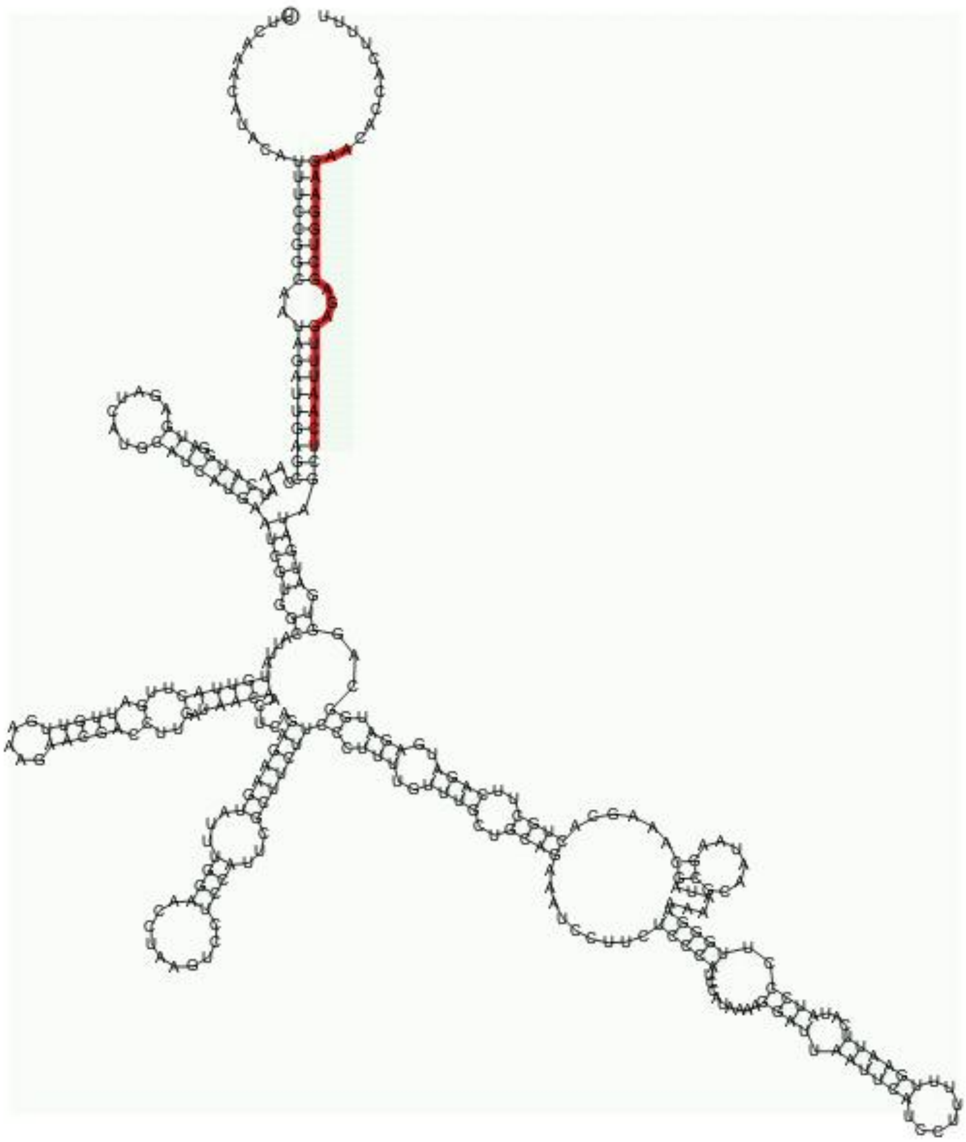

grape-m0016

grape-m0016-3p:ATATTGGTAAATGAATGTTCG

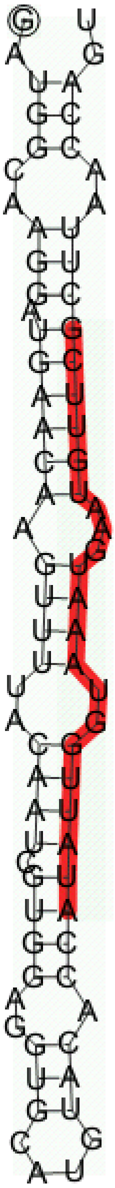

grape-m0017

grape-m0017-3p:AATTTCTTATGTTCATGATTG

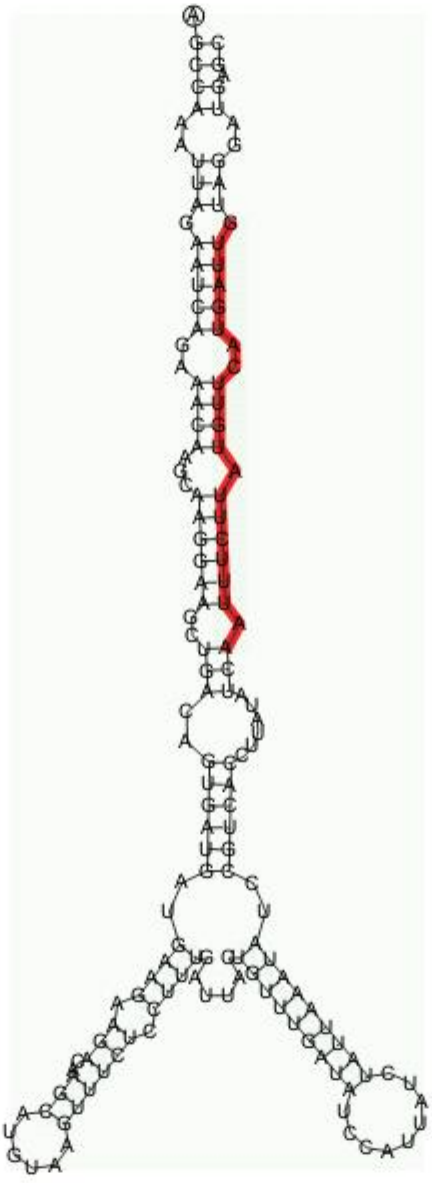

grape-m0018

grape-m0018-3p:AAGAGCAGTTGAACTGAAGCA

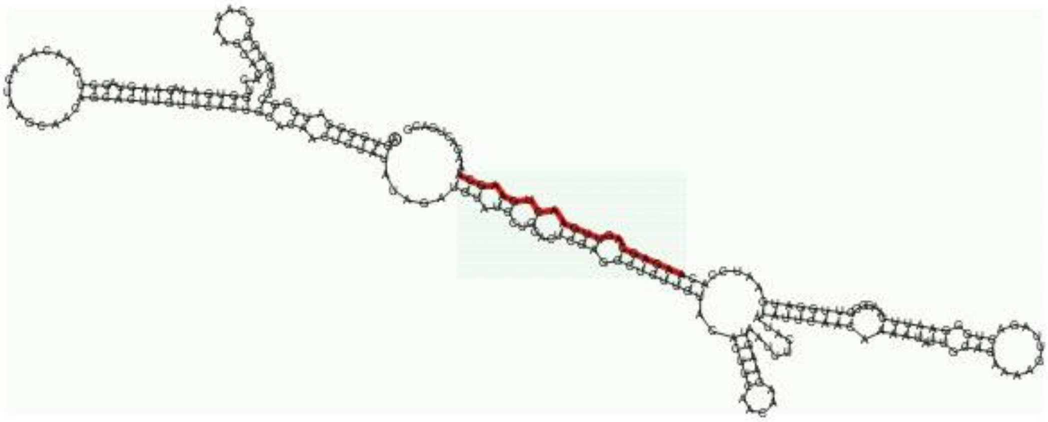

grape-m0019

grape-m0019-5p:TCTGTCGCAGGAGAGATGATGC

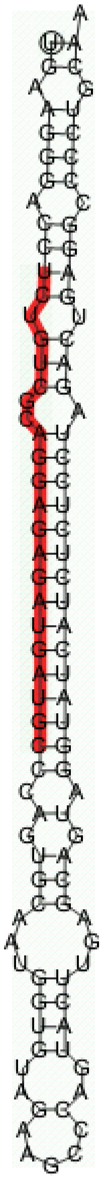

grape-m0020

grape-m0020-5p:GGAATGGGCTGATTGGGATA

grape-m0020-3p:TTCCCAATGCCGCCCATTCCAA

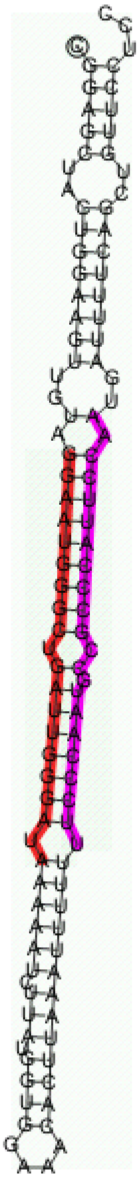

grape-m0021

grape-m0021-5p:CTGAACTCTCTCCCTCATGGCC

grape-m0021-3p:CCAAGAGGGTGGAGTTCAGAT

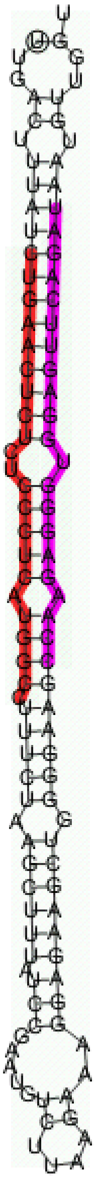

grape-m0022

grape-m0022-5p:AGGAGATGAGGTATGTTTACAT

grape-m0022-3p:CTAAATTGCTTCGGGTCCTGC

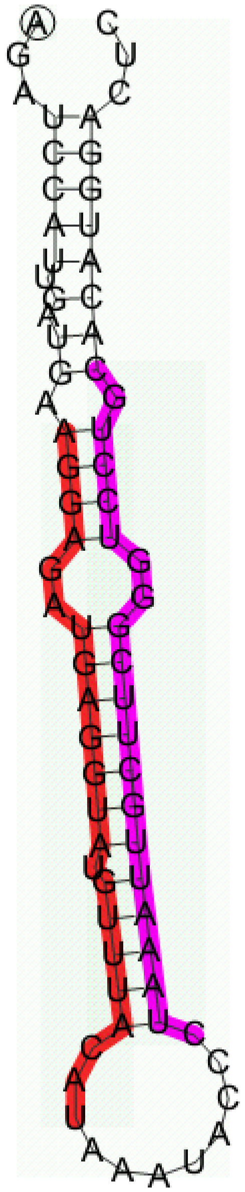

grape-m0023

grape-m0023-5p:AAACATGAGTCTGGACCTTGA

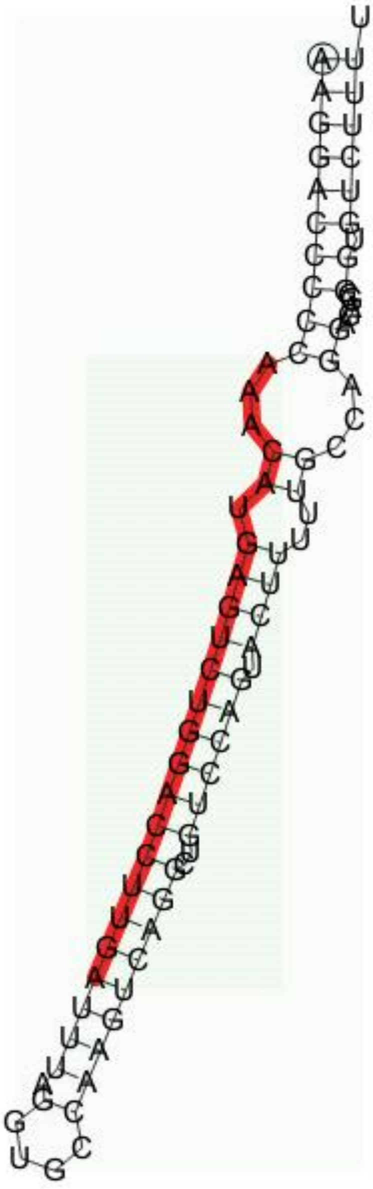

grape-m0024

grape-m0024-5p:AAACATGAGTCTGGACCTTGA

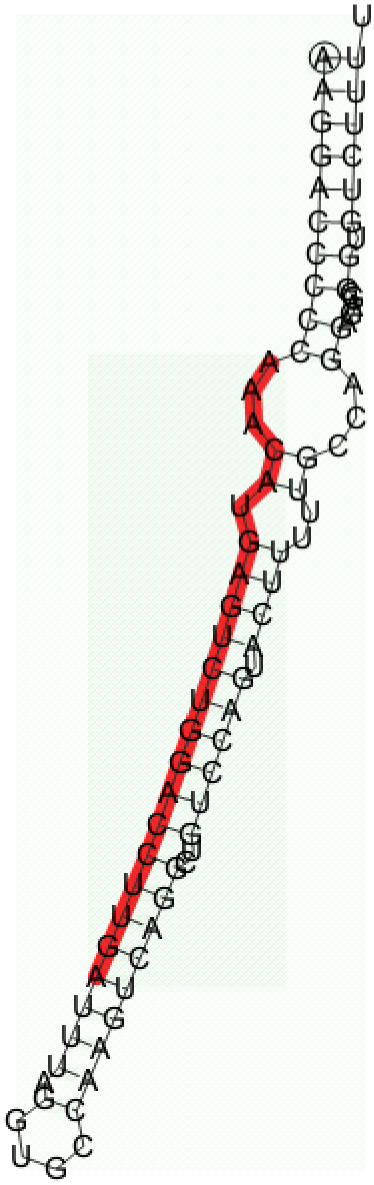

grape-m0025

grape-m0025-5p:TCTGTTTTCACTCTCATTAAG

grape-m0025-3p:TAGTGAGAATGAGTTGGGGAAG

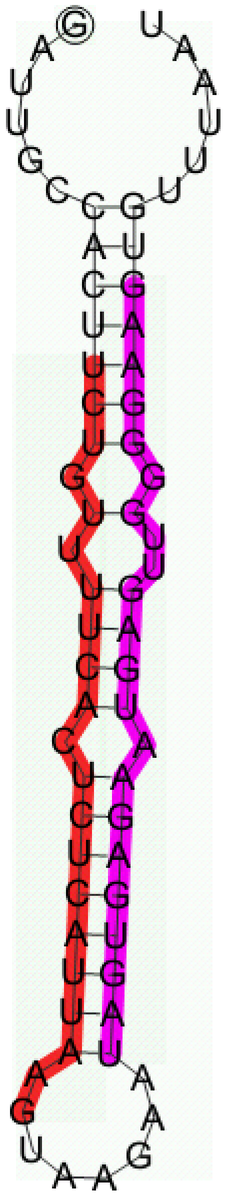

grape-m0026

grape-m0026-5p:TCGGAGAAGTGTGATGTGTAT

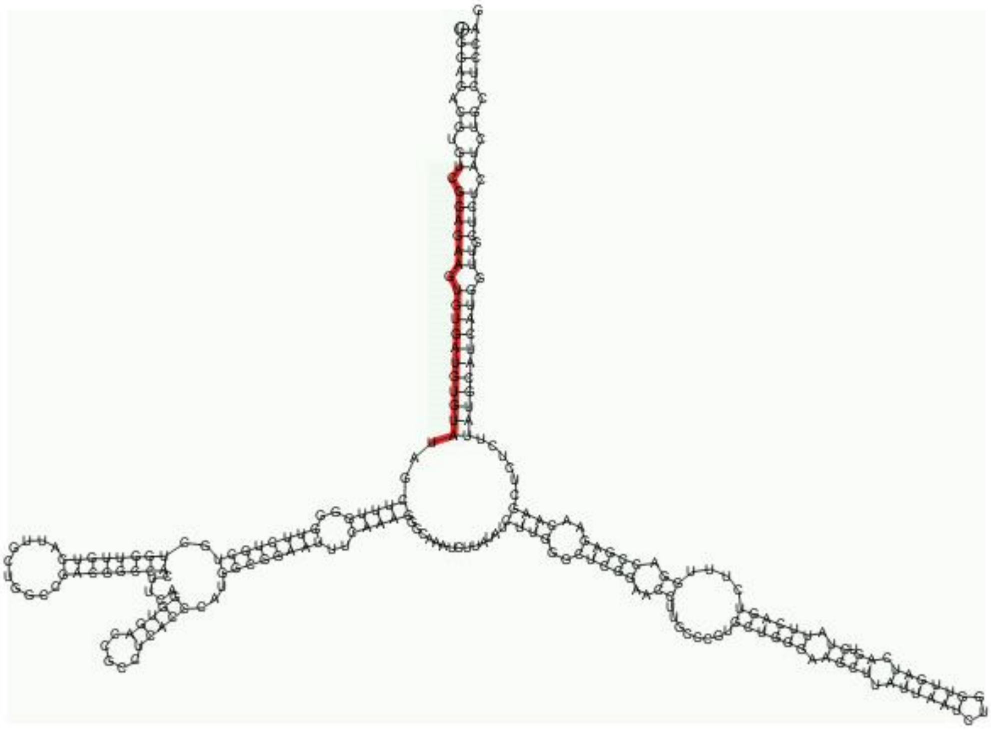

grape-m0027

grape-m0027-5p:ATACCATGTGGAAAAGAGGAATC

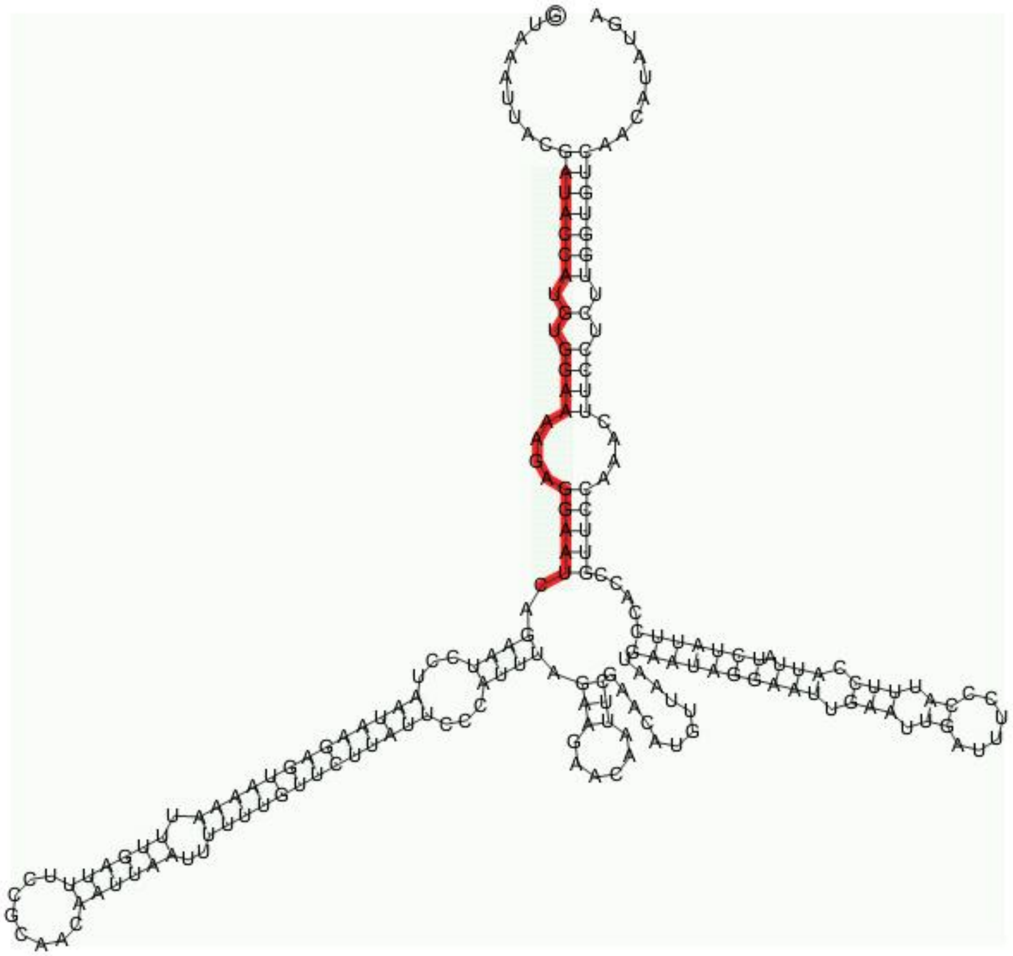

grape-m0028

grape-m0028-3p:ATTGGCAGAATATTCAAGGTTT

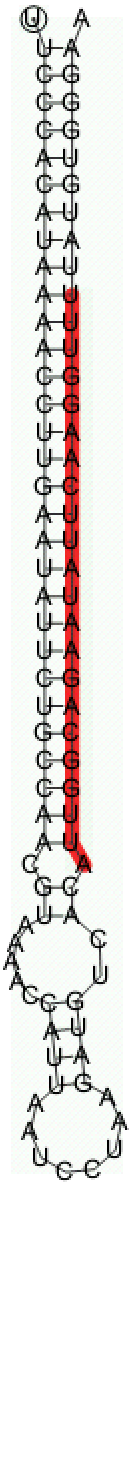

grape-m0029

grape-m0029-3p:TTATTAGGAGGACATTTAGGTAT

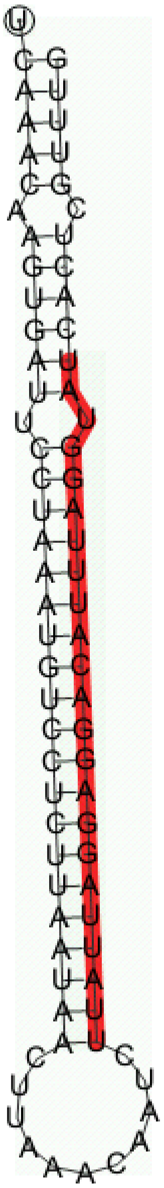

grape-m0030

grape-m0030-5p:TGCGGGTGGAAGAGAAGGAAG

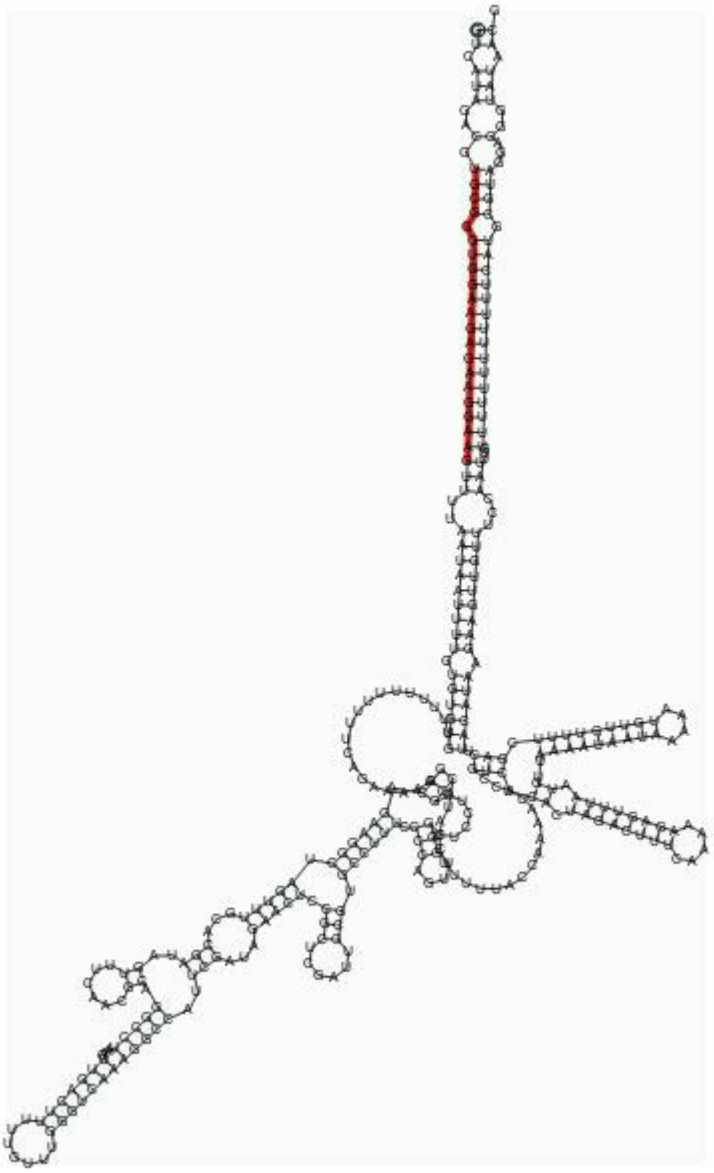

grape-m0031

grape-m0031-3p:TTCCTGCGGTTTCTCGGCGAC

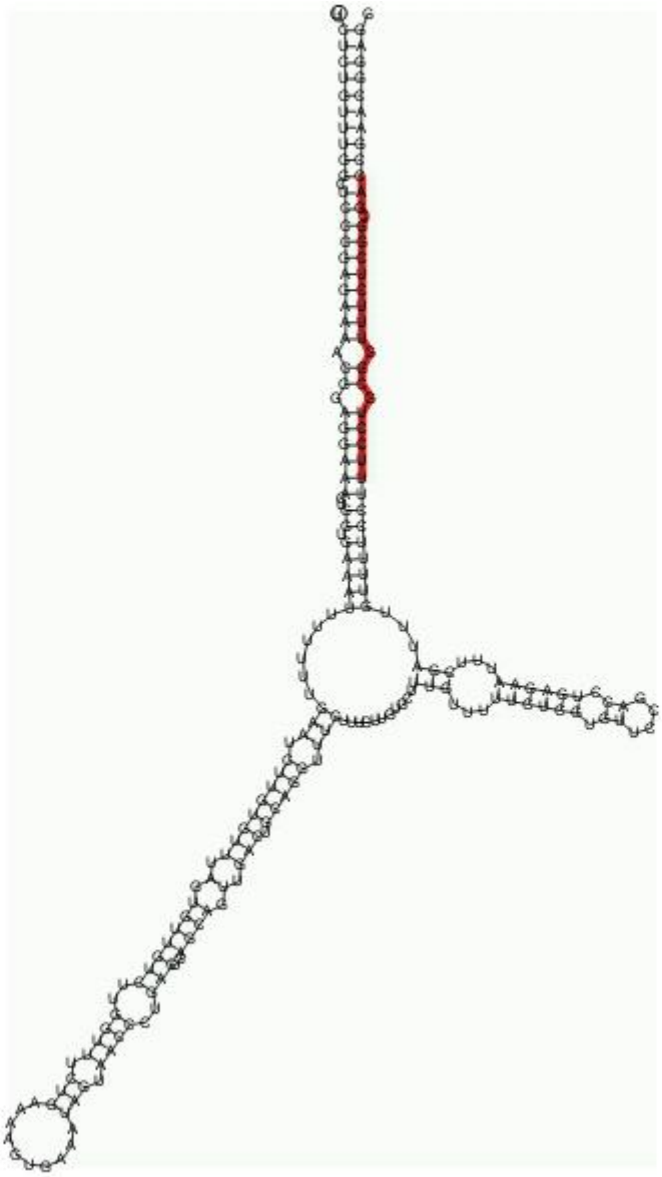

grape-m0032

grape-m0032-5p:CTGGGAAAGCGTGGGAAAACA

grape-m0032-3p:TTTTCCTATGATTTCTTGGCA

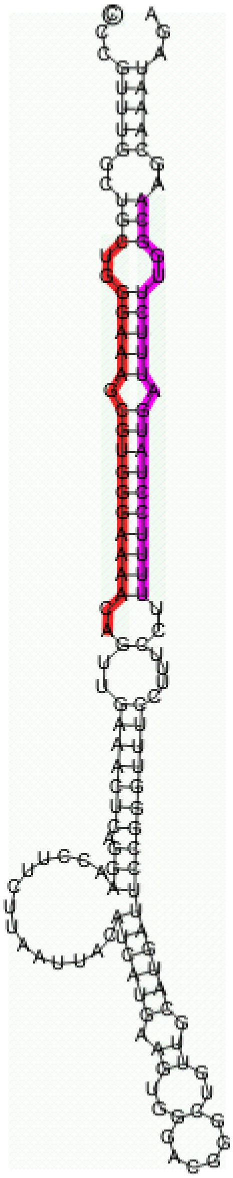

grape-m0033

grape-m0033-3p:TTCCTATCGTTCCCGGGATTT

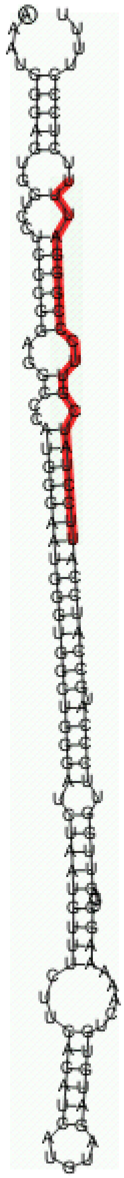

grape-m0034

grape-m0034-5p:TGAAGATAAAGAGTCTCGTCTGG

grape-m0034-3p:TGACCGGCTCTTATCTCTCATG

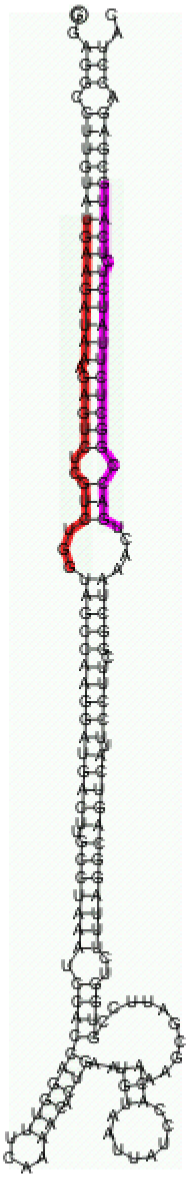

grape-m0035

grape-m0035-3p:GGAATGGATGGCATGGGAACCA

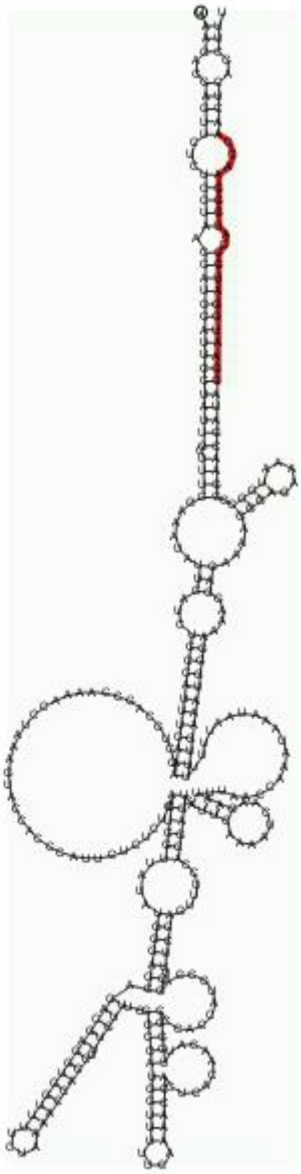

grape-m0036

grape-m0036-5p:TGAGATAAGTCTGCTGCTCCAT

grape-m0036-3p:TGAGTAGTGGACTATCGCATG

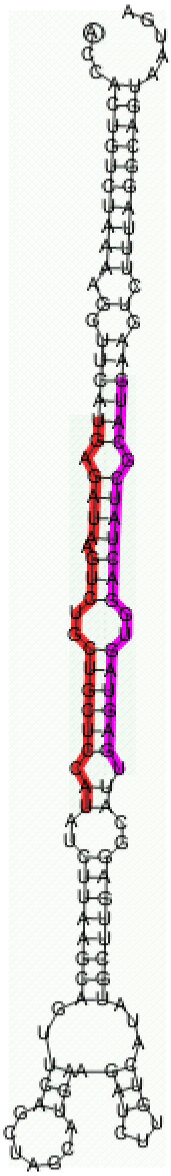

grape-m0037

grape-m0037-5p:GACAAGTTACATACATCCAAG

grape-m0037-3p:TGGATGCATGTAGCTTGTCAA

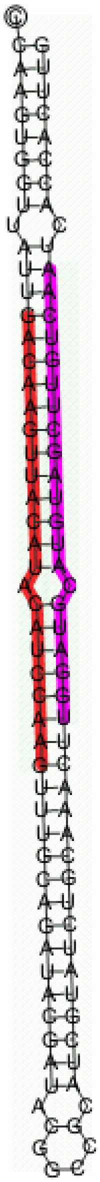

grape-m0038

grape-m0038-5p:TCCTTCGGCGTCGGCAAATCC

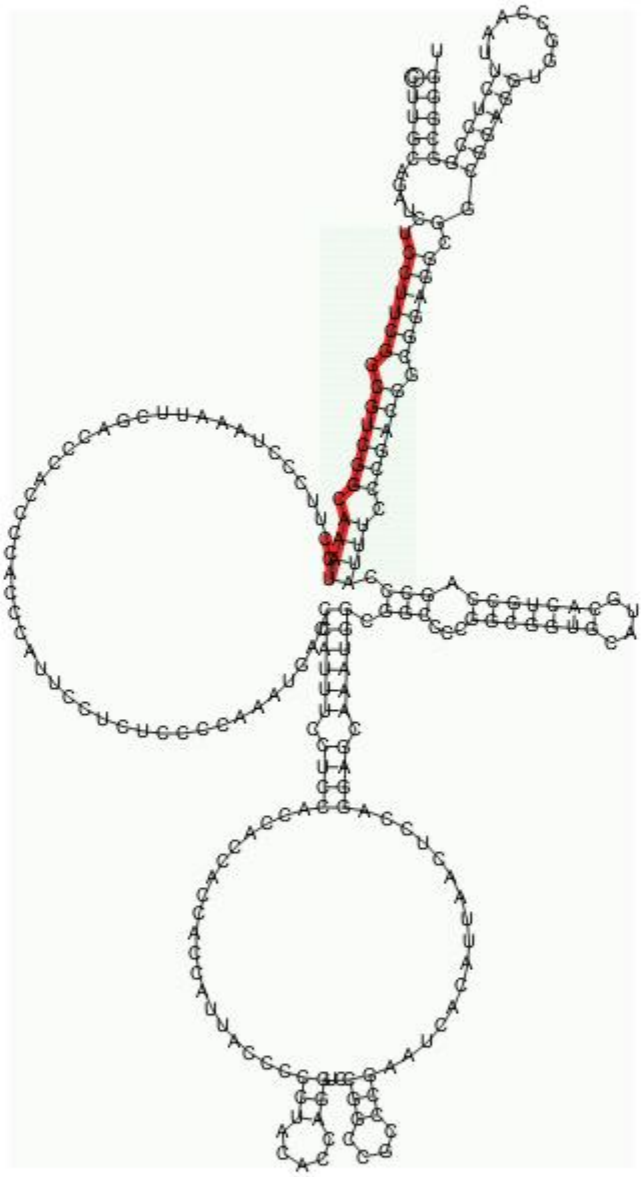

grape-m0039

grape-m0039-5p:AAGGGTTTCTCACAGAGTTTA

grape-m0039-3p:AGCTCTGTTGGACTCTCTTTG

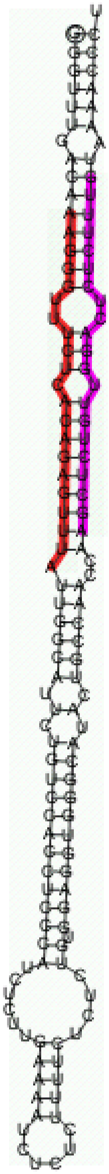

grape-m0040

grape-m0040-3p:GAGGAGAATGTAGTGGGGTTA

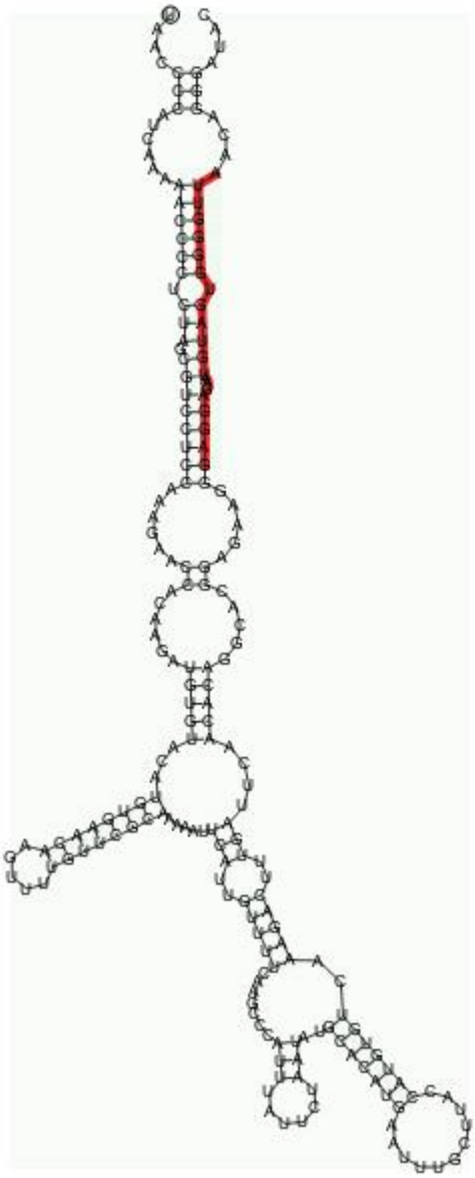

grape-m0041

grape-m0041-5p:CTTTGATCAGATATTGGATTG

grape-m0041-3p:AGCAGAGTTTGATAGAGGGC

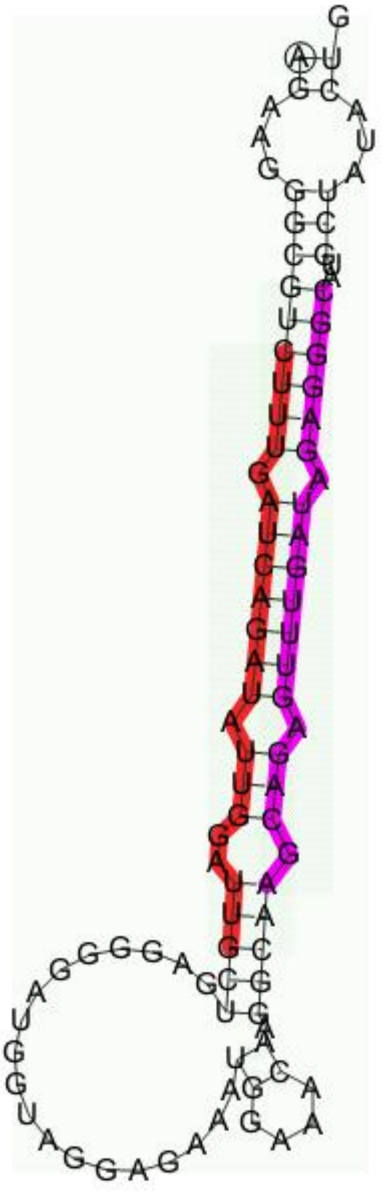

grape-m0042

grape-m0042-5p:AATGACATGAGTTGGAACTAA

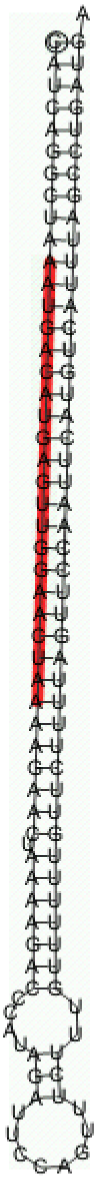

grape-m0043

grape-m0043-3p:GTTGGAAGCCGGTGGGGGACC

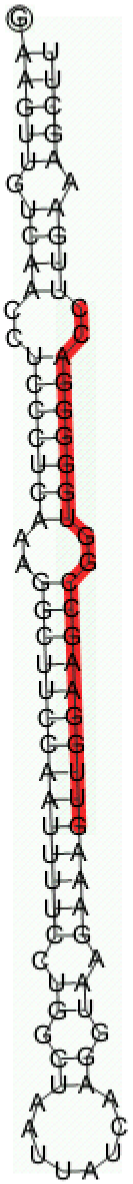

grape-m0044

grape-m0044-3p:GTTGGAAGCCGGTGGGGGACC

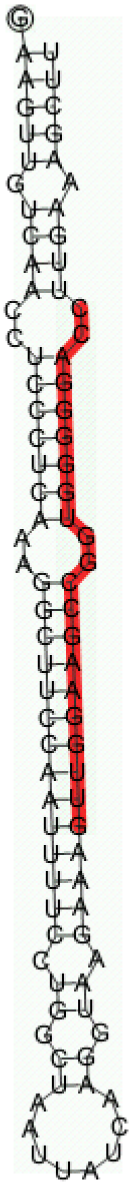

grape-m0045

grape-m0045-3p：GTTGGAAGCCGGTGGGGGACC

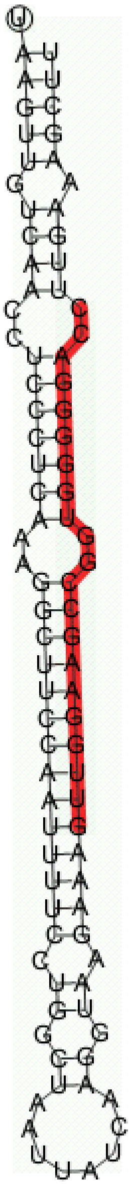

grape-m0046

grape-m0046-5p：GTTGGAAGTCGGTGGGGGAAC

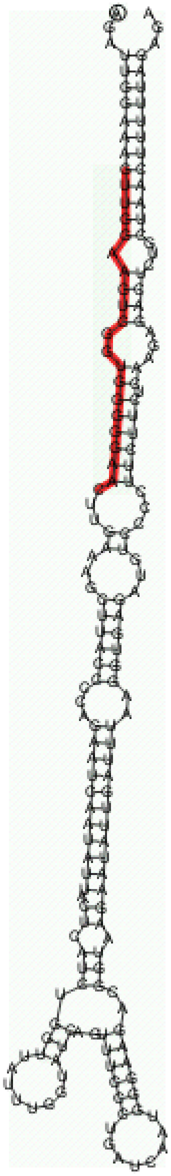

grape-m0047

grape-m0047-3p：GGCGATTGTAAATATGGGTAA

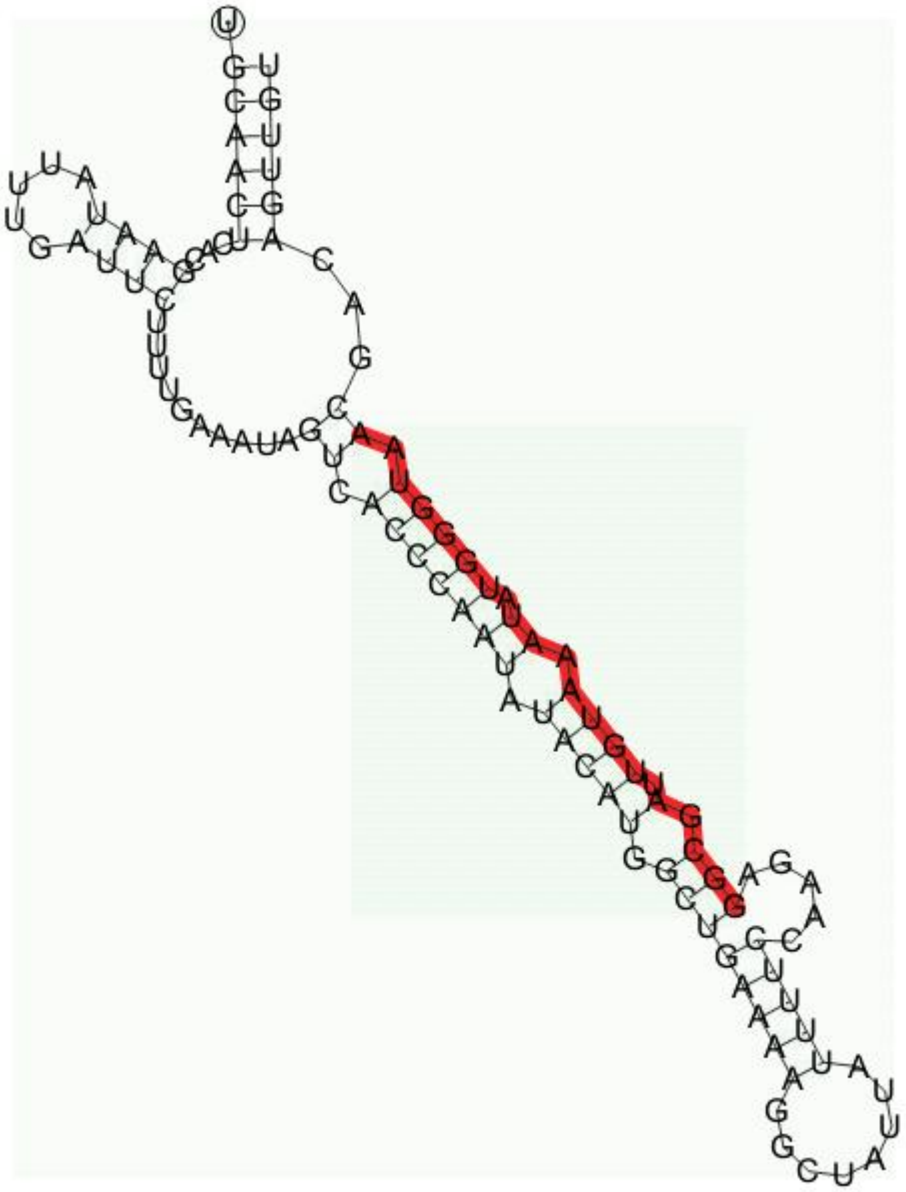

grape-m0048

grape-m0048-3p：TCTAGATTTGGAAGTAGGTCA

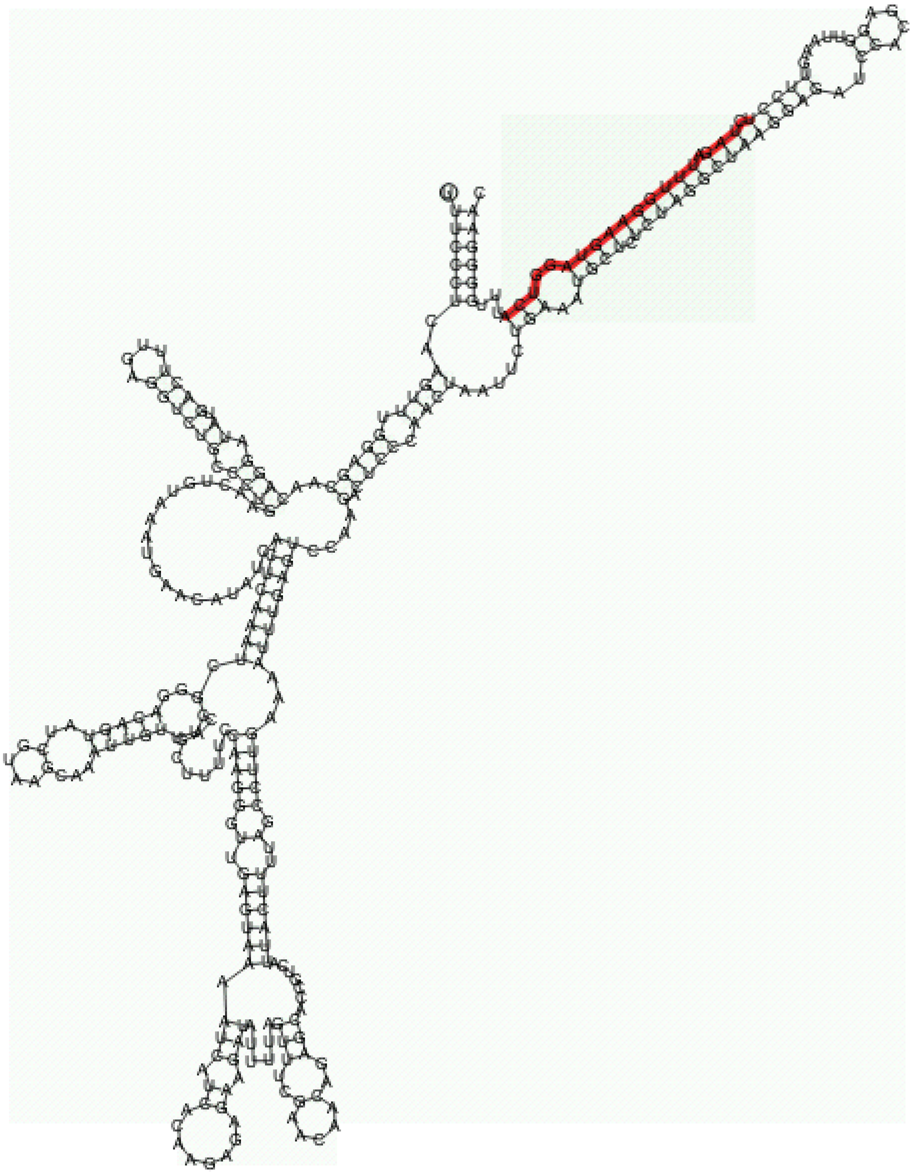

grape-m0049

grape-m0049-5p：GTTGGAAGTCGGTGGGGGACC

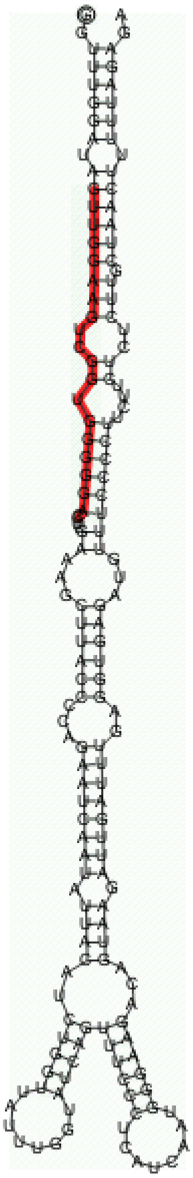

grape-m0050

grape-m0050-3p：GTTGGAAGCCGGTGGGGGACC

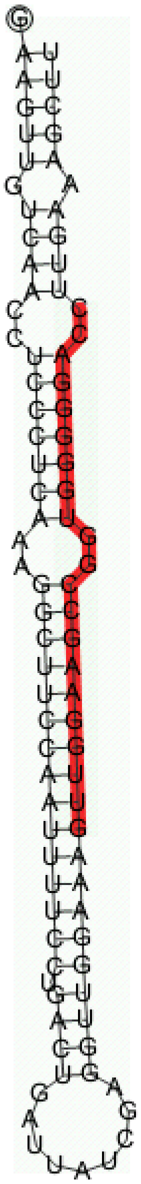

grape-m0051

grape-m0051-5p：TGGGCTTGTGGAGAAGAAAGTGA

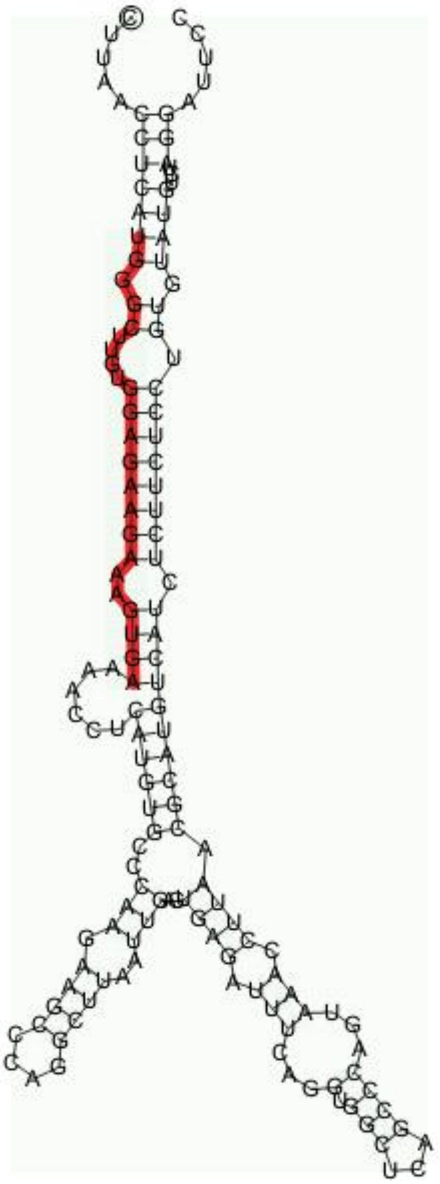

grape-m0052

grape-m0052-5p：CATGGGCGGTTTGGTAAGAGG

grape-m0052-3p：TCTTACCAACACCTCCCATTCC

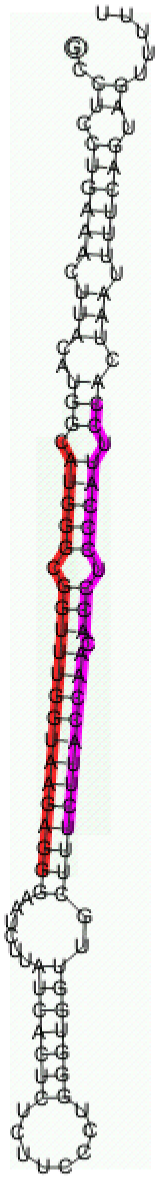

grape-m0053

grape-m0053-5p：GGTATGGGAGGATTGGGGAGA

grape-m0053-3p：TTCCCAAGACCCCCCATGCCAA

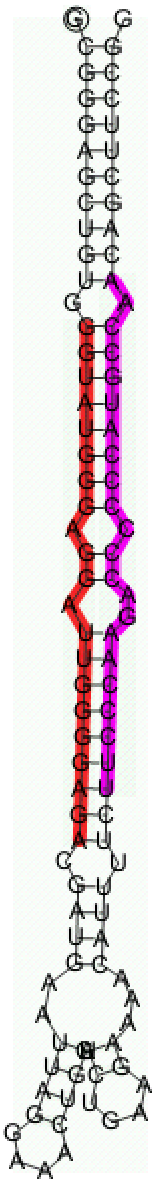

grape-m0054

grape-m0054-5p：TCATACCTCGATCTTCGGTTTC

grape-m0054-3p：AATCTGAGATCGAGAATGAAA

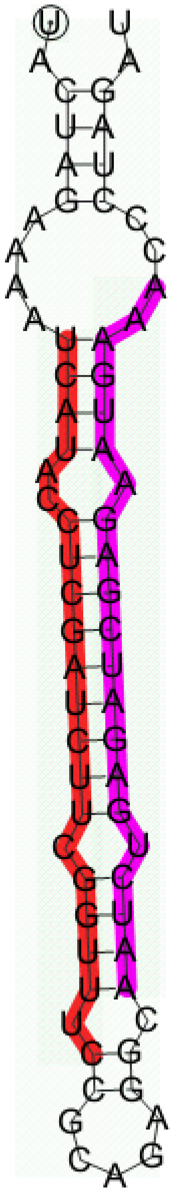

grape-m0055

grape-m0055-3p：ATTCGAACTCAAGACTAAGGT

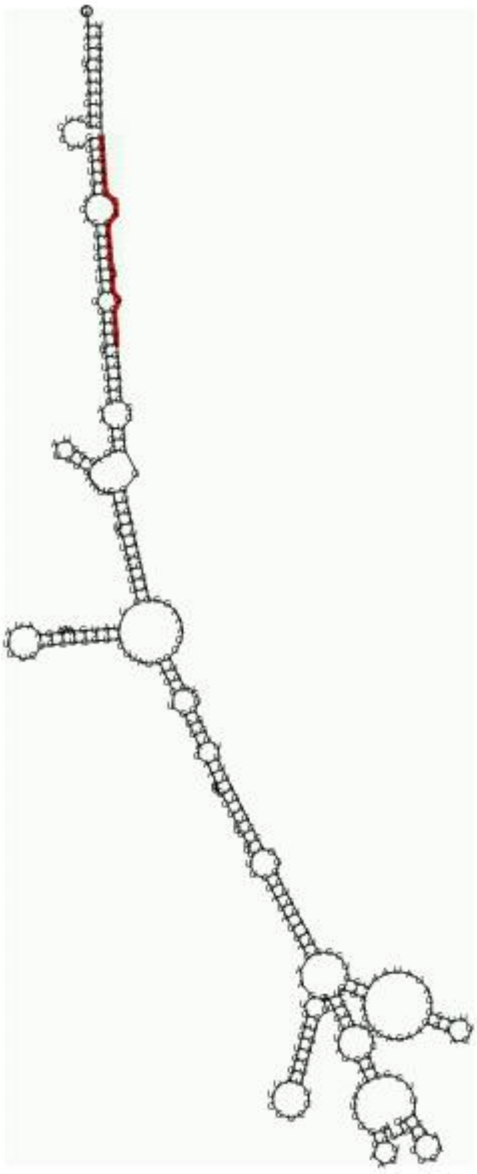

grape-m0056

grape-m0056-5p：ACTCTCCCTCAAGGGCTTCTG

grape-m0056-3p：GAAGCTCTTGAGGGGGACTG

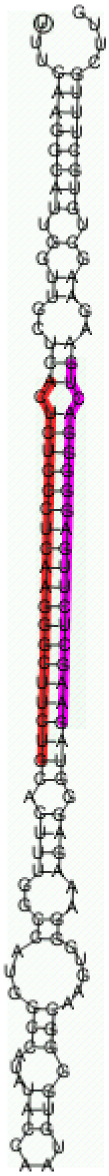

grape-m0057

grape-m0057-3p：AGGTGTAGATGCAAGTGCAGA

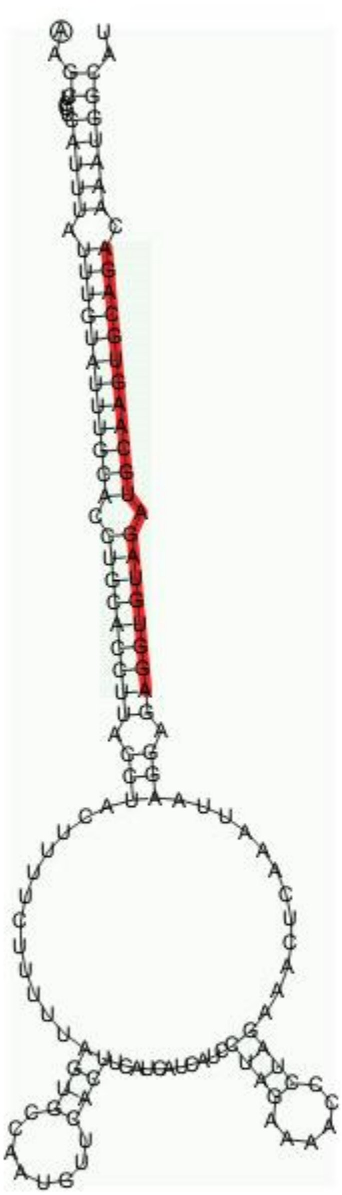

grape-m0058

grape-m0058-3p：TTTAATTTACTAGAGATCTCT

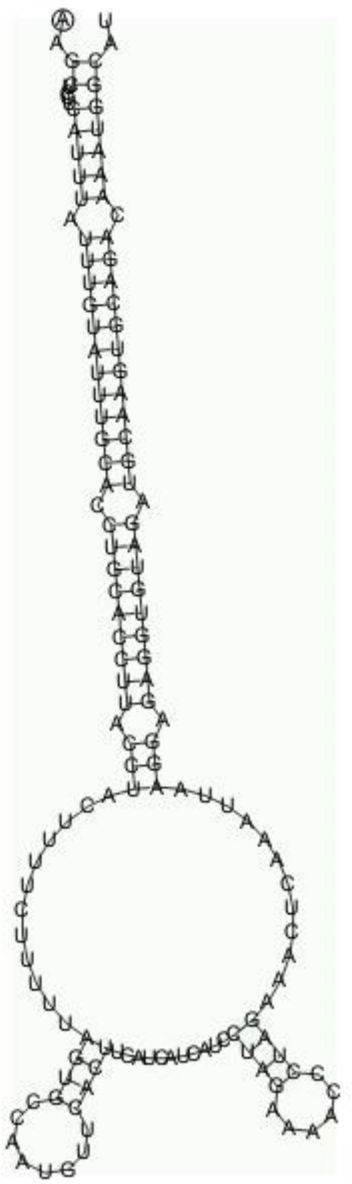

grape-m0059

grape-m0059-3p：GGAGTGAAATTGCAGTGACGG

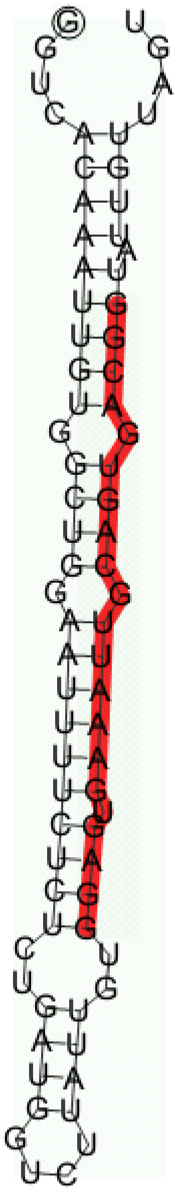

grape-m0060

grape-m0060-3p：TCAGCAGGAATTGGACCAGAA

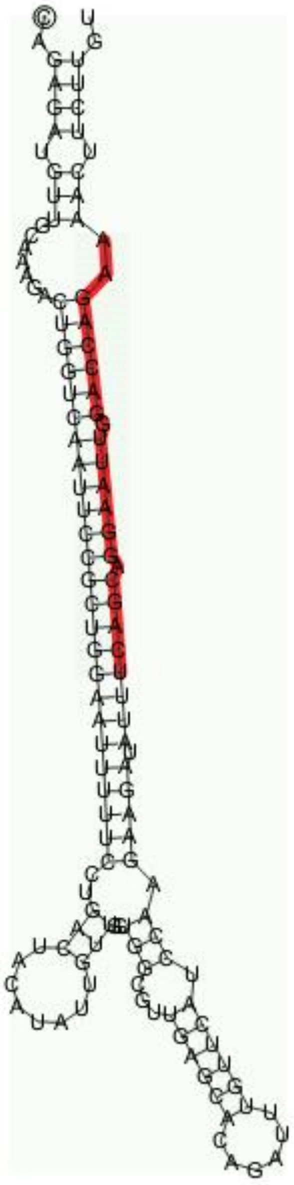

grape-m0061

grape-m0061-5p：ACAGTAGGAAATTGAAAGAGA

grape-m0061-3p：TCTTTCATTTTCCTACTTTTT

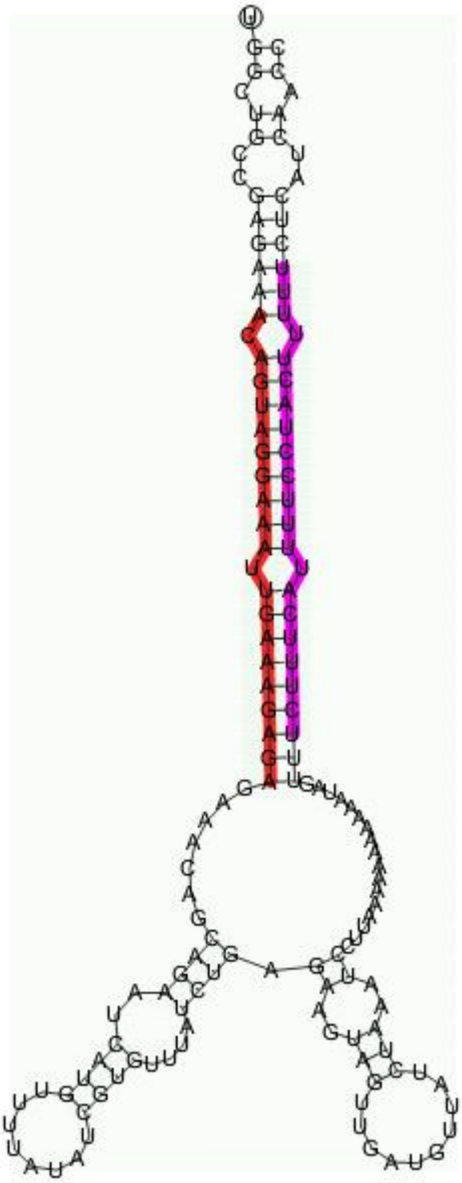

grape-m0062

grape-m0062-3p：AAAGGCGAAGAAAAAGAAGATA

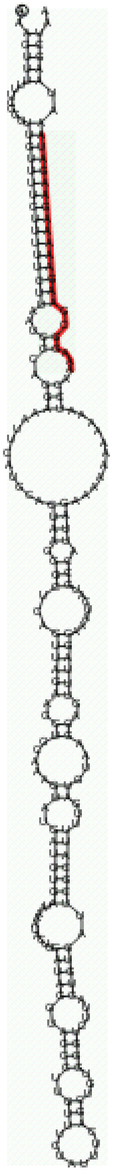

grape-m0063

grape-m0063-5p：AATATGGAGGACTGTGTTCTT

grape-m0063-3p：GAACTCAGTTCCGGTACCATCTTCA

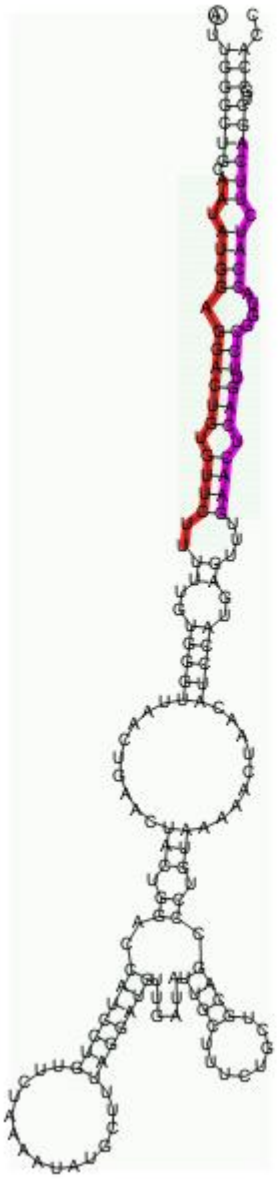

grape-m0064

grape-m0064-3p：TTGGATTCGCGCACAAACTCG

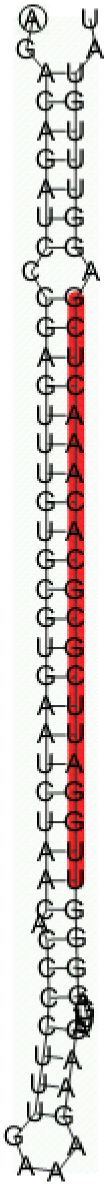

grape-m0065

grape-m0065-3p：TTGGATTCGCGCACAAACTCG

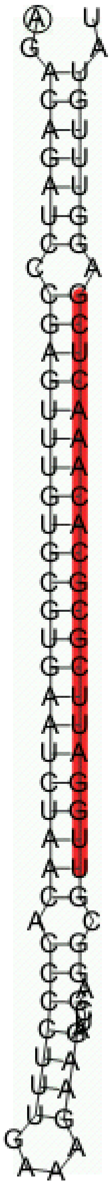

grape-m0066

grape-m0066-5p：CAGCAGTTGCTATTGTGGTTG

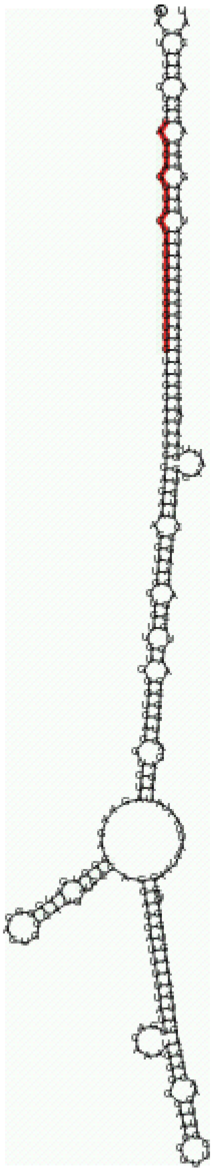

Grape-m0067

grape-m0067-3p：AGAAGAGAGAGAGTACAGCTA

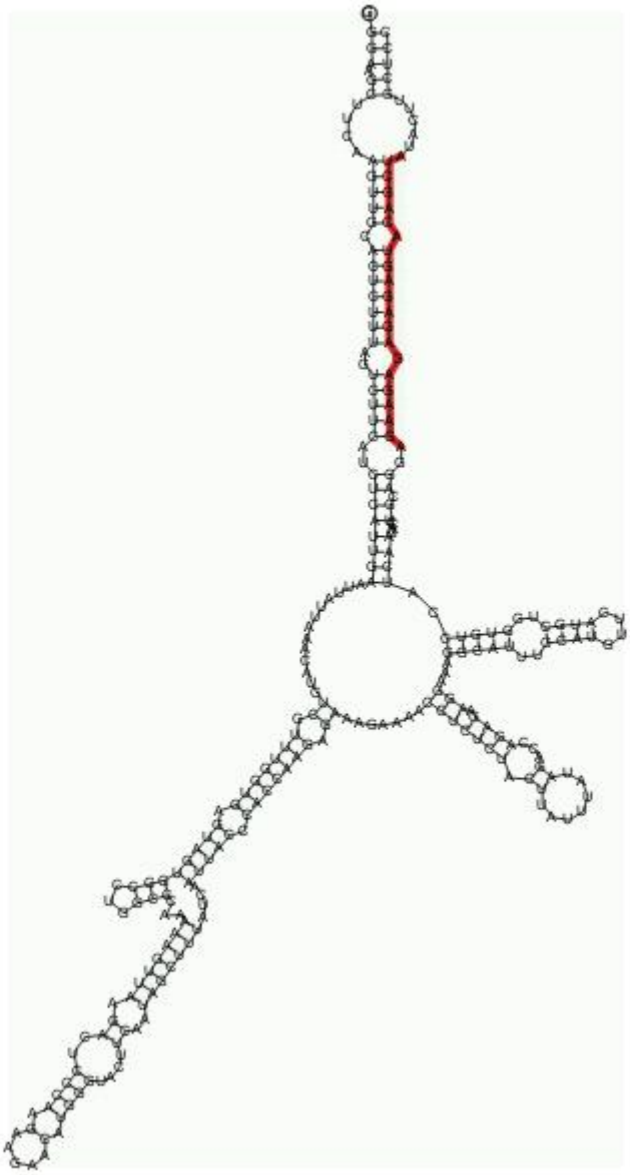

grape-m0068

grape-m0068-5p：TGTTGCCCTCCTGGTACCATC

grape-m0068-3p：TGGTACCAGGAGGGCAACTGTC

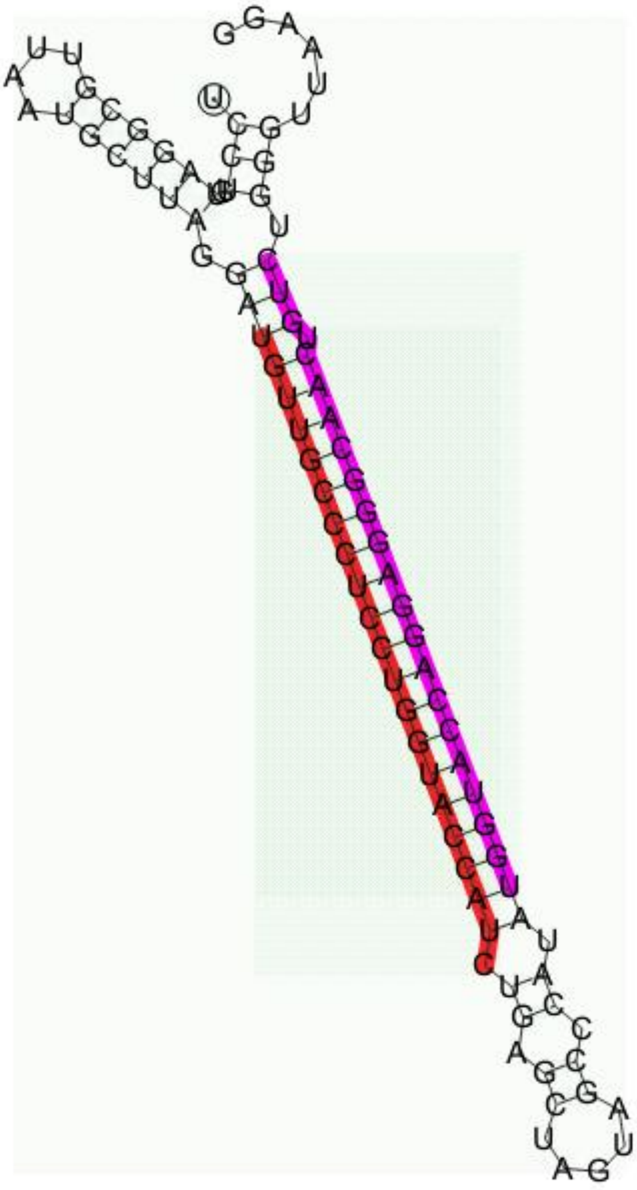

grape-m0069

grape-m0069-5p：TCAAGGGTCGAACGGCTTTGC

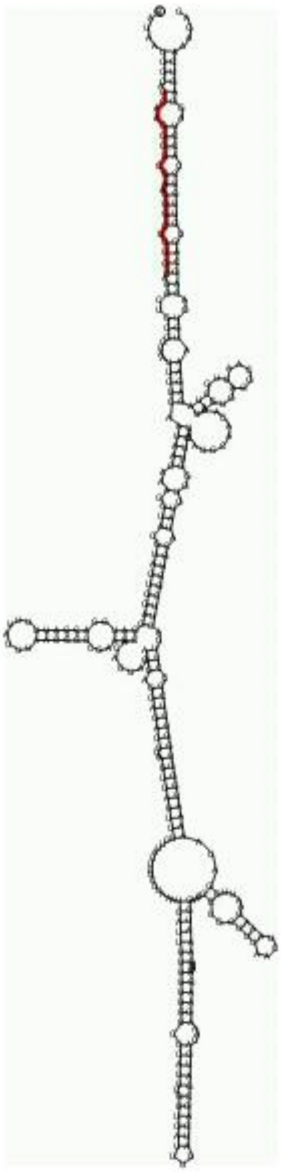

grape-m0070

grape-m0070-3p：TTATGTGAGTGTTCGGCAAATC

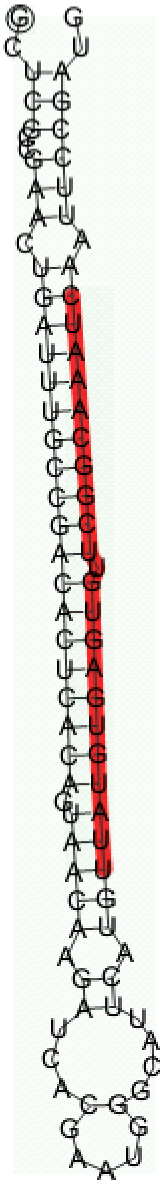

grape-m0071

grape-m0071-5p：TTTTGTTGCTGGTCATCTAGTC

grape-m0071-3p：TTAGATGATCATCAACAAACA

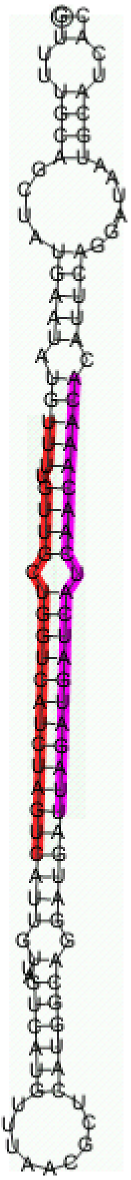

grape-m0072

grape-m0072-5p：TGCTTATTAGGTCTGCTGGCA

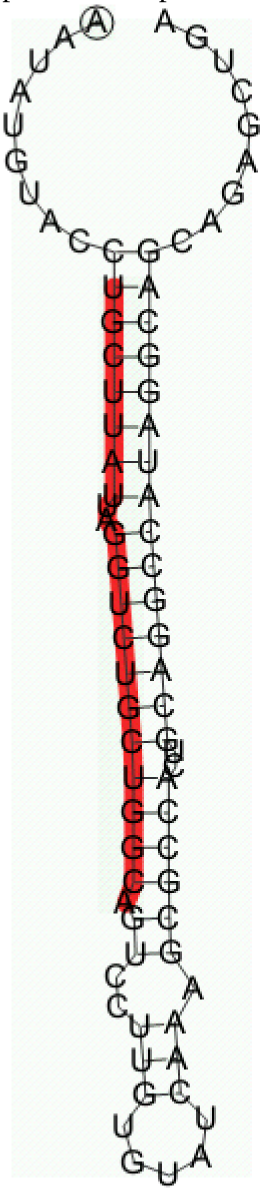

grape-m0073

grape-m0073-5p：TCCATCTTCTCTCTTTTTACA

grape-m0073-3p：TCAAAAGAGAAAATGTGGATG

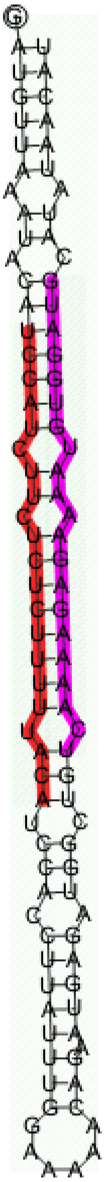

grape-m0074

grape-m0074-5p：TCGCAGGAGAGATGACGCCGT

grape-m0074-3p：AGCATCATTTCTCCTGCATAG

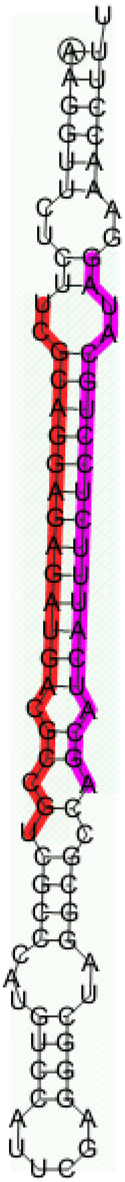

Supplement: Additional file 1 — Secondary structures of the identified novel Vv-miRNAs. [file 1471-2164-15-111-S1.pdf]
