# Supplementary material for: Grapevine microRNAs responsive to exogenous gibberellin
Source: BMC Genomics. 2014 Feb 8;15:111. doi: 10.1186/1471-2164-15-111 (PMC3937062; doi:10.1186/1471-2164-15-111)
Supplement: Additional file 2 — Novel Vv-miRNAs identified in GA3treated and the control grapevine berries. [file 1471-2164-15-111-S2.pdf]

Table S1 Novel Vv-miRNAs identified in GA treatment and Control

miRNA	Mature sequence	Length (nt)	Frequency	Location of precursor shown as start and end on chromosome	MFE (kcal/mol)	Location 3p or 5p	Precursors with fold back*	qRT-PCR
Vv-miRC01	CTATGTTATAGGATCTTGGAT	21	197	chr10:2595357:2595455:-	-51.8	5p	Y	N
Vv-miRC01*	CCAAGATACTATAACATGGTC	21	2	chr10:2595357:2595455:-	-51.8	3p	Y	N
Vv-miRC02	TCCCTTTGGAAGTGCTAAGCG	21	21	chr11:2925877:2926044:-	-93.3	3p	Y	Y
Vv-miRC03	AGTGGTGGCAAGGATGAGCAA	21	6	chr12:17637554:17637759:+	-55.43	3p	Y	Y
Vv-miRC04	TTTGGAATGATTTGTTGATGA	21	17	chr12:17880948:17881049:+	-19.9	5p	Y	Y
Vv-miRC05	AAGATCTCCCATTGCATCTGA	21	6	chr12:19856702:19856829:+	-28.8	3p	Y	Y
Vv-miRC06	TTTTTTGGTTATGGTTGGCTG	21	16	chr12:1137532:1137662:-	-32.3	3p	Y	Y
Vv-miRC07	CTCAAGAAAGCTGTGGGAAAA	21	12	chr12:5155959:5156071:-	-49.5	3p	Y	N
Vv-miRC07*	TTTCCACATCTTTCTTGAACT	21	2	chr12:5155959:5156071:-	-49.5	5p	Y	N
Vv-miRC08	AGAAGAACAAGTAGACTGAGC	21	11	chr12:19456317:19456411:-	-35.6	3p	Y	Y
Vv-miRC09	TTATATAGGCTTTGAGGATGGA	22	22	chr13:9059798:9059972:+	-40.9	3p	Y	Y
Vv-miRC10	TTTTAAAAAGGTTCGTCATTC	21	10	chr13:20001199:20001295:+	-23.1	5p	Y	Y
Vv-miRC11	CCGTGACAAGTGGTATCAGAG	21	13	chr13:6271917:6272017:-	-33	3p	Y	N
Vv-miRC12	TCTGAAGTTTGAAGAGCTGTG	21	121	chr13:9287822:9287908:-	-18.54	5p	Y	N
Vv-miRC12*	AGAGCAATCTACGAACAACAGGAA	24	1	chr13:9287822:9287908:-	-18.54	3p	Y	N
Vv-miRC13	TTGGCTTGGAGATGGATCATT	21	121	chr13:23694657:23694795:-	-25.2	3p	Y	Y
Vv-miRC14	TTGGCTTGGAGATGGATCATT	21	121	chr13:24166889:24167013:-	-24.5	3p	Y	Y
Vv-miRC15	TCAATTTGAGAGCTGGAAGAA	21	8	chr13_random:2451486:2451802:-	-78.6	3p	Y	Y
Vv-miRC16	ATATTGGTAAATGAATGTTCG	21	16	chr13_random:3260493:3260571:-	-19.7	3p	Y	Y
Vv-miRC17	AATTTCTTATGTTCATGATTG	21	9	chr14:1037412:1037567:+	-29.4	3p	Y	Y
Vv-miRC18	AAGAGCAGTTGAACTGAAGCA	21	18	chr14:11961780:11962017:+	-57.39	3p	Y	Y
Vv-miRC19	TCTGTCGCAGGAGAGATGATGC	22	48	chr14:28620631:28620733:+	-53.9	5p	Y	Y

Vv-miRC20	GGAATGGGCTGATTGGGATA	20	8710	chr14:19755471:19755583:-	-57.9	5p	Y	N
Vv-miRC20*	TTCCCAATGCCGCCCATTCCAA	22	2390	chr14:19755471:19755583:-	-57.9	3p	Y	N
Vv-miRC21	CCAAGAGGGTGGAGTTCAGAT	21	17	chr14:24560622:24560730:-	-53.8	3p	Y	N
Vv-miRC21*	CTGAACTCTCTCCCTCATGGCC	22	10	chr14:24560622:24560730:-	-53.8	5p	Y	N
Vv-miRC22	CTAAATTGCTTCGGGTCCTGC	21	76	chr15:10683797:10683870:+	-18.8	3p	Y	N
Vv-miRC22*	AGGAGATGAGGTATGTTTACAT	22	68	chr15:10683797:10683870:+	-18.8	5p	Y	N
Vv-miRC23	AAACATGAGTCTGGACCTTGA	21	9	chr15:5644102:5644187:-	-26.5	5p	Y	Y
Vv-miRC24	AAACATGAGTCTGGACCTTGA	21	9	chr15:5686498:5686583:-	-26.5	5p	Y	Y
Vv-miRC25	TCTGTTTTCACTCTCATTAAG	21	13	chr15:19955164:19955230:-	-20.8	5p	Y	N
Vv-miRC25*	TAGTGAGAATGAGTTGGGGAAG	22	1	chr15:19955164:19955230:-	-20.8	3p	Y	N
Vv-miRC26	TCGGAGAAGTGTGATGTGTAT	21	8	chr16:14731376:14731622:+	-83.2	5p	Y	Y
Vv-miRC27	ATACCATGTGGAAAAGAGGAATC	23	67	chr16:2126040:2126238:-	-38.7	5p	Y	Y
Vv-miRC28	ATTGGCAGAATATTCAAGGTTT	22	10	chr16:3111476:3111566:-	-51.7	3p	Y	N
Vv-miRC29	TTATTAGGAGGACATTTAGGTAT	23	40	chr16:14895040:14895117:-	-39.8	3p	Y	Y
Vv-miRC30	TGCGGGTGGAAGAGAAGGAAG	21	40	chr16:17808410:17808741:-	-79	5p	Y	Y
Vv-miRC31	TTCCTGCGGTTTCTCGGCGAC	21	11	chr16:19208159:19208370:-	-67.7	3p	Y	Y
Vv-miRC32	TTTTCCTATGATTTCTTGGCA	21	9	chr17:4716715:4716853:+	-44.23	3p	Y	N
Vv-miRC32*	CTGGGAAAGCGTGGGAAAACA	21	1	chr17:4716715:4716853:+	-44.23	5p	Y	N
Vv-miRC33	TTCCTATCGTTCCCGGGATTT	21	14	chr17:5521552:5521691:+	-69.3	3p	Y	N
Vv-miRC34	TGACCGGCTCTTATCTCTCATG	22	45	chr17:355691:355861:-	-68.7	3p	Y	Y
Vv-miRC34*	TGAAGATAAAGAGTCTCGTCTGG	23	1	chr17:355691:355861:-	-68.7	5p	Y	N
Vv-miRC35	GGAATGGATGGCATGGGAACCA	22	6	chr17:5521633:5521968:-	-105.4	3p	Y	N
Vv-miRC36	TGAGTAGTGGACTATCGCATG	21	19803	chr17:7265146:7265278:-	-54.7	3p	Y	Y
Vv-miRC36*	TGAGATAAGTCTGCTGCTCCAT	22	1086	chr17:7265146:7265278:-	-54.7	5p	Y	N
Vv-miRC37	TGGATGCATGTAGCTTGTCAA	21	10	chr18:4079210:4079312:+	-71.9	3p	Y	Y
Vv-miRC37*	GACAAGTTACATACATCCAAG	21	2	chr18:4079210:4079312:+	-71.9	5p	Y	N

Vv-miRC38	TCCTTCGGCGTCGGCAAATCC	21	14	chr18:29129189:29129421:+	-68.2	5p	Y	Y
Vv-miRC39	AAGGGTTTCTCACAGAGTTTA	21	9	chr18:1413017:1413151:-	-66.9	5p	Y	N
Vv-miRC39*	AGCTCTGTTGGACTCTCTTTG	21	2	chr18:1413017:1413151:-	-66.9	3p	Y	N
Vv-miRC40	GAGGAGAATGTAGTGGGGTTA	21	5	chr18_random:4558402:4558602:+	-51.3	3p	Y	N
Vv-miRC41	CTTTGATCAGATATTGGATTG	21	16	chr19:607158:607253:+	-21.85	5p	Y	N
Vv-miRC41*	AGCAGAGTTTGATAGAGGGC	20	1	chr19:607158:607253:+	-21.85	3p	Y	N
Vv-miRC42	AATGACATGAGTTGGAACTAA	21	10	chr19:8929025:8929134:+	-67.9	5p	Y	N
Vv-miRC43	GTTGGAAGCCGGTGGGGGACC	21	4878	chr19:12889975:128965:+	-44.9	3p	Y	N
Vv-miRC44	GTTGGAAGCCGGTGGGGGACC	21	4878	chr19:13141683:13141773:+	-44.9	3p	Y	N
Vv-miRC45	GTTGGAAGCCGGTGGGGGACC	21	4878	chr19:13510105:13510195:+	-44.7	3p	Y	N
Vv-miRC46	GTTGGAAGTCGGTGGGGGAAC	21	3125	chr19:18678400:18678570:+	-51.9	5p	Y	N
Vv-miRC47	GGCGATTGTAAATATGGGTAA	21	13	chr19:580958:581064:-	-21	3p	Y	N
Vv-miRC48	TCTAGATTTGGAAGTAGGTCA	21	5	chr19:5446765:5447061:-	-68.9	3p	Y	N
Vv-miRC49	GTTGGAAGTCGGTGGGGGACC	21	836	chr19:18872600:18872761:-	-50	5p	Y	N
Vv-miRC50	GTTGGAAGCCGGTGGGGGACC	21	4878	chr19:18881366:18881456:-	-47.9	3p	Y	N
Vv-miRC51	TGGGCTTGTGGAGAAGAAAGTGA	23	6	chr19:22103176:22103318:-	-35.7	5p	Y	N
Vv-miRC52	CATGGGCGGTTTGGTAAGAGG	21	16066	chr1:3865565:3865681:+	-46.2	5p	Y	N
Vv-miRC52*	TCTTACCAACACCTCCCATTCC	22	2274	chr1:3865565:3865681:+	-46.2	3p	Y	N
Vv-miRC53	GGTATGGGAGGATTGGGGAGA	21	5013	chr1:3865822:3865924:+	-58.1	5p	Y	N
Vv-miRC53*	TTCCCAAGACCCCCCATGCCAA	22	3750	chr1:3865822:3865924:+	-58.1	3p	Y	N
Vv-miRC54	TCATACCTCGATCTTCGGTTTC	22	8	chr1:19610984:19611053:+	-24.4	5p	Y	N
Vv-miRC54*	AATCTGAGATCGAGAATGAAA	21	1	chr1:19610984:19611053:+	-24.4	3p	Y	N
Vv-miRC55	ATTCGAACTCAAGACTAAGGT	21	476	chr1:20086070:20086427:-	-94.56	3p	Y	N
Vv-miRC56	GAAGCTCTTGAGGGGGACTG	20	692	chr2:1237534:1237664:+	-64.9	3p	Y	N
Vv-miRC56*	ACTCTCCCTCAAGGGCTTCTG	21	15	chr2:1237534:1237664:+	-64.9	5p	Y	N
Vv-miRC57	AGGTGTAGATGCAAGTGCAGA	21	12	chr2:8092876:8093016:+	-42.8	3p	Y	N

Vv-miRC58	TTTAATTTACTAGAGATCTCT	21	16	chr3:4796795:4797053:+	-61.4	3p	Y	N
Vv-miRC59	GGAGTGAAATTGCAGTGACGG	21	13	chr4:378829:378904:-	-21.1	3p	Y	N
Vv-miRC60	TCAGCAGGAATTGGACCAGAA	21	43	chr4:792817:792945:-	-40.2	3p	Y	N
Vv-miRC61	ACAGTAGGAAATTGAAAGAGA	21	8	chr4:1141379:1141524:-	-38.2	5p	Y	N
Vv-miRC61*	TCTTTCATTTTCCTACTTTTT	20	6	chr4:1141379:1141524:-	-38.2	3p	Y	N
Vv-miRC62	AAAGGCGAAGAAAAAGAAGATA	21	9	chr4:19502753:19502982:-	-49.4	3p	Y	N
Vv-miRC63	AATATGGAGGACTGTGTTCTT	20	20	chr5:20944390:20944559:+	-44.63	5p	Y	N
Vv-miRC63*	GAACTCAGTTCCGGTACCATCTTCA	24	1	chr5:20944390:20944559:+	-44.63	3p	Y	N
Vv-miRC64	TTGGATTCGCGCACAAACTCG	20	13	chr5:166480:166567:-	-52.1	3p	Y	N
Vv-miRC65	TTGGATTCGCGCACAAACTCG	20	13	chr5:600179:600266:-	-48.9	3p	Y	N
Vv-miRC66	CAGCAGTTGCTATTGTGGTTG	20	96	chr5:6017515:6017763:-	-98.3	5p	Y	N
Vv-miRC67	AGAAGAGAGAGAGTACAGCTA	20	110	chr5:19124470:19124728:-	-65.6	3p	Y	N
Vv-miRC68	TGGTACCAGGAGGGCAACTGTC	21	12	chr5:20264917:20265010:-	-50.4	3p	Y	N
Vv-miRC68*	TGTTGCCCTCCTGGTACCATC	20	1	chr5:20264917:20265010:-	-50.4	5p	Y	N
Vv-miRC69	TCAAGGGTCGAACGGCTTTGC	20	27	chr5:21653204:21653548:-	-87.8	5p	Y	N
Vv-miRC70	TTATGTGAGTGTTCGGCAAATC	21	30	chr5:22090345:22090434:-	-37.9	3p	Y	N
Vv-miRC71	TTAGATGATCATCAACAAACA	20	5931	chr5:24742118:24742235:-	-45.5	3p	Y	N
Vv-miRC71*	TTTTGTTGCTGGTCATCTAGTC	21	35	chr5:24742118:24742235:-	-45.5	5p	Y	N
Vv-miRC72	TGCTTATTAGGTCTGCTGGCA	20	7	chr6:1876410:1876488:+	-27.3	5p	Y	N
Vv-miRC73	TCAAAAGAGAAAATGTGGATG	20	9	chr6:4485456:4485555:+	-35.9	3p	Y	N
Vv-miRC73*	TCCATCTTCTCTCTTTTTACA	20	1	chr6:4485456:4485555:+	-35.9	5p	Y	N
Vv-miRC74	TCGCAGGAGAGATGACGCCGT	20	1263	chr6:3976407:3976496:-	-48.4	5p	Y	N
Vv-miRC74*	AGCATCATTTCTCCTGCATAG	20	49	chr6:3976407:3976496:-	-48.4	3p	Y	N
Vv-miRC75	ATATTAGCAGCTGAGAACACA	21	16	chr14:22335448:22335597:+	-71.7	3p	Y	N
Vv-miRC76	CAGGACTGGCAGTGATGGTTA	21	13	chr19:5046231:5046495:+	-67.97	5p	Y	N
Vv-miRC77	GTGTTTTGCAGGATCAGACGG	21	8	chr1:10111677:10111963:+	-64.31	5p	Y	N

Vv-miRC78	TGGCTGAGAACTTGATGGTTA	21	31	chr1:10113504:10113829:+	-68	3p	Y	N
Vv-miRC79	TTCAAGTCAAAGTCGAACAAG	21	10	chr1:22552197:22552267:-	-20.2	5p	Y	N
Vv-miRC80	AGCGAAGTAGTTGTAGGGCTT	21	11	chr2:4101786:4101936:+	-68.3	5p	Y	N
Vv-miRC81	TTCGGAGGGAACTGACCGGTT	21	8	chr2:4105677:4105745:+	-25.7	5p	Y	N
Vv-miRC82	TGCCAAGAAGCACATTCCTCC	21	193	chr3:17003551:17003669:-	-81.3	5p	Y	N
Vv-miRC82*	AGGAATGTGCTTCTTGGCATA	21	1	chr3:17003551:17003669:-	-81.3	3p	Y	N
Vv-miRC83	CAAGTGTGGGATTTTGGGTGGCT	23	6	chr4:20294006:20294192:+	-59.2	5p	Y	N
Vv-miRC84	GCAGCATCATGAAGATTCACA	20	6	chr6:6003607:6003738:+	-61.57	5p	Y	N
Vv-miRC84*	GGAATCTTGATGATGCTGCAT	20	2	chr6:6003607:6003738:+	-61.57	3p	Y	N
Vv-miRC85	AGGTGCAGGTGAAGGTGCAGA	20	20	chr6:17896119:17896283:+	-75.22	3p	Y	N
Vv-miRC85*	TGCATTTGCACCTGCACCTTA	20	11	chr6:17896119:17896283:+	-75.22	5p	Y	N
Vv-miRC86	GTAGCATCATCAAGATTCACA	20	19	chr6:17652412:17652519:-	-46.5	5p	Y	N
Vv-miRC87	GGAATGTTGTCTGGCTCGAGGT	21	8	chr7:19450050:19450214:+	-49.6	3p	Y	N
Vv-miRC88	ATGTATTTGAGGGAAAGCAAA	20	5	chr8:22308229:22308469:+	-68.25	3p	Y	N
Vv-miRC88*	TGTTTTCCCTCAAAAACATGT	20	1	chr8:22308229:22308469:+	-68.25	5p	Y	N
Vv-miRC89	CTGCGGGTGGAAAAGGATTAGGC	22	78	chr8:14079511:14079769:-	-106.45	5p	Y	N
Vv-miRC89*	CTCATCCTTTTCCATCGGCAGCA	22	4	chr8:14079511:14079769:-	-106.45	3p	Y	N
Vv-miRC90	TCTCAGCAACCAAGTAGAGCC	20	68	chr8:20492988:20493218:-	-60.2	3p	Y	N
