# Supplementary material for: Grapevine microRNAs responsive to exogenous gibberellin
Source: BMC Genomics. 2014 Feb 8;15:111. doi: 10.1186/1471-2164-15-111 (PMC3937062; doi:10.1186/1471-2164-15-111)
Supplement: Additional file 3 — List of predicted target genes of novel miRNAs identified in grapevines. [file 1471-2164-15-111-S3.doc]

Table S2 List of primers used in qRT-PCR

| **miRNA** | **specific primers (5’ → 3’)** | **adaptor primers (5’ → 3’)** |
| --- | --- | --- |
| miRC01 | CTATGTTATAGGATCTTGGAT | ATTCTAGAGGCCGAGGCGGCCGACATG |
| miRC01* | CCAAGATACTATAACATGGTC | ATTCTAGAGGCCGAGGCGGCCGACATG |
| miRC02 | TCCCTTTGGAAGTGCTAAGCG | ATTCTAGAGGCCGAGGCGGCCGACATG |
| miRC03 | AGTGGTGGCAAGGATGAGCAA | ATTCTAGAGGCCGAGGCGGCCGACATG |
| miRC04 | TTTGGAATGATTTGTTGATGA | ATTCTAGAGGCCGAGGCGGCCGACATG |
| miRC05 | AAGATCTCCCATTGCATCTGA | ATTCTAGAGGCCGAGGCGGCCGACATG |
| miRC06 | TTTTTTGGTTATGGTTGGCTG | ATTCTAGAGGCCGAGGCGGCCGACATG |
| miRC07 | CTCAAGAAAGCTGTGGGAAAA | ATTCTAGAGGCCGAGGCGGCCGACATG |
| miRC07* | TTTCCACATCTTTCTTGAACT | ATTCTAGAGGCCGAGGCGGCCGACATG |
| miRC08 | AGAAGAACAAGTAGACTGAGC | ATTCTAGAGGCCGAGGCGGCCGACATG |
| miRC09 | TTATATAGGCTTTGAGGATGGA | ATTCTAGAGGCCGAGGCGGCCGACATG |
| miRC10 | TTTTAAAAAGGTTCGTCATTC | ATTCTAGAGGCCGAGGCGGCCGACATG |
| miRC11 | CCGTGACAAGTGGTATCAGAG | ATTCTAGAGGCCGAGGCGGCCGACATG |
| miRC12 | TCTGAAGTTTGAAGAGCTGTG | ATTCTAGAGGCCGAGGCGGCCGACATG |
| miRC12* | AGAGCAATCTACGAACAACAGGAA | ATTCTAGAGGCCGAGGCGGCCGACATG |
| miRC13 | TTGGCTTGGAGATGGATCATT | ATTCTAGAGGCCGAGGCGGCCGACATG |
| miRC14 | TTGGCTTGGAGATGGATCATT | ATTCTAGAGGCCGAGGCGGCCGACATG |
| miRC15 | TCAATTTGAGAGCTGGAAGAA | ATTCTAGAGGCCGAGGCGGCCGACATG |
| miRC16 | ATATTGGTAAATGAATGTTCG | ATTCTAGAGGCCGAGGCGGCCGACATG |
| miRC17 | AATTTCTTATGTTCATGATTG | ATTCTAGAGGCCGAGGCGGCCGACATG |
| miRC18 | AAGAGCAGTTGAACTGAAGCA | ATTCTAGAGGCCGAGGCGGCCGACATG |
| miRC19 | TCTGTCGCAGGAGAGATGATGC | ATTCTAGAGGCCGAGGCGGCCGACATG |
| miRC20 | GGAATGGGCTGATTGGGATA | ATTCTAGAGGCCGAGGCGGCCGACATG |
| miRC20* | TTCCCAATGCCGCCCATTCCAA | ATTCTAGAGGCCGAGGCGGCCGACATG |
| miRC21 | CCAAGAGGGTGGAGTTCAGAT | ATTCTAGAGGCCGAGGCGGCCGACATG |
| miRC21* | CTGAACTCTCTCCCTCATGGCC | ATTCTAGAGGCCGAGGCGGCCGACATG |
| miRC22 | CTAAATTGCTTCGGGTCCTGC | ATTCTAGAGGCCGAGGCGGCCGACATG |
| miRC22* | AGGAGATGAGGTATGTTTACAT | ATTCTAGAGGCCGAGGCGGCCGACATG |
| miRC23 | AAACATGAGTCTGGACCTTGA | ATTCTAGAGGCCGAGGCGGCCGACATG |
| miRC24 | AAACATGAGTCTGGACCTTGA | ATTCTAGAGGCCGAGGCGGCCGACATG |
| miRC25 | TCTGTTTTCACTCTCATTAAG | ATTCTAGAGGCCGAGGCGGCCGACATG |
| miRC25* | TAGTGAGAATGAGTTGGGGAAG | ATTCTAGAGGCCGAGGCGGCCGACATG |
| miRC26 | TCGGAGAAGTGTGATGTGTAT | ATTCTAGAGGCCGAGGCGGCCGACATG |
| miRC27 | ATACCATGTGGAAAAGAGGAATC | ATTCTAGAGGCCGAGGCGGCCGACATG |
| miRC28 | ATTGGCAGAATATTCAAGGTTT | ATTCTAGAGGCCGAGGCGGCCGACATG |
| miRC29 | TTATTAGGAGGACATTTAGGTAT | ATTCTAGAGGCCGAGGCGGCCGACATG |
| miRC30 | TGCGGGTGGAAGAGAAGGAAG | ATTCTAGAGGCCGAGGCGGCCGACATG |
| miRC31 | TTCCTGCGGTTTCTCGGCGAC | ATTCTAGAGGCCGAGGCGGCCGACATG |
| miRC32 | TTTTCCTATGATTTCTTGGCA | ATTCTAGAGGCCGAGGCGGCCGACATG |
| miRC32* | CTGGGAAAGCGTGGGAAAACA | ATTCTAGAGGCCGAGGCGGCCGACATG |
| miRC33 | TTCCTATCGTTCCCGGGATTT | ATTCTAGAGGCCGAGGCGGCCGACATG |
| miRC34 | TGACCGGCTCTTATCTCTCATG | ATTCTAGAGGCCGAGGCGGCCGACATG |
| miRC34* | TGAAGATAAAGAGTCTCGTCTGG | ATTCTAGAGGCCGAGGCGGCCGACATG |
| miRC35 | GGAATGGATGGCATGGGAACCA | ATTCTAGAGGCCGAGGCGGCCGACATG |
| miRC36 | TGAGTAGTGGACTATCGCATG | ATTCTAGAGGCCGAGGCGGCCGACATG |
| miRC36* | TGAGATAAGTCTGCTGCTCCAT | ATTCTAGAGGCCGAGGCGGCCGACATG |
| miRC37 | TGGATGCATGTAGCTTGTCAA | ATTCTAGAGGCCGAGGCGGCCGACATG |
| miRC37* | GACAAGTTACATACATCCAAG | ATTCTAGAGGCCGAGGCGGCCGACATG |
| miRC38 | TCCTTCGGCGTCGGCAAATCC | ATTCTAGAGGCCGAGGCGGCCGACATG |
| miRC39 | AAGGGTTTCTCACAGAGTTTA | ATTCTAGAGGCCGAGGCGGCCGACATG |
| miRC39* | AGCTCTGTTGGACTCTCTTTG | ATTCTAGAGGCCGAGGCGGCCGACATG |
| miRC40 | GAGGAGAATGTAGTGGGGTTA | ATTCTAGAGGCCGAGGCGGCCGACATG |
| miRC41 | CTTTGATCAGATATTGGATTG | ATTCTAGAGGCCGAGGCGGCCGACATG |
| miRC41* | AGCAGAGTTTGATAGAGGGC | ATTCTAGAGGCCGAGGCGGCCGACATG |
| miRC42 | AATGACATGAGTTGGAACTAA | ATTCTAGAGGCCGAGGCGGCCGACATG |
| miRC43 | GTTGGAAGCCGGTGGGGGACC | ATTCTAGAGGCCGAGGCGGCCGACATG |
| miRC44 | GTTGGAAGCCGGTGGGGGACC | ATTCTAGAGGCCGAGGCGGCCGACATG |
| miRC45 | GTTGGAAGCCGGTGGGGGACC | ATTCTAGAGGCCGAGGCGGCCGACATG |
| miRC46 | GTTGGAAGTCGGTGGGGGAAC | ATTCTAGAGGCCGAGGCGGCCGACATG |
| miRC47 | GGCGATTGTAAATATGGGTAA | ATTCTAGAGGCCGAGGCGGCCGACATG |
| miRC48 | TCTAGATTTGGAAGTAGGTCA | ATTCTAGAGGCCGAGGCGGCCGACATG |
| miRC49 | GTTGGAAGTCGGTGGGGGACC | ATTCTAGAGGCCGAGGCGGCCGACATG |
| miRC50 | GTTGGAAGCCGGTGGGGGACC | ATTCTAGAGGCCGAGGCGGCCGACATG |
| miRC51 | TGGGCTTGTGGAGAAGAAAGTGA | ATTCTAGAGGCCGAGGCGGCCGACATG |
| miRC52 | CATGGGCGGTTTGGTAAGAGG | ATTCTAGAGGCCGAGGCGGCCGACATG |
| miRC52* | TCTTACCAACACCTCCCATTCC | ATTCTAGAGGCCGAGGCGGCCGACATG |
| miRC53 | GGTATGGGAGGATTGGGGAGA | ATTCTAGAGGCCGAGGCGGCCGACATG |
| miRC53* | TTCCCAAGACCCCCCATGCCAA | ATTCTAGAGGCCGAGGCGGCCGACATG |
| miRC54 | TCATACCTCGATCTTCGGTTTC | ATTCTAGAGGCCGAGGCGGCCGACATG |
| miRC54* | AATCTGAGATCGAGAATGAAA | ATTCTAGAGGCCGAGGCGGCCGACATG |
| miRC55 | ATTCGAACTCAAGACTAAGGT | ATTCTAGAGGCCGAGGCGGCCGACATG |
| miRC56 | GAAGCTCTTGAGGGGGACTG | ATTCTAGAGGCCGAGGCGGCCGACATG |
| miRC56* | ACTCTCCCTCAAGGGCTTCTG | ATTCTAGAGGCCGAGGCGGCCGACATG |
| miRC57 | AGGTGTAGATGCAAGTGCAGA | ATTCTAGAGGCCGAGGCGGCCGACATG |
| miRC58 | TTTAATTTACTAGAGATCTCT | ATTCTAGAGGCCGAGGCGGCCGACATG |
| miRC59 | GGAGTGAAATTGCAGTGACGG | ATTCTAGAGGCCGAGGCGGCCGACATG |
| miRC60 | TCAGCAGGAATTGGACCAGAA | ATTCTAGAGGCCGAGGCGGCCGACATG |
| miRC61 | ACAGTAGGAAATTGAAAGAGA | ATTCTAGAGGCCGAGGCGGCCGACATG |
| miRC61* | TCTTTCATTTTCCTACTTTTT | ATTCTAGAGGCCGAGGCGGCCGACATG |
| miRC62 | AAAGGCGAAGAAAAAGAAGATA | ATTCTAGAGGCCGAGGCGGCCGACATG |
| miRC63 | AATATGGAGGACTGTGTTCTT | ATTCTAGAGGCCGAGGCGGCCGACATG |
| miRC63* | GAACTCAGTTCCGGTACCATCTTCA | ATTCTAGAGGCCGAGGCGGCCGACATG |
| miRC64 | TTGGATTCGCGCACAAACTCG | ATTCTAGAGGCCGAGGCGGCCGACATG |
| miRC65 | TTGGATTCGCGCACAAACTCG | ATTCTAGAGGCCGAGGCGGCCGACATG |
| miRC66 | CAGCAGTTGCTATTGTGGTTG | ATTCTAGAGGCCGAGGCGGCCGACATG |
| miRC67 | AGAAGAGAGAGAGTACAGCTA | ATTCTAGAGGCCGAGGCGGCCGACATG |
| miRC68 | TGGTACCAGGAGGGCAACTGTC | ATTCTAGAGGCCGAGGCGGCCGACATG |
| miRC68* | TGTTGCCCTCCTGGTACCATC | ATTCTAGAGGCCGAGGCGGCCGACATG |
| miRC69 | TCAAGGGTCGAACGGCTTTGC | ATTCTAGAGGCCGAGGCGGCCGACATG |
| miRC70 | TTATGTGAGTGTTCGGCAAATC | ATTCTAGAGGCCGAGGCGGCCGACATG |
| miRC71 | TTAGATGATCATCAACAAACA | ATTCTAGAGGCCGAGGCGGCCGACATG |
| miRC71* | TTTTGTTGCTGGTCATCTAGTC | ATTCTAGAGGCCGAGGCGGCCGACATG |
| miRC72 | TGCTTATTAGGTCTGCTGGCA | ATTCTAGAGGCCGAGGCGGCCGACATG |
| miRC73 | TCAAAAGAGAAAATGTGGATG | ATTCTAGAGGCCGAGGCGGCCGACATG |
| miRC73* | TCCATCTTCTCTCTTTTTACA | ATTCTAGAGGCCGAGGCGGCCGACATG |
| miRC74 | TCGCAGGAGAGATGACGCCGT | ATTCTAGAGGCCGAGGCGGCCGACATG |
| vv-miR156d | TGACAGAAGAGAGTGAGCAC | ATTCTAGAGGCCGAGGCGGCCGACATG |
| vv-miR160a | TCCTAGTTGGCATCAGAGGAG | ATTCTAGAGGCCGAGGCGGCCGACATG |
| vv-miR162 | TCGATAAACCTCTGCATCCAG | ATTCTAGAGGCCGAGGCGGCCGACATG |
| vv-miR164d | TGGAGAAGCAGGGCACGTGCA | ATTCTAGAGGCCGAGGCGGCCGACATG |
| vv-miR166h | TCGGACCAGGCTTCATTCCCC | ATTCTAGAGGCCGAGGCGGCCGACATG |
| vv-miR167a | TGAAGCTGCCAGCATGATCTGG | ATTCTAGAGGCCGAGGCGGCCGACATG |
| vv-miR168 | TCGCTTGGTGCAGGTCGGGAA | ATTCTAGAGGCCGAGGCGGCCGACATG |
| vv-miR169d | CAGCCAAGAATGATTTGCCGG | ATTCTAGAGGCCGAGGCGGCCGACATG |
| vv-miR171d | TCATTGAGTGCAGCGTTGATG | ATTCTAGAGGCCGAGGCGGCCGACATG |
| vv-miR172d | AGAATCTTGATGATGCTGCAT | ATTCTAGAGGCCGAGGCGGCCGACATG |
| vv-miR319c | ATTGAATGATGCGGGAGACAA | ATTCTAGAGGCCGAGGCGGCCGACATG |
| vv-miR390 | AAGCTCAGGAGGGATAGCGCC | ATTCTAGAGGCCGAGGCGGCCGACATG |
| vv-miR393a | ATCATGCTATCCCTTAGGAAC | ATTCTAGAGGCCGAGGCGGCCGACATG |
| vv-miR394b | TATTGGCATTCTGTCCACCTCC | ATTCTAGAGGCCGAGGCGGCCGACATG |
| vv-miR395f | CACTGAAGTGTTTGGGGGAAC | ATTCTAGAGGCCGAGGCGGCCGACATG |
| vv-miR396a | CTCAAGAAAGCTGTGGGAGG | ATTCTAGAGGCCGAGGCGGCCGACATG |
| vv-miR397a | TCATTGAGTGCAGCGTTGATG | ATTCTAGAGGCCGAGGCGGCCGACATG |
| vv-miR398a | CAAGGGAGTGGCACCTGAGAACA | ATTCTAGAGGCCGAGGCGGCCGACATG |
| vv-miR399a | GTGTGATTCTCCTTTGGCAGA | ATTCTAGAGGCCGAGGCGGCCGACATG |
| vv-miR403e | TTAGATTCACGCACAAACTCG | ATTCTAGAGGCCGAGGCGGCCGACATG |
| vv-miR408 | ACGGGGACGAGGTAGTGCATG | ATTCTAGAGGCCGAGGCGGCCGACATG |
| vv-miR477 | TCCCTCAAAGGCTTCCAATTT | ATTCTAGAGGCCGAGGCGGCCGACATG |
| vv-miR479 | TGTGGTATTGGTTCGGCTCATC | ATTCTAGAGGCCGAGGCGGCCGACATG |
| vv-miR482 | AATTGGAGAGTAGGAAAGCTT | ATTCTAGAGGCCGAGGCGGCCGACATG |
| vv-miR535c | TGACAACGAGAGAGAGCACGC | ATTCTAGAGGCCGAGGCGGCCGACATG |
| vv-miR828a | AGATGCTCATTTGAGGAAGCAA | ATTCTAGAGGCCGAGGCGGCCGACATG |
