# Supplementary material for: Grapevine microRNAs responsive to exogenous gibberellin
Source: BMC Genomics. 2014 Feb 8;15:111. doi: 10.1186/1471-2164-15-111 (PMC3937062; doi:10.1186/1471-2164-15-111)
Supplement: Additional file 4 — Primer sequences of qRT-PCR validated novel miRNAs. [file 1471-2164-15-111-S4.doc]

| Table S3 List of predicted target genes of novel miRNAs identified in grapevines | | | | |
| --- | --- | --- | --- | --- |
| miRNA ID | Target genes | Orthologous targets | Target functions | Other plants with orthologs |
| Vv-miRC03 | *GSVIVT01000665001* | XM_002304152 | cc-nbs-lrr resistance protein | *Populus trichocarpa* |
|  | *GSVIVT01000842001* | XM_003599066 | hypothetical protein | *Medicago truncatula* |
|  | *GSVIVT01000871001* | XM_002297700 | cc-nbs-lrr resistance protein | *Populus trichocarpa* |
|  | *GSVIVT01001446001* | XM_002519323 | Disease resistance protein RPS2 | *Ricinus communis* |
|  | *GSVIVT01001448001* | XM_002332904 | cc-nbs-lrr resistance protein | *Populus trichocarpa* |
|  | *GSVIVT01001453001* | Unknow |  |  |
|  | *GSVIVT01001457001* | XM_002297689 | cc-nbs-lrr resistance protein | *Populus trichocarpa* |
|  | *GSVIVT01001459001* | XM_002304749 | cc-nbs-lrr resistance protein | *Populus trichocarpa* |
|  | *GSVIVT01001473001* | XM_002334632 | nbs-lrr resistance protein | *Populus trichocarpa* |
|  | *GSVIVT01004013001* | Unknow |  |  |
|  | *GSVIVT01004021001* | Unknow |  |  |
|  | *GSVIVT01004035001* | Unknow |  |  |
|  | *GSVIVT01004038001* | Unknow |  |  |
|  | *GSVIVT01004040001* | Unknow |  |  |
|  | *GSVIVT01004061001* | XM_002299886 | cc-nbs-lrr resistance protein | *Populus trichocarpa* |
|  | *GSVIVT01004064001* | Unknow |  |  |
|  | *GSVIVT01005316001* | Unknow |  |  |
|  | *GSVIVT01005320001* | XM_002299886 | cc-nbs-lrr resistance protein | *Populus trichocarpa* |
|  | *GSVIVT01012407001* | XM_002442373 | hypothetical protein | *Sorghum bicolor* |
|  | *GSVIVT01012421001* | XM_002442373 | hypothetical protein | *Sorghum bicolor* |
|  | *GSVIVT01013310001* | Unknow |  |  |
|  | *GSVIVT01022829001* | Unknow |  |  |
|  | *GSVIVT01022835001* | Unknow |  |  |
|  | *GSVIVT01022837001* | Unknow |  |  |
|  | *GSVIVT01022856001* | Unknow |  |  |
|  | *GSVIVT01022859001* | Unknow |  |  |
|  | *GSVIVT01023312001* | Unknow |  |  |
|  | *GSVIVT01031991001* | Unknow |  |  |
|  | *GSVIVT01031992001* | Unknow |  |  |
|  | *GSVIVT01032000001* | Unknow |  |  |
| Vv-miRC06 | *GSVIVT01027473001* | Unknow |  |  |
| Vv-miRC07 | *GSVIVT01012147001* | XM_002326404 | predicted protein | *Populus trichocarpa* |
| Vv-miRC11 | *GSVIVT01020995001* | XM_002328864 | predicted protein | *Populus trichocarpa* |
| Vv-miRC12 | *GSVIVT01004013001* | XM_002330810 | predicted protein | *Populus trichocarpa* |
|  | *GSVIVT01020514001* | Unknow |  |  |
|  | *GSVIVT01022864001* | Unknow |  |  |
|  | *GSVIVT01023999001* | Unknow |  |  |
| Vv-miRC13 | *GSVIVT01024172001* | Unknow |  |  |
|  | *GSVIVT01034151001* | XM_002309029 | predicted protein | *Populus trichocarpa* |
| Vv-miRC14 | *GSVIVT01024172001* | XM_002512951 | ATP binding protein | *Ricinus* |
|  | *GSVIVT01034151001* | NM_180423 | hydrolase domain-containing protein | *Arabidopsis thaliana* |
| Vv-miRC15 | *GSVIVT01000639001* | XM_002299886 | cc-nbs-lrr resistance protein | *Populus* |
|  | *GSVIVT01000646001* | XM_002299886 | cc-nbs-lrr resistance protein | *Populus* |
|  | *GSVIVT01000656001* | XM_002299886 | cc-nbs-lrr resistance protein | *Populus* |
|  | *GSVIVT01000657001* | XM_002299886 | cc-nbs-lrr resistance protein | *Populus* |
|  | *GSVIVT01031992001* | Unknown |  |  |
| Vv-miRC16 | *GSVIVT01000871001* | DQ513206 | disease resistance protein | *Populus* |
|  | *GSVIVT01001473001* | EF613535 | disease resistance protein | *Solanum demissum* |
| Vv-miRC21 | *GSVIVT01016754001* | Unknown |  |  |
| Vv-miRC23 | *GSVIVT01018564001* | Unknown |  |  |
|  | *GSVIVT01026728001* | XM_002334660 | nbs-lrr resistance protein | *Populus* |
|  | *GSVIVT01026747001* | XM_002334660 | nbs-lrr resistance protein | *Populus* |
|  | *GSVIVT01026750001* | XM_002304553 | nbs-lrr resistance protein | *Populus* |
|  | *GSVIVT01026825001* | XM_002524176 | disease resistance protein | *Ricinus* |
| Vv-miRC24 | *GSVIVT01018564001* | XM_002334660 | nbs-lrr resistance protein | *Populus* |
|  | *GSVIVT01026728001* | XM_002524176 | disease resistance protein | *Ricinus* |
|  | *GSVIVT01026747001* | XM_002304553 | nbs-lrr resistance protein | *Populus* |
| Vv-miRC28 | *GSVIVT01024479001* | NM_120826 | glutamate dehydrogenase | *Arabidopsis thaliana* |
| Vv-miRC38 | *GSVIVT01023141001* | NM_124362 | ATP-dependent Clp protease | *Arabidopsis thaliana* |
|  | *GSVIVT01037101001* | NM_103063 | ATP-dependent Clp protease | *Arabidopsis thaliana* |
| Vv-miRC40 | *GSVIVT01025804001* | Unknown |  |  |
| Vv-miRC47 | *GSVIVT01014125001* | XM_002332988 | BED finger-nbs resistance protein | *Populus* |
| Vv-miRC48 | *GSVIVT01014594001* | XM_002331747 | cc-nbs-lrr resistance protein | *Populus* |
|  | *GSVIVT01014595001* | NM_118853 | domain-containing disease resistance protein | *Arabidopsis thaliana* |
|  | *GSVIVT01014596001* | NM_118853 | domain-containing disease resistance protein | *Arabidopsis thaliana* |
|  | *GSVIVT01038056001* | NM_118853 | domain-containing disease resistance protein | *Arabidopsis thaliana* |
| Vv-miRC56 | *GSVIVT01017984001* | unknown |  |  |
|  | *GSVIVT01026952001* | NM_201966 | ATP-dependent helicase | *Arabidopsis thaliana* |
|  | *GSVIVT01035357001* | NM_179350 | Protein phosphatase | *Arabidopsis thaliana* |
| Vv-miRC57 | *GSVIVT01000587001* | NM_101188 | actin-related protein | *Arabidopsis thaliana* |
|  | *GSVIVT01006040001* | AB616659 | disease resistance protein | *Oryza sativa* |
|  | *GSVIVT01011907001* | unknown |  |  |
| Vv-miRC59 | *GSVIVT01019259001* | FJ708783 | leucine-rich repeat receptor-like protein kinase | *Arabidopsis thaliana* |
|  | *GSVIVT01035304001* | NM_101584 | receptor-like protein kinase | *Arabidopsis thaliana* |
| Vv-miRC59 | *GSVIVT01035308001* | [XM_002335992.1](http://www.ncbi.nlm.nih.gov/nucleotide/224143343?report=genbank&log$=nucltop&blast_rank=24&RID=EB09WMF2013) | predicted protein | *Populus trichocarpa* |
|  | *GSVIVT01035312001* | XM_002456291.1 | hypothetical protein | *Sorghum bicolor* |
|  | *GSVIVT01035313001* | XM_002335992.1 | predicted protein | *Populus trichocarpa* |
|  | *GSVIVT01035314001* | XM_002516487.1 | serine-threonine protein kinase | *Ricinus communis* |
| Vv-miRC60 | *GSVIVT01015298001* | [XM_002514174.1](http://www.ncbi.nlm.nih.gov/nucleotide/255546320?report=genbank&log$=nuclalign&blast_rank=11&RID=EZDMYCF001R) | serine-threonine protein kinase | *Ricinus communis* |
|  | *GSVIVT01019259001* | [XM_003611459.1](http://www.ncbi.nlm.nih.gov/nucleotide/357482440?report=genbank&log$=nuclalign&blast_rank=73&RID=EZEY9WPC01R) | Receptor-like protein kinase | *Medicago truncatula* |
|  | *GSVIVT01023658001* | [XM_002528360.1](http://www.ncbi.nlm.nih.gov/nucleotide/255574999?report=genbank&log$=nuclalign&blast_rank=5&RID=EZF8SJYK01R) | Receptor protein kinase CLAVATA1 precursor | *Ricinus communis* |
|  | *GSVIVT01035303001* | [XM_002872144.1](http://www.ncbi.nlm.nih.gov/nucleotide/297808612?report=genbank&log$=nuclalign&blast_rank=35&RID=EZFH95US01R) | lyrata leucine-rich repeat family protein | *Arabidopsis lyrata subsp.* |
|  | *GSVIVT01035304001* | NM_122494.3 | thaliana Protein kinase family protein with leucine-rich repeat domain | *Arabidopsis* |
|  | *GSVIVT01035311001* | XM_003603596.1 | Receptor-like protein kinase | *Medicago truncatula* |
|  | *GSVIVT01035313001* | [XM_003619738.1](http://www.ncbi.nlm.nih.gov/nucleotide/357498994?report=genbank&log$=nuclalign&blast_rank=51&RID=EZH4W1N301R) | Receptor-like protein kinase | *Medicago truncatula* |
|  | *GSVIVT01035314001* | [XM_002516487.1](http://www.ncbi.nlm.nih.gov/nucleotide/255550969?report=genbank&log$=nuclalign&blast_rank=26&RID=EZH93H3201R) | serine-threonine protein kinase | *Ricinus communis* |
|  | *GSVIVT01035367001* | [XM_002442894.1](http://www.ncbi.nlm.nih.gov/nucleotide/242085027?report=genbank&log$=nuclalign&blast_rank=78&RID=EZHD25DH01R) | hypothetical protein | *Sorghum bicolor* |
| Vv-miRC62 | *GSVIVT01017189001* | [XM_002527285.1](http://www.ncbi.nlm.nih.gov/nucleotide/255572796?report=genbank&log$=nuclalign&blast_rank=32&RID=EZHN7X8V015) | ATP-binding cassette transporter | *Ricinus communis* |
|  | *GSVIVT01018830001* | [XM_003629310.1](http://www.ncbi.nlm.nih.gov/nucleotide/357518138?report=genbank&log$=nuclalign&blast_rank=7&RID=EZHTCR1901R) | Coiled-coil domain-containing protein 90B | *Medicago truncatula* |
| Vv-miRC63 | *GSVIVT01001633001* | unknown |  |  |
|  | *GSVIVT01001636001* | unknown |  |  |
|  | *GSVIVT01001651001* | [XM_002533349.1](http://www.ncbi.nlm.nih.gov/nucleotide/255585398?report=genbank&log$=nuclalign&blast_rank=82&RID=EZJ8AV9J014) | ankyrin repeat-containing protein | *Ricinus communis* |
|  | *GSVIVT01010727001* | unknown |  |  |
|  | *GSVIVT01010731001* | unknown |  |  |
|  | *GSVIVT01013569001* | [|XM_002533349.1](http://www.ncbi.nlm.nih.gov/nucleotide/255585398?report=genbank&log$=nuclalign&blast_rank=75&RID=EZJNM5JN01R) | ankyrin repeat-containing protein | *Ricinus communis* |
|  | *GSVIVT01013583001* | [XM_002516972.1](http://www.ncbi.nlm.nih.gov/nucleotide/255551946?report=genbank&log$=nuclalign&blast_rank=81&RID=EZJTBZRS015) | ankyrin repeat-containing protein | *Ricinus communis* |
| Vv-miRC67 | *GSVIVT01031981001* | [NM_101804.3](http://www.ncbi.nlm.nih.gov/nucleotide/42562176?report=genbank&log$=nuclalign&blast_rank=28&RID=EZJYB8JU014) | DNA glycosylase domain-containing protein | *Arabidopsis thaliana* |
|  | *GSVIVT01033519001* | [XM_002532297.1|](http://www.ncbi.nlm.nih.gov/nucleotide/255583153?report=genbank&log$=nuclalign&blast_rank=5&RID=EZK3B62S01R) | LIGULELESS1 protein | *Ricinus communis* |
| Vv-miRC69 | *GSVIVT01000040001* | [XM_002512323.1](http://www.ncbi.nlm.nih.gov/nucleotide/255542611?report=genbank&log$=nuclalign&blast_rank=8&RID=EZK7FB04014) | hypothetical protein | *Ricinus communis* |
| Vv-miRC71 | *GSVIVT01011059001* | [XM_002524749.1](http://www.ncbi.nlm.nih.gov/nucleotide/255567631?report=genbank&log$=nuclalign&blast_rank=7&RID=EZKBPM7U01R) | conserved hypothetical protein | *Ricinus communis* |
|  | *GSVIVT01011553001* | [XM_001853144.1](http://www.ncbi.nlm.nih.gov/nucleotide/170048533?report=genbank&log$=nuclalign&blast_rank=80&RID=EZKEGF9Z015) | conserved hypothetical protein | *Culex quinquefasciatus* |
| Vv-miRC72 | *GSVIVT01025339001* | [XM_002513397.1](http://www.ncbi.nlm.nih.gov/nucleotide/255544763?report=genbank&log$=nuclalign&blast_rank=26&RID=EZKKYPXW014) | lipoxygenase | *Ricinus communis* |
|  | *GSVIVT01025340001* | [FJ864335.1](http://www.ncbi.nlm.nih.gov/nucleotide/227345103?report=genbank&log$=nuclalign&blast_rank=78&RID=EZKV7FX801R) | nigra lipoxygenase | *Populus simonii x Populus* |
|  | *GSVIVT01025341001* | [XM_002513182.1](http://www.ncbi.nlm.nih.gov/nucleotide/255544331?report=genbank&log$=nuclalign&blast_rank=30&RID=EZM17R4K01R) | lipoxygenase | *Ricinus communis* |
|  | *GSVIVT01025342001* | [XM_002875668.1](http://www.ncbi.nlm.nih.gov/nucleotide/297815661?report=genbank&log$=nuclalign&blast_rank=63&RID=EZM9RYP901R) | lyrata hypothetical protein | *Arabidopsis lyrata subsp.* |
| Vv-miRC73 | *GSVIVT01012214001* | [XM_002875163.1](http://www.ncbi.nlm.nih.gov/nucleotide/297814651?report=genbank&log$=nuclalign&blast_rank=7&RID=EZMB018X014) | unknown | *Arabidopsis lyrata subsp.* |
